# Reinforcing the supply chain of umifenovir and other antiviral drugs with retrosynthetic software

**DOI:** 10.1038/s41467-021-27547-3

**Published:** 2021-12-16

**Authors:** Yingfu Lin, Zirong Zhang, Babak Mahjour, Di Wang, Rui Zhang, Eunjae Shim, Andrew McGrath, Yuning Shen, Nadia Brugger, Rachel Turnbull, Sarah Trice, Shashi Jasty, Tim Cernak

**Affiliations:** 1 Department of Medicinal Chemistry, University of Michigan, Ann Arbor, MI 48109 USA; 2 Department of Chemistry, University of Michigan, Ann Arbor, MI 48109 USA; 3 MilliporeSigma, Burlington, MA 01803 USA; 4 Present Address: Entos, Inc., San Diego, CA 92037 USA

**Keywords:** Chemical synthesis, Cheminformatics

## Abstract

The global disruption caused by the 2020 coronavirus pandemic stressed the supply chain of many products, including pharmaceuticals. Multiple drug repurposing studies for COVID-19 are now underway. If a winning therapeutic emerges, it is unlikely that the existing inventory of the medicine, or even the chemical raw materials needed to synthesize it, will be available in the quantities required. Here, we utilize retrosynthetic software to arrive at alternate chemical supply chains for the antiviral drug umifenovir, as well as eleven other antiviral and anti-inflammatory drugs. We have experimentally validated four routes to umifenovir and one route to bromhexine. In one route to umifenovir the software invokes conversion of six C–H bonds into C–C bonds or functional groups. The strategy we apply of excluding known starting materials from search results can be used to identify distinct starting materials, for instance to relieve stress on existing supply chains.

## Abstract (web summary)

COVID-19 has exposed the fragility of supply chains, particularly for goods that are essential or may suddenly become essential, such as repurposed pharmaceuticals. Here the authors develop a methodology to provide routes to pharmaceutical targets that allow low-supply starting materials or intermediates to be avoided, with representative pathways validated experimentally.

## Introduction

In 2020, the scourge of coronavirus highlighted the fragility of diverse supply chains, affecting the world’s pipeline of hand sanitizer^1^, toilet paper^2^, and pharmaceutical starting materials^3^. A diverse array of antiviral and anti-inflammatory drugs was investigated in the hopes that an existing medicine could be repurposed for use against COVID-19^4,5^. The scale of the pandemic^6^ would easily stress the pharmaceutical supply chain^7^. For instance, at the time of our study, the only drug with an emergency use authorization for treating COVID-19 was remdesivir (**1**). While its producer, Gilead Sciences, Inc., ramped up production of **1** significantly, there were only 5000 doses of **1** available when the outbreak began^8^.

We realized that the availability of alternative starting materials to promising synthetic therapeutics could alleviate pressure on supply chains. We reasoned that modern retrosynthetic software could be used to analyze the diverse chemical agents that were being studied clinically and pre-clinically when we initiated our study^4^. For the sake of preparedness, we chose to consider multiple therapeutic synthetic targets simultaneously, even though many would eventually prove to be irrelevant in the war on COVID-19. We focused our experimental attention on umifenovir (**2**), which had been used against SARS-CoV1, and is effective against SARS-CoV2 in vitro^9^. Automated retrosynthesis has already been used to design contingency plans for the investigational COVID-19 therapeutics hydroxychloroquine and remdesivir (**1**)^10^. This reaction-centric study identified novel sequences computationally, but the routes initiate with known starting materials, or from starting materials that added significantly to route length or reagent cost.

We present herein a starting material-centric retrosynthetic analysis of 12 investigational COVID-19 drugs. We identify distinct raw materials that are of comparable cost, and which feature in routes of comparable length to known routes to the 12 diverse targets. From our perspective, it was not detrimental to intercept established synthetic routes to the selected drugs, as long as the overall route had a competitive step-count and initiated with distinct starting materials of a comparable or better price. The simultaneous design of multistep preparative routes to diverse targets, which circumvent the use of established raw materials, presents a considerable data handling challenge that would be a good test of modern computer-assisted synthesis planning software^11–20^. We enlisted the SYNTHIA^TM^ retrosynthesis platform^12,16,20^ to facilitate navigation of requisite parameters including availability, pricing, and novelty of starting materials, route brevity, issues of chemo-, regio-, and stereoselectivity, as well as route visualization, documentation, sharing, and storage. We present herein predicted retrosynthetic routes to twelve diverse COVID-19 therapeutic candidates that initiate, as frequently as possible, with starting materials that are distinct from those used in published or patented syntheses. Few studies of modern retrosynthetic software validate routes experimentally, so it was important for us to realize some of our calculated routes.

## Results and discussion

### Crowd-sourced data collection and automated retrosynthesis

**Fig. 1:**
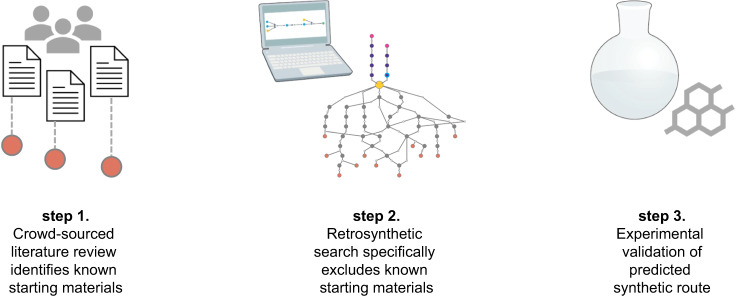
Workflow to identify alternate starting material supply chains. 1. Published starting materials are encoded as SMILES strings, which are 2. excluded from automated retrosynthetic searching. 3. Select routes are validated in the wet lab.

Our study commenced with a crowd-sourcing approach wherein each member of our lab collected all published and patented synthetic routes for one of the drugs in Fig. 1. The routes were then encoded via their simplified molecular-input line-entry system (SMILES) strings. From this dataset we built an interactive route visualizer, available for free at http://covidroutes.cernaklab.com^21^, to facilitate review of existing routes. The concatenated list of starting material SMILES from each target was used as an exclusion criterion in each retrosynthetic search. This approach allowed us to rapidly navigate to novel starting materials. Each search result contained 50 route proposals, and the user-defined search heuristic was generally set to minimize starting material cost. A single search heuristic worked for most targets, but occasionally, the preference for minimized cost reagents would result in proposed routes with more reaction steps than desired. In these cases, the search heuristic was modified by relaxing the preference for reagent cost and increasing the software’s beam search width. Predicted routes were manually reviewed for step count, synthetic feasibility, and ease of execution of proposed reactions on the multikilogram scale, for instance by biasing towards routes that minimized the use of cryogenic cooling or pyrophoric reagents. The final heuristic used for each target is shown in the Supplementary Information.

**Fig. 2:**
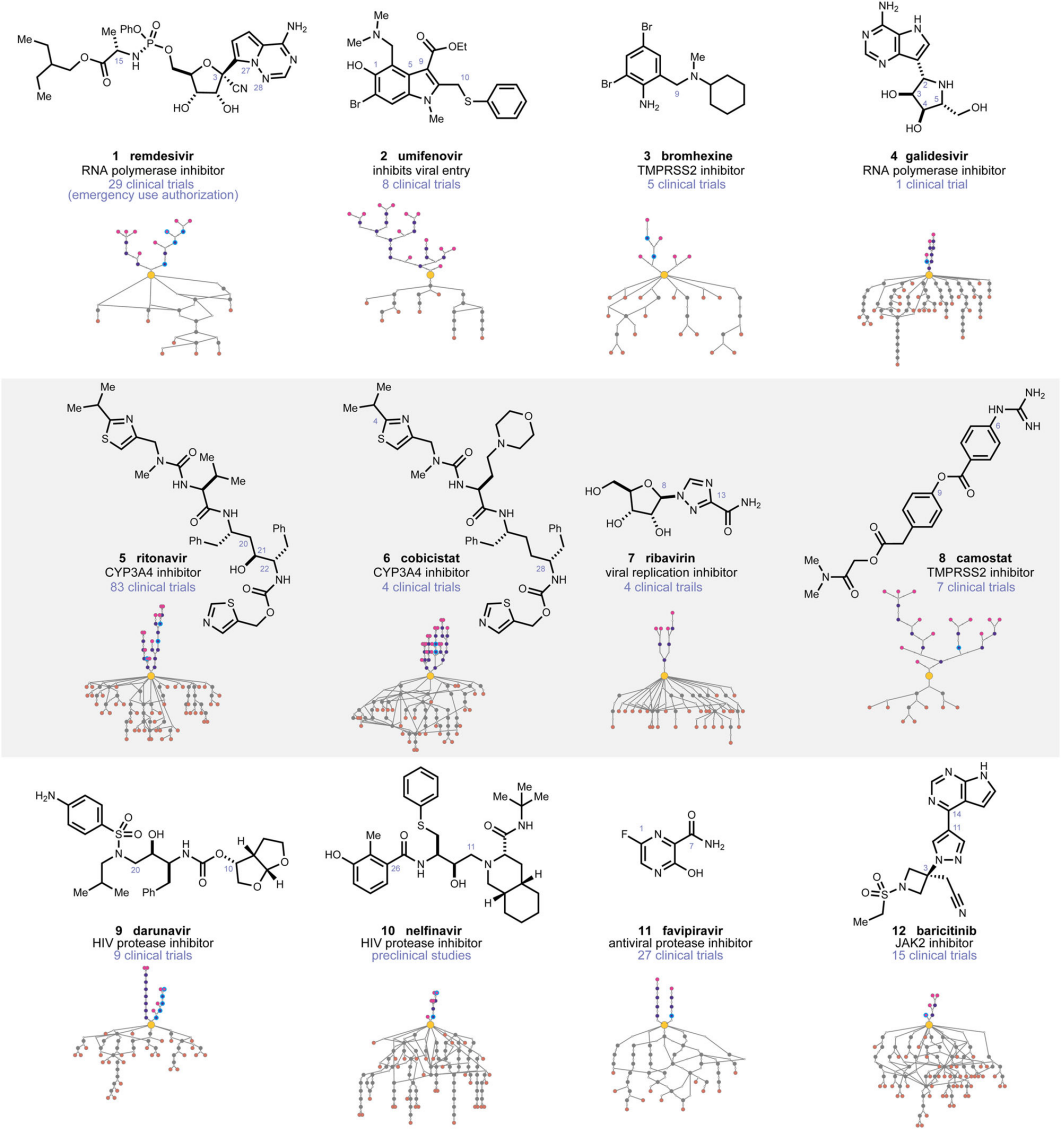
Therapeutics being evaluated for COVID-19 and their retrosynthetic analysis networks. The number of clinical trials is based on search results for all listed trials, completed, active, or planned, found on www.clinicaltrials.gov (accessed July 7th, 2020). For the route networks, the yellow dot at the center is the target molecule. Routes in grey and orange below the target are published, and the routes in purple and pink above the target are routes predicted. Intersection nodes in literature routes reflect common intermediates or starting materials. A version of this diagram is available as an interactive route visualizer.

**Fig. 3:**
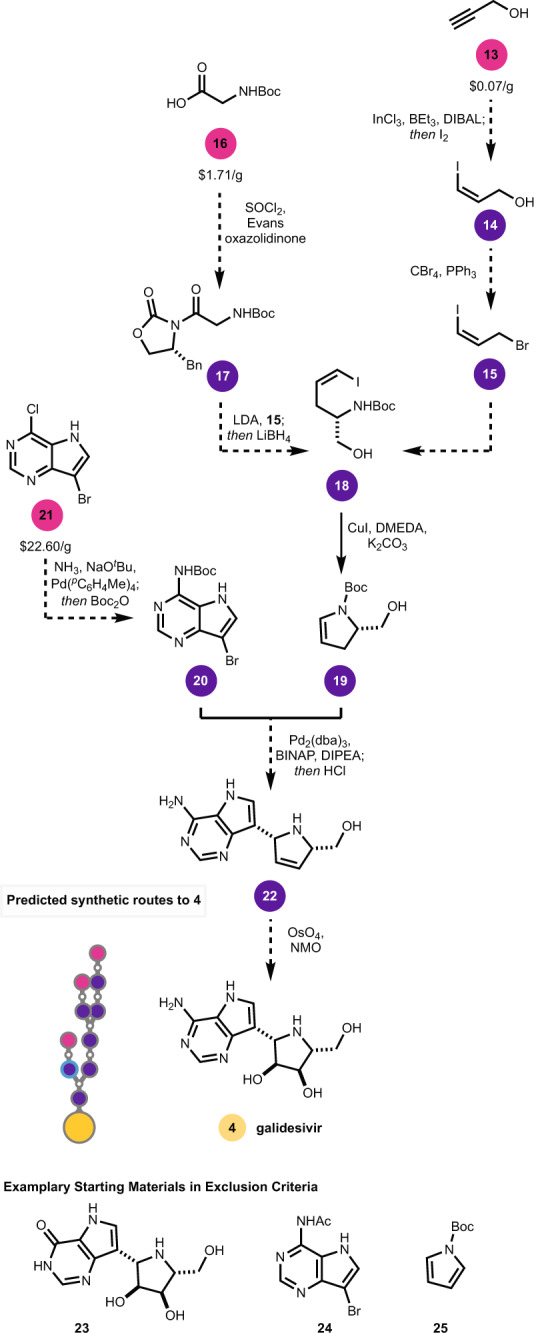
Predicted synthetic sequence to 4. Reaction conditions are proposals from the SYNTHIA^™^ software.

Among small molecules being considered (Fig. 2), we focused on remdesivir (**1**), umifenovir (**2**), bromhexine (**3**), galidesivir (**4**), ritonavir (**5**), cobicistat (**6**), ribavirin (**7**), camostat (**8**), darunavir (**9**), nelfinavir (**10**), favipiravir (**11**), and baricitinib (**12**). In most cases, the proposed route has the same number, or fewer, steps than the established routes, and initiates from distinct starting materials. Our analysis yielded alternate starting material proposals for **1**–**12**, which can relieve pressure on the fine chemical supply chain. Using galidesivir (**4**) as an example (Fig. 3), the software proposed a sequence hinging on a *trans*-hydroiodination of alkyne **13**, an Evans alkylation to form **18**, an Ullman coupling to form **19**, and an enantioselective Heck-coupling to give **22**. The software proposed that the latter reaction mixture could be subjected in situ to hydrochloric acid to remove the Boc-protecting groups in a one-pot operation. Dihydroxylation of **22** would complete the synthesis of **4**. Exemplary starting materials **23**–**25** were excluded from the search based on the appearance of their SMILES strings in published routes to **4**. The algorithm successfully navigated around five established pyrrolopyrimidine starting materials to arrive at **21**, which is cost-competitive with the established nucleobase sources; for instance, the 7-*des*-bromo analog of **21**, 4-chloro-5H-pyrrolo[3,2-d]pyrimidine, is listed at $9.90/g while a 4–OH analog of **21**, 7-bromo-5H-pyrrolo[3,2-d]pyrimidin-4-ol, which is used in the reported synthesis of **4**, is listed at $280/g. On a production scale, all starting material pricing would likely be customized based on competitive bidding, but in any event, the high list price of **21** is comparable to starting material analogs currently described in patents. The proposed use of an Evans auxiliary to produce **18** highlights the software’s desire to select robust chemistry, but this step could likely be replaced with a catalytic protocol to avoid auxiliary use for large-scale production if needed. Indeed, the overall route proposes a variety of catalytic operations. For the production of **1**–**12** on large scale, routes could be found that minimized use of cryogenic conditions, pyrophoric reagents, or expensive catalysts, which were the main biases imposed in our manual review of answer sets beyond route length and starting material cost. For **1**, novel starting materials were identified but the route bore high similarity to known routes, mirroring the automated retrosynthesis findings of the Grzybowski lab for this target^10^. While the software was challenged by esoteric functionalities like a chiral phosphorus atom, the predicted route to **4** discussed above, and those to **2** and **3** discussed below, represent typical outputs.

### Retrosynthesis and experimental syntheses of umifenovir

Umifenovir (**2**), is an antiviral drug developed to combat influenza infections whose use against SARS-CoV1 made it an attractive synthetic target for this study. It is believed that **2** inhibits the entry of viruses into human cells, and the antiviral has been used in many clinical trials as an investigational COVID-19 therapy^9^. Although **2** shows promising in vitro activity against the novel coronavirus, recent clinical results suggest limited efficacy for **2** against COVID-19 in humans^22^. Using the search criteria described above, we arrived at a series of routes to **2** based on the oxidative cyclization of aniline with a β-ketoester^23^. Since it is a commodity chemical, ethyl acetoacetate **27** was not included in the exclusion criteria of our heuristic and appears as a starting material here, although it has been used previously in the synthesis of **2**. Starting materials **28**–**30**, among several others (see Supplementary Information), were excluded. A key theme that separated the predicted routes from the established indole-forming routes, and enabled the use of distinct starting materials, was the incorporation of a Baeyer–Villiger oxidation to utilize an acetyl group as a surrogate to the requisite hydroxyl group at C1. We found this proposal of a Baeyer–Villiger oxidation to be a surprising solution. Among other proposals that were non-obvious to us was the suggested C–H oxidation of an ethyl group where the use of C–H functionalization logic^24–27^ reduces the cost of the starting materials. The software proposed an inefficient three-step sequence to *N*-methylate the indole, whereas published syntheses of **2** reported *N*-methylation directly from the indole with methyl iodide and sodium hydride. We opted to use this one-step precedent instead of the software’s three-step proposal. In another search, a proposed sequence to **2** was initiated with a pre-installed halogen coupling handle, instead of a C–H bond, to enable a related indole formation, but instead invoked a Bamberger rearrangement to functionalize the C–H bond. As described below, these four routes were reduced to experimental practice with only minor modifications to reaction conditions and sequences proposed by the software.

**Fig. 4:**
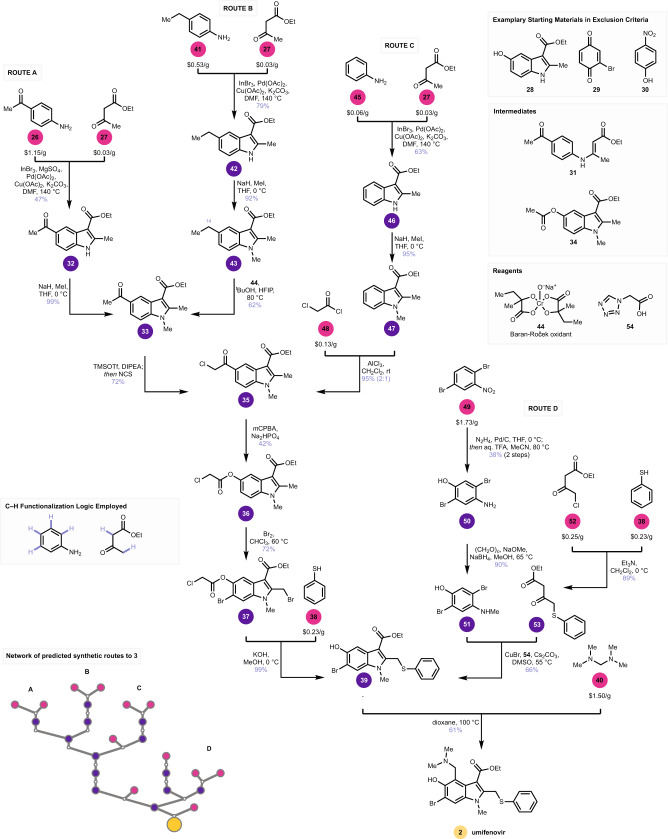
Experimental demonstration of four routes to 2. Each route terminates in inexpensive starting materials. **26**, **41**, **45**, **48**, and **49** have not been used in a published synthesis of **2**. The conversion of **33** into **35**, and the use of starting material **49**, instead of 5-bromo-2-chloro-1-nitrobenzene, were manual modifications to the software’s prediction. See main text for details.

To experimentally validate routes to **2**, we first investigated the proposed indole formation from 1-(4-aminophenyl)ethan-1-one (**26**, $1.15/g) and ethyl acetoacetate (**27**, $0.03/g) using oxidative reaction conditions (Fig. 4 route A). Pretreatment of **26** and **27** with 1 mol% indium(III) bromide, to form **31**, was followed by oxidative cyclization to form **32**. While the published reaction conditions for the suggested reaction^23^ did provide the desired indole **32**, the yield was only 20%. Using magnesium sulfate to promote the formation of **31** improved the yield of **32** to 47%. As described below, other implementations of this reaction gave much higher yields. *N*-Methylation occurred smoothly to produce **33** in 99% yield. An issue was encountered in the experimental realization of the Baeyer–Villiger oxidation using *m*CPBA in that a mixture of oxidation products was obtained. Unstable products we believe to be from oxidation of the indole’s double bond accounted for the bulk of the reacted material, and only traces of **34** were isolated. While the formation of **34** was accurately predicted, the subtle interplay of electronics that govern the preference for the desired Baeyer-Villiger oxidation over the undesired Prilezhaev oxidation could not be teased out by the software, and the best modification of reaction conditions we found yielded small amounts of **34** as a mixture with undesired oxidation products. A literature search on related indoles revealed that the α-chloroketone **35** should be a viable substrate for the Baeyer–Villiger^28^, with the chloro-group acting as a directing group to favor oxidation of the ketone. We thus modified the route and, indeed, chlorination of **33** led to **35**, which underwent selective Baeyer–Villiger oxidation to produce **36**. Subsequent bromination produced **37**, which underwent thioetherification with **38** and in situ saponification to produce **39**. Here, the route intercepts known syntheses of **2** via alkylation with **40**^29^. All intermediates predicted by the software were observed, but a modification to incorporate a chlorine directing group was necessary to achieve usable levels of selectivity in the formation of **36**. This change led us to demonstrate the bromination of **36** to produce **37**, instead of brominating **34**, yet the selective bromination of **34** en route to **39** is a known reaction^30^.

The output of a SYNTHIA^TM^ search is a ranked list of route proposals. Several other computed routes to **2** were also experimentally vetted. One route, based on a variation of the same indole-formation and Baeyer–Villiger sequence described above, proposed a benzylic C–H oxidation of indole **43** (Fig. 4, route B). The indole-synthesis was more productive with **41** than with **26**, yielding **42** in 79% yield. Methylation gave **43** in 92% yield. SYNTHIA^TM^ predicts reaction sequences. Corresponding reaction conditions are recommended based on what was reported in the source literature. While these recommendations work well for a majority of substrates, exact recipes for specific substrates may require user direction. Software-recommended conditions of Oxone^®^ with potassium bromide^31^ for the C–H oxidation of **43** were unsuccessful in our hands. An experimental survey of oxidants revealed the recently disclosed Baran-Roček oxidation^32,33^ could selectively oxidize C14 in 62% yield, thus intercepting the previous route to **2**. While the generation of chromium waste is only viable on small scale, this result validates the proposed C–H functionalization hypothesis.

The direct installation of the chloromethyl ketone via a Friedel–Crafts acylation would provide a concise and alternative route to **2**. Indeed, the software had proposed a route that used a Friedel–Crafts acylation (Fig. 4, route C). This route was intriguing in that it initiated the synthesis from **45**, an exceptionally cheap starting material. While the software proposed a Friedel–Crafts acylation with acetyl chloride, we modified the route to use instead chloroacetyl chloride (**48**, $0.13/g), thus installing the chloride directing group in a single step. Experimentally, oxidative indole-coupling to form **46**, followed by methylation to form **47**, occurred smoothly. Friedel–Crafts acylation of **47** with **48** under influence of aluminum(III) chloride gave **35** and intercepted the other routes. The 2:1 regioselectivity of the acylation would require optimization for production on production scale. Aside from this reaction, the regioselectivity for desired isomers was excellent for all other C–H functionalization events. We expect the frequent suggestion by the software to convert C–H bonds into other functionalities is the result of the preference for low-cost starting materials in our heuristic, with C–H bonds in many instances being cheaper than other functionalities. The Friedel–Crafts acylation route described replaces six C–H bonds with new functionalities over seven reactions to convert **27** and **45** into **2**.

We next employed a different tactic. Most routes to **2** hinge on a Nenitzescu indole coupling^29^ between 1,4-benzoquinone and a β-aminocrotonic ester^21^. Indeed, the Nenitzescu reaction using known starting materials featured as a proposal in our query results when default search criteria were used, so the keyword “Nenitzescu” was used as an exclusion criterion. This heuristic did not employ a SMILES exclusion criterion, so starting material **52** was employed even though this chemical has been used in a prior synthesis of **2**. The results of this search led to yet another proposal to use a C–H bond as a feedstock, via a Bamberger rearrangement to install the C1 hydroxyl (Fig. 4, route D). SYNTHIA^TM^ proposed the use of 5-bromo-2-chloro-1-nitrobenzene as a starting material. In our hands, the requisite indole coupling on the chloride gave only traces of **39**, and we ultimately modified the starting material to use 2,5-dibromo-1-nitrobenzene (**49**) instead. This modification allowed the indole coupling to proceed, as discussed below, with the added benefit that **49** is cheaper than the corresponding chloride. In practice, **49** was reduced to the hydroxylamine, and treated with aqueous trifluoroacetic acid to affect the Bamberger rearrangement yielding **50**, which was methylated to arrive at **51**. Copper-catalyzed coupling to **53**, itself obtained through the union of **52** and **38**, produced **39** in 66% yield when **54** was used as a ligand. These conditions were the result of a rapid optimization campaign using high-throughput experimentation (see Supplementary Information). Subsequent alkylation of **39** with **40** produced **2**. With this latter route, convergency is maximized, so the longest-linear sequence is just five steps.

### One-step synthesis of bromhexine

**Fig. 5:**
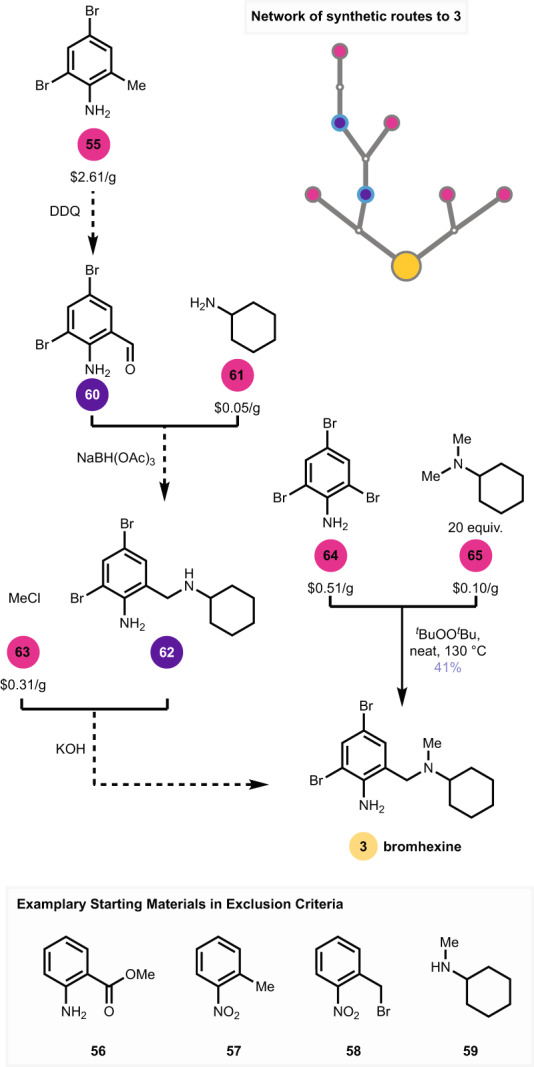
Predicted and realized routes to **3**. Dashed arrows are predicted, while the solid arrow is manually designed and experimentally validated, with the subsequent addition of the key reaction to the SYNTHIA^™^ database.

Finally, we looked at **3** (Fig. 5), a transmembrane protease, serine 2 (TMPRSS2) inhibitor that was being investigated in five clinical trials for COVID-19. A SYNTHIA^TM^ search provided new reaction sequences of comparable length to known routes, identifying **55** as a novel starting material^21^ by navigating around known starting materials **56**–**59** and others. The predicted route invoked a C–H oxidation of the benzylic methyl group, presumably to arrive at cheaper starting materials, which readied **60** for reductive amination with **61**. The proposed route completed the synthesis of **3** by *N*-methylation of **62** with **63**. We considered instead that **3** could be synthesized from 2,4,6-tribromoaniline (**64**, $0.51/g), which is used in the textile industry and readily available, with *N*,*N*-dimethylcyclohexylamine (**65**, $0.10/g), a commodity chemical used in oil refining, via the direct C–H functionalization recently reported by Shirakawa^34^. While this manually designed route does not serve as a test of the software’s capability per se, our motivation here was largely to do what we could as synthetic chemists to support the production of a potentially beneficial medicine during a pandemic. The key reaction was added to the SYNTHIA^TM^ database so it would appear as a general solution to subsequent searches, and indeed this route came up as a top hit in a subsequent search for **3**. To experimentally realize this one-step route, we found that **64** could be heated in excess **65** in the presence of *tert*-butylperoxide^34^ to produce **3** in 41% yield. Further optimization of reaction conditions—to improve yield, ease of purification of **3**, and address the hazard of using peroxide on large scale—would be needed for commercial production. Nonetheless, this strategic disconnection reduces **3**, in a single step, to starting materials that are considerably cheaper than those in commercial use.

In this work, we show disconnection of drugs into affordable reagents for 12 drugs through the merger of crowd-sourcing and retrosynthetic software. Four predicted routes to **2** and one route to **3**, manually designed but added to the software’s database for future use, were experimentally validated. Navigating the combinatorial explosion of routes towards twelve distinct synthetic targets to arrive at distinct and affordable starting materials was a data handling challenge that could only be navigated with automated retrosynthesis. Our work was performed over nine weeks in Spring 2020 against the backdrop of a developing pandemic. While full process development would require a longer timeline—for instance, reagents such as peroxides would likely be replaced for production on commercial scale—our results show that automated retrosynthetic predictions can rapidly highlight alternative starting material supply chains to pharmaceuticals.

## Methods

### Experimental methods

All reactions were conducted in the oven- or flame-dried glassware under an atmosphere of nitrogen unless stated otherwise. Reactions were set up in an MBraun LABmaster Pro Glove Box (H_2_O level <0.1 ppm, O_2_ level <0.1 ppm), or using standard Schlenk technique with a glass vacuum manifold connected to an inlet of dry nitrogen gas. Solvents (acetonitrile, tetrahydrofuran, and dichloromethane) were purified using an MBraun SPS solvent purification system, by purging with nitrogen and then passing the solvent through a column of activated alumina. Flash chromatography was performed on silica gel (230–400 Mesh, Grade 60) under a positive pressure of nitrogen. Thin-layer chromatography was performed on 25 µm TLC Silica gel 60 F_254_ glass plates purchased from Fisher Scientific (part number: S07876). Visualization was performed using ultraviolet light (254 nm), potassium permanganate (KMnO_4_) stain, or cerium ammonium molybdate stain. See Supplementary Information for additional details. Data used to produce the network visualizations in Fig. 2, which are available as an interactive visualizer at http://covidroutes.cernaklab.com/, are also available in the Supplementary Information. Reagent prices shown are the cheapest U.S. list price from a structure search on www.sigmaaldrich.com in May or June of 2020. For starting materials not available from this website, the price shown is the cheapest list price found in a SciFinder search of commercially available materials. Care must be taken in optimization studies based on peroxide activation of **64** and **65** to produce **3**, as we observed a violent reaction on a small scale between neat **65** and benzoylperoxide.

## Supplementary information

Supplementary Information

Peer review file

## Data Availability

Raw data and experimental procedures are available in the Supplementary Information. The collection of patents, publications, and SMILES strings of starting materials and intermediates used as exclusion criteria for searches in the commercial software SYNTHIA^TM^ are available as an interactive visualizer at http://covidroutes.cernaklab.com/.

## Code availability

No relevant code was used. Algorithmic searches were performed in the commercially available software SYNTHIA^TM^, the code for which is proprietary to Merck.
