# Author Correction: Reinforcing the supply chain of umifenovir and other antiviral drugs with retrosynthetic software

**DOI:** 10.1038/s41467-022-29041-w

**Published:** 2022-03-28

**Authors:** Yingfu Lin, Zirong Zhang, Babak Mahjour, Di Wang, Rui Zhang, Eunjae Shim, Andrew McGrath, Yuning Shen, Nadia Brugger, Rachel Turnbull, Sarah Trice, Shashi Jasty, Tim Cernak

**Affiliations:** 1 Department of Medicinal Chemistry, University of Michigan, Ann Arbor, MI 48109 USA; 2 Department of Chemistry, University of Michigan, Ann Arbor, MI 48109 USA; 3 MilliporeSigma, Burlington, MA 01803 USA; 4 Present Address: Entos, Inc., San Diego, CA 92037 USA

**Keywords:** Chemical synthesis, Cheminformatics

Correction to: *Nature Communications* 10.1038/s41467-021-27547-3, published online 16 December 2021.

The original version of this Article contained errors in the code availability statement, acknowledgements, and the competing interests statement.

In the code availability stated the software code is proprietary to ‘MilliporeSigma’ but should have read ‘Merck’.

In the acknowledgements section ‘Shane Krska and Melodie Christensen of Merck & Co., Inc.’ should have read ‘Shane Krska and Melodie Christensen of MSD’. Additionally, ‘this work was supported by MilliporeSigma’ has been corrected to ‘this work was supported by Merck’.

Finally, the competing interests statement stated ‘The Cernak Lab has received research funding or in-kind donations from MilliporeSigma, Relay Therapeutics, Janssen Therapeutics, SPT Labtech and Merck & Co., Inc.’ but should read as ‘The Cernak Lab has received research funding or in-kind donations from Merck, Relay Therapeutics, Janssen Therapeutics, SPT Labtech, and MSD.’

These have now been corrected in both the PDF and HTML versions of the Article.

