# Supplementary material for: Reinforcing the supply chain of umifenovir and other antiviral drugs with retrosynthetic software
Source: Nat Commun. 2021 Dec 16;12:7327. doi: 10.1038/s41467-021-27547-3 (PMC8677791; doi:10.1038/s41467-021-27547-3)
Supplement: Supplementary file 1 — Supplementary Information [file 41467_2021_27547_MOESM1_ESM.pdf]

Supplementary Information for

"Reinforcing the Supply Chain of Umifenovir and Other Antiviral Drugs with Retrosynthetic Software"

S-2 : 1. Supplementary Methods

S-2 : General Information

S-4 : Experimental

S-18 : 3. Supplementary Figures

S-18 : Spectra

S-46 : SYNTHIA^{TM} searches and predicted routes

S-80 : 2. Supplementary References

1. Supplementary Methods

General Information

All reactions were conducted in oven- or flame-dried glassware under an atmosphere of nitrogen unless stated otherwise. Reactions were set up in an MBraun LABmaster Pro Glove Box (H_2O level <0.1 ppm, O_2 level <0.1 ppm), or using standard Schlenk technique with a glass vacuum manifold connected to an inlet of dry nitrogen gas. Solvents (acetonitrile, tetrahydrofuran, dichloromethane) were purified using a MBraun SPS solvent purification system, by purging with nitrogen, and then passing the solvent through a column of activated alumina. *t*BuOH, 1,1,1,3,3,3-hexafluoro-2-propanol (HFIP) were used as received. Methanol, 1,4-dioxane, dimethyl sulfoxide (DMSO), *N,N*-dimethylformamide (DMF) were purchased as the anhydrous solvents and used as received. Reagents [4'-aminoacetophenone, ethyl acetoacetate, $InBr_3$, $MgSO_4$, $Pd(OAc)_2$, $Cu(OAc)_2$, K_2CO_3, NaH, MeI, trimethylsilyl trifluoromethanesulfonate (TMSOTf), *N*,*N*-diisopropylethylamine (DIPEA), *N*-chlorosuccinimide (NCS), 3-chloroperbenzoic acid (*m*CPBA), Na_2HPO_4, bromine, KOH, thiophenol, 4-ethylaniline, aniline, sodium bis(2-hydroxy-2-methylbutyrato)oxochromate(V), chloroacetyl chloride, $AlCl_3$, hydrazine monohydrate, trifluoroacetic acid (TFA), palladium on activated carbon, paraformaldehyde, $NaBH_4$, CuI, Cs_2CO_3, triethylamine (Et_3N), 2,4,6-tribromoaniline, *N*,*N*-dimethylcyclohexanamine, di-*tert*-butyl peroxide, 2,5-dibromonitrobenzene, ethyl 4-chloroacetoacetate, *N*,*N*,*N'*,*N'*-tetramethyldiaminomethane] were purchased from Sigma Aldrich, Alfa Aesar, Oakwood Chemical, or TCI Chemical. All chemicals were used as received. Glass 2 dram vials (ChemGlass #CG-4912-02) were used as reaction vessels, fitted with a screw-cap with a Teflon-coated silicone septa (CG-4910-02), and magnetic stir bars (Fisher Scientific #14-513-93 or #14-513-65). High temperature reactions were performed in crimp cap vials (Biotage #351521).

Proton nuclear magnetic resonance spectra (^1H NMR) were recorded on a Varian MR-500 MHz or Varian MR-400 MHz spectrometer and chemical shifts are reported in parts per million (ppm) using the solvent residual peak as an internal standard ($CDCl_3$ at 7.26 ppm, $(CD_3)_2SO$ at 2.50 ppm, CD_3CN at 1.94 ppm). Data are reported using the abbreviations: app = apparent, s = singlet, d = doublet, t = triplet, q = quartet, m = multiplet, comp = complex, br = broad. Coupling constant(s) are reported in Hz. Proton-decoupled carbon nuclear magnetic resonance spectra (^{13}C NMR) spectra were recorded on a Varian MR-500 MHz or Varian MR-400 MHz spectrometer and chemical shifts are reported in ppm using the solvent as an internal standard ($CDCl_3$ at 77.16 ppm, $(CD_3)_2SO$ at 39.52 ppm, CD_3CN at 1.32 ppm). High resolution mass spectrometry data (HRMS) was obtained on a Micromass AutoSpec Ultima Magnetic Sector instrument. Reaction analysis was typically performed by thin-layer chromatography on silica gel, or using a Waters I-class ACQUITY UPLC-MS (Waters Corporation, Milford, MA, USA) equipped with in-line photodiode array detector (PDA) and QDa mass detector (ESI positive ionization mode). 0.1 µL sample injections were taken from acetonitrile solutions of reaction mixtures or products (~1 mg/mL). A partial loop injection mode was used with the needle placement at 1.0 mm from bottom of the wells and a 0.2 µL

air gap at pre-aspiration and post-aspiration. Column used: Waters Cortecs UPLC C18+ column, 2.1mm × 50 mm with (Waters #186007114) with Waters Cortecs UPLC C18+ VanGuard Pre-column 2.1mm × 5 mm (Waters #186007125), Mobile Phase A: 0.1 % formic acid in Optima LC/MS-grade water, Mobile Phase B: 0.1% formic acid in Optima LC/MS-grade MeCN. Flow rate: 1 mL/min. Column temperature: 45 °C. The PDA sampling rate was 20 points/sec. The QDa detector monitored m/z 150-750 with a scan time of 0.06 seconds and a cone voltage of 30 V. The PDA detector range was between 210 nm – 400 nm with a resolution of 1.2 nm. 1 minute and 2 minute methods were used. The method gradients are below: 0 min: 0.8 mL/min, 95% 0.1% formic acid in water/5% 0.1% formic acid in acetonitrile; 1.5 min : 0.8 mL/min, 0.1% 0.1% formic acid in water/99.9% 0.1% formic acid in acetonitrile; 1.91 min : 0.8 mL/min, 95% 0.1% formic acid in water/5% 0.1% formic acid in acetonitrile.

Flash chromatography was performed on silica gel (230 – 400 Mesh, Grade 60) under a positive pressure of Nitrogen. Thin Layer Chromatography was performed on 25 µm TLC Silica gel 60 F_{254} glass plates purchased from Fisher Scientific (part number: S07876). Visualization was performed using ultraviolet light (254 nm), potassium permanganate ($KMnO_4$) stain, or Cerium Ammonium Molybdate (CAM) stain.

Experimental

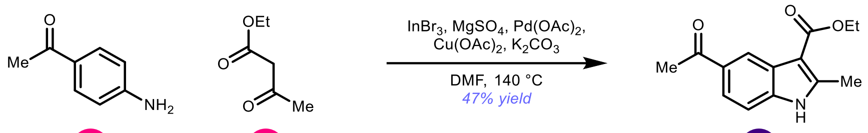

26 27 32

ethyl 5-acetyl-2-methyl-1*H*-indole-3-carboxylate (32).

Following a modified literature procedure (1), to an oven-dried 2-dram vial with a Teflon-coated stir bar was added 4’-aminoacetophenone (**26**) (1.081 g, 8.00 mmol, 1.00 equiv), ethyl acetoacetate (**27**) (1.518 mL, 12.00 mmol, 1.50 equiv) and $MgSO_4$ (0.963 g, 8.00 mmol, 1 equiv). Then the vial was moved into the glovebox, and $InBr_3$ (28.4 mg, 0.08 mmol, 1 mol%) was added. Then the vial was capped and moved out of the glove box. The reaction mixture was stirred at 80 °C for 2 h. The mixture was transferred with a syringe into a 250 mL round bottom flask containing $Pd(OAc)_2$ (89.8 mg, 0.40 mmol, 5 mol%), $Cu(OAc)_2$ (4.359 g, 24.00 mmol, 3.0 equiv), K_2CO_3 (3.317 g, 24.00 mmol, 3.0 equiv), $MgSO_4$ (0.963 g, 8.00 mmol, 1 equiv) and DMF (70 mL), and the product residues were rinsed into the bigger flask with DMF (2 × 5 mL). The reaction mixture was treated at 140 °C under N_2 atmosphere for 1 h before it was cooled to room temperature, diluted with EtOAc (100 mL) and filtered through a short pad of silica and sea sand. The red-brown solid was washed with EtOAc (2 × 80 mL) and the combined filtrates were concentrated *in vacuo* to yield a crude product, which was purified by flash column chromatography (silica gel, 5% EtOAc/CH_2Cl_2) to yield the product (0.915 g, 3.73 mmol, 47%)

^{1}H NMR (500 MHz, $(CD_3)_2SO$) δ 12.12 (s, 1H), 8.59 (d, *J* = 1.7 Hz, 1H), 7.76 (dd, *J* = 8.5, 1.4 Hz, 1H), 7.43 (d, *J* = 8.5 Hz, 1H), 4.30 (q, *J* = 7.1 Hz, 2H), 2.67 (s, 3H), 2.60 (s, 3H), 1.37 (t, *J* = 7.1 Hz, 3H). ^{13}C NMR (125 MHz, $(CD_3)_2SO$) δ 197.41, 164.68, 146.43, 137.50, 130.43, 126.42, 121.97, 121.91, 111.13, 103.96, 59.04, 26.56, 14.37, 13.72. HRMS (ESI): calculated $C_{14}H_{16}NO_3$ $[M+H]^+$: 246.1125, found: 246.1121.

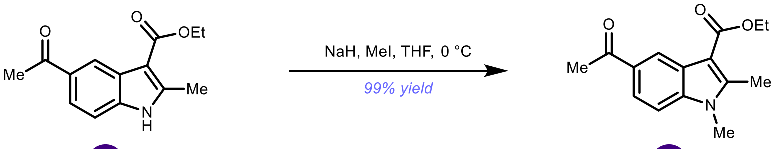

32 33

ethyl 5-acetyl-1,2-dimethyl-1*H*-indole-3-carboxylate (33):

To a solution of compound **32** (0.217 g, 0.885 mmol, 1.00 equiv) in anhydrous THF (8.8 mL) was added NaH (60 % dispersion in mineral oil, 42.5 mg, 1.062 mmol, 1.2 equiv) at 0 °C. After 30 min, iodomethane (66.1 µL, 1.062 mmol, 1.2 equiv) was added dropwise at 0 °C. The mixture was stirred at the same temperature for 30 min before it was quenched with water (0.2 mL). After removal of the solvents on a rotary evaporator, the residue was purified by flash column chromatography (silica gel, 2% EtOAc/CH_2Cl_2) to yield the product (0.228 g, 0.879 mmol, 99%).

^1H NMR (500 MHz, $CDCl_3$) δ 8.79 (d, *J* = 1.8 Hz, 1H), 7.92 (dd, *J* = 8.7, 1.7 Hz, 1H), 7.33 (d, *J* = 8.6 Hz, 1H), 4.43 (q, *J* = 7.1 Hz, 2H), 3.74 (s, 3H), 2.79 (s, 3H), 2.69 (s, 3H), 1.48 (t, *J* = 7.1 Hz, 3H). ^{13}C NMR (125 MHz, $CDCl_3$) δ 198.16, 165.32, 146.54, 138.72, 131.00, 125.88, 123.29, 121.78, 108.87, 104.92, 59.52, 29.59, 26.45, 14.44, 11.74. HRMS (ESI): calculated $C_{15}H_{18}NO_3$ $[M+H]^+$: 260.1281, found: 260.1278.

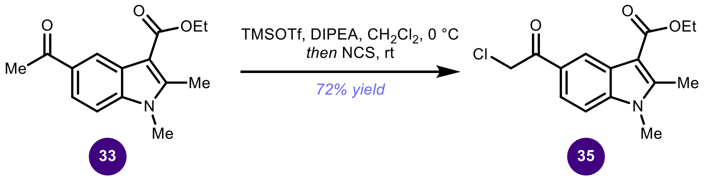

33

35

ethyl 5-(2-chloroacetyl)-1,2-dimethyl-1*H*-indole-3-carboxylate (35):

Following a modified literature procedure (2), to a solution of compound **33** (25.9 mg, 0.10 mmol, 1.00 equiv.) in dichloromethane (1 mL) was added 4Å molecular sieves (10.0 mg) and *N*,*N*-diisopropylethylamine (52.3 µL, 0.30 mmol, 3.00 equiv) at room temperature. The mixture was cooled to 0 °C, then TMSOTf (54.2 µL, 0.30 mmol, 3.00 equiv) was added and stirred for 2 h. The reaction mixture was allowed to warm to room temperature and *N*-chlorosuccinimide (NCS) (20.0 mg, 0.15 mmol, 1.50 equiv) was added. The reaction was stirred for an additional 2 h, then quenched with saturated aqueous $NaHCO_3$ at room temperature. The crude mixture was extracted with EtOAc (2 × 3 mL). The organic layers were combined, washed with brine, and dried over anhydrous sodium sulfate. The organic layer was then concentrated under reduced pressure and purified by flash column chromatography (silica gel, 2% – 5% EtOAc/CH_2Cl_2) to afford the product (21.1 mg, 0.072 mmol, 72%).

^1H NMR (500 MHz, $CDCl_3$) δ 8.78 (d, *J* = 1.7 Hz, 1H), 7.92 (dd, *J* = 8.7, 1.8 Hz, 1H), 7.36 (d, *J* = 8.7 Hz, 1H), 4.83 (s, 2H), 4.43 (q, *J* = 7.1 Hz, 2H), 3.75 (s, 3H), 2.80 (s, 3H), 1.49 (t, *J* = 7.1 Hz, 3H). ^{13}C NMR (125 MHz, $CDCl_3$) δ 191.14, 165.43,

147.18, 139.38, 128.18, 126.22, 123.51, 122.51, 109.60, 105.44, 59.88, 46.36, 29.99, 14.62, 12.06. HRMS (ESI): calculated $C_{15}H_{17}ClNO_3$ [M+H]$^+$: ^{35}Cl 294.0891, found: 294.0895.

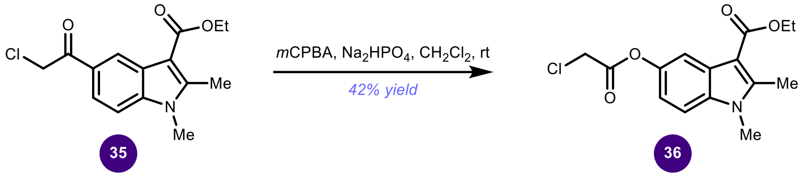

ethyl 5-(2-chloroacetoxy)-1,2-dimethyl-1*H*-indole-3-carboxylate (36):

Following a modified literature procedure (3), *m*CPBA (70%, 37.0 mg, 0.15 mmol, 3.00 equiv) was added to a mixture of compound **35** (14.7 mg, 0.05 mmol, 1.00 equiv) and Na_2HPO_4 (21.3 mg, 0.15 mmol, 3.00 equiv) in dichloromethane (0.5 mL) at room temperature. The reaction mixture was stirred at the same temperature for 1 h before quenched with saturated aqueous sodium thiosulfate (1 mL) and saturated aqueous sodium bicarbonate (1 mL). The crude mixture was extracted with EtOAc (2 × 3 mL). The organic layers were combined, washed with brine, and dried over anhydrous sodium sulfate. The organic layer was then concentrated under reduced pressure and purified by flash column chromatography (silica gel, 17% EtOAc/heaxnes) to afford the product (6.5 mg, 0.021 mmol, 42%).

^{1}H NMR (500 MHz, $CDCl_3$) δ 7.84 (d, *J* = 2.4 Hz, 1H), 7.27 (d, *J* = 9.0 Hz, 1H), 6.99 (dd, *J* = 8.7, 2.3 Hz, 1H), 4.39 (q, *J* = 7.1 Hz, 2H), 4.35 (s, 2H), 3.70 (s, 3H), 2.77 (s, 3H), 1.43 (t, *J* = 7.1 Hz, 3H). ^{13}C NMR (125 MHz, $CDCl_3$) δ 166.76, 165.80, 146.69, 145.62, 134.60, 127.12, 115.50, 113.56, 109.69, 104.35, 59.62, 41.17, 29.86, 14.74, 12.05. HRMS (ESI): calculated $C_{15}H_{17}ClNO_4$ [M+H]$^+$: ^{35}Cl 310.0841, found: 310.0778.

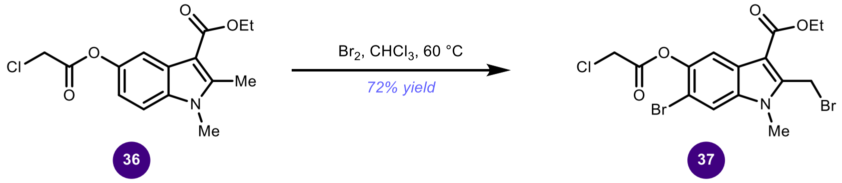

ethyl 6-bromo-2-(bromomethyl)-5-(2-chloroacetoxy)-1-methyl-1*H*-indole-3-carboxylate (37):

Following a modified literature procedure (4), to a solution of compound **36** (12.0 mg, 0.039 mmol, 1.00 equiv) in chloroform (0.4 mL) was added bromine (4.0 µL, 0.078 mmol, 2.00 equiv) dropwise at room temperature. After addition, the mixture was treated at 60 °C for 2 h before quenched with saturated aqueous sodium thiosulfate (1 mL) and saturated aqueous sodium bicarbonate (1 mL). The crude mixture was extracted with EtOAc (2 × 3 mL). The organic layers were combined, washed with brine, and dried over anhydrous sodium sulfate. The organic layer was then concentrated under reduced pressure and purified by flash column chromatography (silica gel, 15% EtOAc/heaxnes) to afford the product (13.0 mg, 0.028 mmol, 72%).

^{1}H NMR (500 MHz, $CDCl_3$) δ 7.93 (s, 1H), 7.61 (s, 1H), 5.12 (s, 2H), 4.48 – 4.38 (m, 4H), 3.79 (s, 3H), 1.45 (t, *J* = 7.1 Hz, 3H). ^{13}C NMR (125 MHz, $CDCl_3$) δ 165.94, 164.43, 143.06, 142.98, 135.96, 125.86, 116.34, 114.28, 111.53, 106.09, 60.48, 40.94, 30.32, 20.57, 14.65. HRMS (ESI): calculated $C_{15}H_{14}Br_2ClNO_4Na$ $[M+Na]^+$: $^{35}Cl/^{79}Br/^{79}Br$ 487.8870, found: 487.8868.

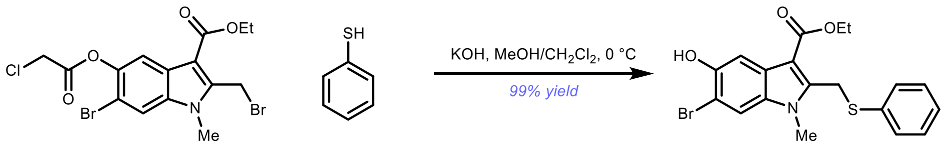

37 38 39

ethyl 6-bromo-5-hydroxy-1-methyl-2-((phenylthio)methyl)-1*H*-indole-3-carboxylate (39):

Following a modified literature procedure (4) to a solution of potassium hydroxide (85%, 5.1 mg, 0.077 mmol, 3.00 equiv) in methanol (0.1 mL) was added thiophenol (5.3 µL, 0.051 mmol, 2.00 equiv) and the mixture was stirred at room temperature for 15 min before it was cooled in an ice bath. To the above mixture was added a solution of compound **37** (12.0 mg, 0.026 mmol, 1.00 equiv) in dichloromethane (0.25 mL) at 0 °C. The resulting mixture was stirred at the same temperature for 1 h before it was quenched with acetic acid (8 µL). The solvent was removed under reduced pressure and the residue was purified by flash column chromatography (silica gel, 20% EtOAc/hexanes) to yield the product (10.7 mg, 0.026 mmol, 99%).

The characterization data matched spectral values from our synthesis of **39** (Route D).

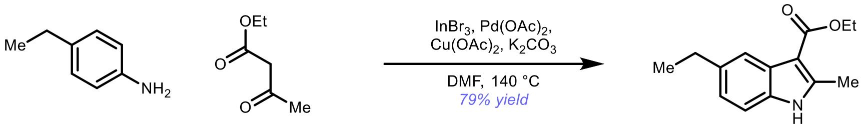

41 27 42

ethyl 5-ethyl-2-methyl-1*H*-indole-3-carboxylate (42):

Following a modified literature procedure (1) to an oven-dried 2-dram vial with a Teflon-coated stir bar was added 4-ethylaniline (**41**) (0.621 mL, 5.00 mmol, 1.00 equiv), and ethyl acetoacetate (0.696 mL, 5.50 mmol, 1.10 equiv). Then the vial was moved into the glovebox, and $InBr_3$ (17.7 mg, 0.05 mmol, 1 mol%) was added. Then the vial was capped and moved out of the glove box. The reaction mixture was stirred at room temperature for 30 min. The mixture was transferred with a syringe into a 250 mL round bottom flask containing $Pd(OAc)_2$ (56.1 mg, 0.25 mmol, 5 mol%), $Cu(OAc)_2$ (2.724 g, 15.00 mmol, 3.0 equiv), K_2CO_3 (2.073 g, 15.00 mmol, 3.0 equiv), and DMF (40 mL), and the product residues were rinsed into the bigger flask with DMF (2 × 5 mL). The reaction mixture was treated at 140 °C under N_2 atmosphere for 30 min before it was cooled to room temperature, diluted with EtOAc (100 mL) and filtered through a short pad of silica and sea sand. The red-brown solid was washed with EtOAc (2 × 80 mL) and the combined filtrates were concentrated on a rotary evaporator and in high vacuum to yield a crude product, which was purified by flash column chromatography (silica gel, 20% EtOAc/hexanes) to yield the product (0.916 g, 3.96 mmol, 79%)

^{1}H NMR (500 MHz, $CDCl_3$) δ 8.25 (br s, 1H), 7.94 (s, 1H), 7.21 (d, J = 8.2 Hz, 1H), 7.05 (d, J = 8.2 Hz, 1H), 4.40 (q, J = 7.1 Hz, 2H), 2.76 (q, J = 7.6 Hz, 2H), 2.72 (s, 3H), 1.45 (t, J = 7.1 Hz, 3H), 1.29 (t, J = 7.6 Hz, 3H). ^{13}C NMR (125 MHz, $CDCl_3$) δ 166.45, 144.10, 137.91, 133.10, 127.62, 122.83, 119.98, 110.41, 104.34, 59.59, 29.31, 16.53, 14.74, 14.43. HRMS (ESI): calculated $C_{14}H_{17}NNaO_2$ $[M+Na]^+$: 254.1151, found: 254.1148.

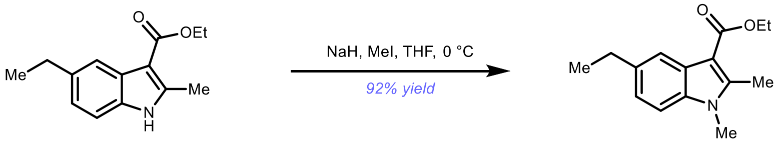

42 43

ethyl 5-ethyl-1,2-dimethyl-1*H*-indole-3-carboxylate (43):

To a solution of compound **42** (0.600 g, 2.59 mmol, 1.00 equiv) in anhydrous THF

(26 mL) was added NaH (60 % dispersion in mineral oil, 125 mg, 3.11 mmol, 1.2 equiv) at 0 °C. After 30 min, iodomethane (194 µL, 3.11 mmol, 1.2 equiv) was added dropwise at 0 °C. The mixture was stirred at the same temperature for 30 min before it was quenched with water (0.5 mL). After removal of the solvents on a rotary evaporator, the residue was purified by flash column chromatography (silica gel, 15% EtOAc/hexanes) to yield the product (0.583 g, 2.38 mmol, 92%).

^{1}H NMR (500 MHz, $CDCl_3$) δ 7.96 (s, 1H), 7.20 (d, *J* = 8.3 Hz, 1H), 7.09 (dd, *J* = 8.3, 1.8 Hz, 1H), 4.41 (q, *J* = 7.2 Hz, 2H), 3.67 (s, 3H), 2.83 – 2.69 (m, 5H), 1.46 (t, *J* = 7.1 Hz, 3H), 1.30 (t, *J* = 7.6 Hz, 3H). ^{13}C NMR (125 MHz, $CDCl_3$) δ 166.42, 145.26, 137.89, 135.22, 126.99, 122.49, 120.23, 108.92, 103.76, 59.43, 29.73, 29.30, 16.58, 14.78, 12.05. HRMS (ESI): calculated $C_{15}H_{20}NO_2$ $[M+H]^+$: 246.1489, found: 246.1481.

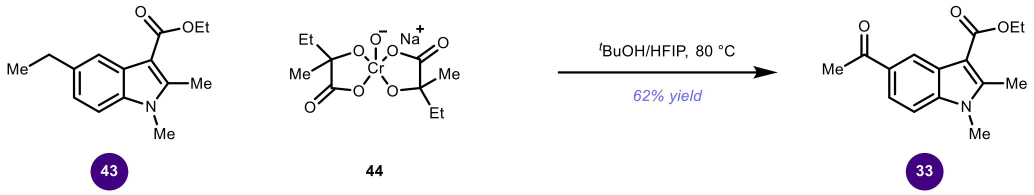

ethyl 5-acetyl-1,2-dimethyl-1*H*-indole-3-carboxylate (33):

Following a modified literature procedure (5), compound **43** (24.5 mg, 0.10 mmol, 1.00 equiv), and sodium bis(2-hydroxy-2-methylbutyrato)oxochromate(V) (compound **44**, 194 mg, 0.60 mmol, 6.00 equiv) were placed in a 2-dram vial with a stir bar. ^{t}BuOH (0.10 mL) and HFIP (0.90 mL) were added, and the mixture was then stirred at 80 °C for 5 h, open to air. On complete consumption of compound **43**, silica gel (582 mg, 300 wt% to **44**) was added. After removal of solvent under reduced pressure, the resulting residue was purified by flash chromatography (silica gel, 25% – 30% EtOAc/hexanes) to afford the product (16.0 mg, 0.062 mmol, 62%)

The characterization data matched spectral values from our earlier synthesis of **33** (Route A).

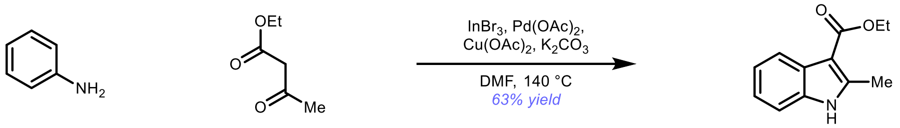

45 27 46

ethyl 2-methyl-1*H*-indole-3-carboxylate (46):

Following a modified literature procedure (1), to an oven-dried 2-dram vial with a Teflon-coated stir bar was added aniline (**45**) (0.729 mL, 8.00 mmol, 1.00 equiv), and ethyl acetoacetate (1.113 mL, 8.80 mmol, 1.10 equiv). Then the vial was moved into the glovebox, and $InBr_3$ (28.4 mg, 0.08 mmol, 1 mol%) was added. Then the vial was capped and moved out of the glove box. The reaction mixture was stirred at room temperature for 30 min. The mixture was transferred with a syringe into a 250 mL round bottom flask containing $Pd(OAc)_2$ (89.8 mg, 0.40 mmol, 5 mol%), $Cu(OAc)_2$ (4.359 g, 24.00 mmol, 3.0 equiv), K_2CO_3 (3.317 g, 24.00 mmol, 3.0 equiv), and DMF (70 mL), and the residues were rinsed into the bigger flask with DMF (2 × 5 mL). The reaction mixture was stirred at 140 °C under N_2 atmosphere for 30 min before it was cooled to room temperature, diluted with EtOAc (100 mL) and filtered through a short pad of silica and sea sand. The red-brown solid was washed with EtOAc (2 × 80 mL) and the combined filtrates were concentrated *in vacuo* to yield a crude product, which was purified by flash column chromatography (silica gel, 20% EtOAc/hexanes) to yield the product (1.015 g, 5.00 mmol, 63%).

^{1}H NMR (500 MHz, $CDCl_3$) δ 8.44 (br s, 1H), 8.11 (d, J = 7.3 Hz, 1H), 7.30 (dd, J = 6.9, 1.3 Hz, 1H), 7.24 – 7.16 (m, 2H), 4.41 (q, J = 7.2 Hz, 2H), 2.74 (s, 3H), 1.45 (t, J = 7.1 Hz, 3H). ^{13}C NMR (125 MHz, $CDCl_3$) δ 166.29, 144.06, 134.62, 127.33, 122.46, 121.81, 121.46, 110.61, 104.80, 59.65, 14.74, 14.36. HRMS (ESI): calculated $C_{12}H_{13}NNaO_2$ $[M+Na]^+$: 226.0838, found: 226.1582.

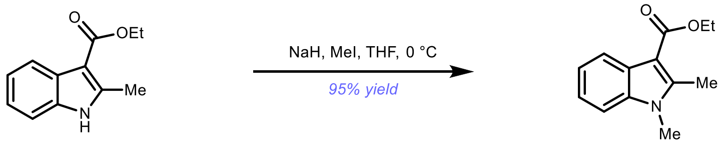

46 47

ethyl 1,2-dimethyl-1*H*-indole-3-carboxylate (47):

To a solution of compound **46** (1.003 g, 4.935 mmol, 1.00 equiv) in anhydrous THF (49 mL) was added NaH (60 % dispersion in mineral oil, 237 mg, 5.922 mmol, 1.2 equiv) at 0 °C. After 30 min, iodomethane (369 µL, 5.922 mmol, 1.2 equiv) was

added dropwise at 0 °C. The mixture was stirred at the same temperature for 30 min before it was quenched with water (1.0 mL). After removal of the solvents on a rotary evaporator, the residue was purified by flash column chromatography (silica gel, 15% EtOAc/hexanes) to yield the product (1.016 g, 4.676 mmol, 95%).

^{1}H NMR (500 MHz, $CDCl_3$) δ 8.16 – 8.11 (m, 1H), 7.30 – 7.26 (m, 1H), 7.25 – 7.20 (m, 2H), 4.41 (q, J = 7.1 Hz, 2H), 3.66 (s, 3H), 2.76 (s, 3H), 1.46 (t, J = 7.1 Hz, 3H). ^{13}C NMR (126 MHz, $CDCl_3$) δ 166.31, 145.38, 136.60, 126.70, 122.05, 121.70, 121.55, 109.12, 104.03, 59.47, 29.64, 14.74, 11.94. HRMS (ESI): calculated $C_{13}H_{15}NNaO_2$ [M+Na]$^+$: 240.0995, found: 240.0992.

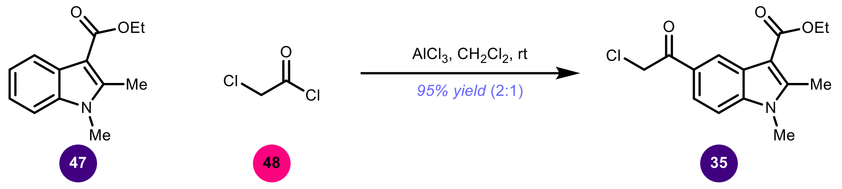

ethyl 5-(2-chloroacetyl)-1,2-dimethyl-1*H*-indole-3-carboxylate (35):

Following a modified literature procedure (3), to a suspension of anhydrous $AlCl_3$ (1.600 g, 12.00 mmol, 4.00 equiv) in anhydrous dichloromethane (15 mL) at 0 °C was added chloroacetyl chloride **48** (1.004 mL, 12.60 mmol, 4.2 equiv) dropwise. The mixture was stirred at 0 °C for 20 min before warmed up to room temperature. To this solution was added compound **47** (0.652 g, 3.00 mmol, 1.00 equiv). After the reaction mixture was stirred at room temperature for 3 h, it was poured into ice-cold water and extracted with EtOAc (3 × 40 mL). The organic layers were combined, washed with brine, and dried over anhydrous sodium sulfate. The organic layer was then concentrated under reduced pressure and purified by flash column chromatography (silica gel, 25% EtOAc/hexanes) to afford a mixture of two regioisomers (2:1, 838 mg, 2.854 mmol, 95%). The major regioisomer is compound **35**.

The characterization data matched spectral values from our earlier synthesis of **35** (Route A).

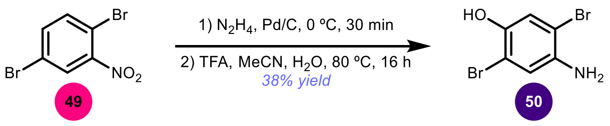

4-amino-2,5-dibromophenol (50):

To a 50 mL round bottom flask, **49** (2.81 g, 10.0 mmol, 1.00 equiv) and palladium on activated carbon (53.4 mg, 0.500 mmol, 0.0500 equiv) were added in tetrahydrofuran (25.0 mL) and cooled to 0 ºC. Hydrazine monohydrate (0.970 mL, 20 mmol, 2.00 equiv) was added dropwise. The reaction was stirred at 0 °C for 30 minutes and warmed to 25 ºC. The mixture was filtered through a pad of Celite and concentrated under reduced pressure. The crude mixture was used for next step without further purification. (6)

To a 100 mL round bottom flask, *N*-(2,5-dibromophenyl)hydroxylamine from last step and trifluoroacetic acid (3.80 mL, 50.0 mmol, 5.00 equiv) were dissolved in acetonitrile (10.0 mL) and water (40.0 mL) under nitrogen atmosphere. The reaction was stirred and heated at 80 °C for 16 hours. The reaction mixture was neutralized with saturated aqueous sodium bicarbonate solution (pH = 8), extracted with ethyl acetate (3 × 40 mL, dried over magnesium sulfate and concentrated under reduced pressure. The residue was purified with silica gel column chromatography (15% ethyl acetate in hexanes) to afford the product (1.00 g, 38%). (7)

^1H NMR (500 MHz, CD_3CN) δ 6.99 (s, 1H), 6.94 (s, 1H), 6.68 (s, 1H), 4.14 (s, 2H). ^{13}C NMR (125 MHz, CD_3CN) δ 146.26, 140.72, 120.60, 119.66, 110.55, 108.45. HRMS (ESI): calculated $C_6H_6Br_2NO$ $[M+H]^+$: $^{79}Br/^{79}Br$ 265.8811, found: 265.8806.

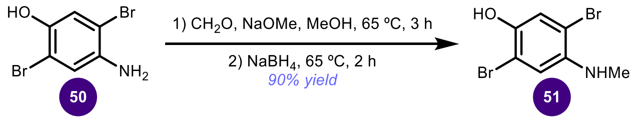

6-bromo-5-hydroxy-1-methyl-2-((phenylthio)methyl)-1*H*-indole-3-carboxylate (51):

To a 10 mL round bottom flask, **50** (187 mg, 0.700 mmol, 1 equiv), sodium methoxide (270 mg, 5.00 mmol, 7.14 equiv) and paraformaldehyde (70.1 mg, 2.5 mmol, 3.57 equiv) were dissolved in methanol (4.00 mL) under nitrogen atmosphere. The reaction mixture was stirred and heated at 65 °C for 3 hours before cooled to 25 ºC. After sodium borohydride (94.6 mg, 2.5 mmol, 3.57 equiv) was added, reaction was heated to 65 ºC and stirred for 2 hours. The reaction mixture was neutralized with saturated aqueous ammonium chloride solution (pH = 7), extracted with ethyl acetate (3 × 5 mL), dried over magnesium sulfate and concentrated under reduced pressure. The residue was purified with silica gel column chromatography (10% ethyl acetate in hexanes) to afford the product (176.4 mg, 90%). (8)

^1H NMR (500 MHz, CD_3CN) δ 7.08 (s, 1H), 6.76 (s, 1H), 6.62 (s, 1H), 4.27 (s, 1H), 2.75 (s, 3H). ^{13}C NMR (125 MHz, CD_3CN) δ 145.35, 142.48, 121.17, 115.02, 110.81, 108.89, 31.20. HRMS (ESI): calculated $C_7H_8Br_2NO$ $[M+H]^+$: $^{79}Br/^{79}Br$ 279.8967, found: 279.8963.

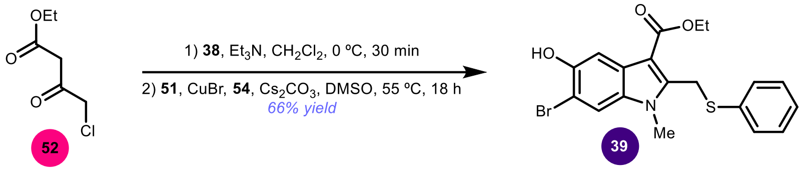

6-bromo-5-hydroxy-1-methyl-2-((phenylthio)methyl)-1*H*-indole-3-carboxylate (39):

To a 2-dram vial, **52** (94.6 µL, 0.700 mmol, 1.00 equiv) and thiophenol (71.4 µL, 0.700 mmol, 1.00 equiv) were dissolved in dichloromethane (4.00 mL). The reaction mixture was cooled to 0 ºC and triethylamine (105 µL, 0.750 mmol, 1.07 equiv) was added dropwise. The reaction was stirred for 30 minutes before warmed to 25 ºC. The reaction mixture was extracted sequentially with saturated aqueous sodium bicarbonate solution (4 mL), hydrochloric acid (1 M, 4 mL) and saturated sodium chloride solution (4 mL), dried over magnesium sulfate and concentrated under reduced pressure. The crude mixture was used for next step without further purification. (9)

To a 2-dram vial, **51** (28.1 mg, 0.100 mmol, 1.00 equiv), ethyl 3-oxo-4-(phenylthio)butanoate from last step, copper (I) bromide (2.9 mg, 0.020 mmol, 0.2 equiv), 2-(1H-tetrazol-1-yl)acetic acid (5.1 mg, 0.040 mmol, 0.4 equiv) and cesium carbonate (97.7 mg, 0.300 mmol, 3.00 equiv) were dissolved in dimethyl sulfoxide (1.00 mL) under nitrogen atmosphere. The reaction mixture was stirred at 55 °C for 18 hours. The reaction mixture was neutralized with saturated aqueous ammonium chloride solution (1 mL), extracted with ethyl acetate (3 × 2 mL), dried over magnesium sulfate and concentrated under reduced pressure. The residue was purified with silica gel column chromatography (10% ethyl acetate in hexanes to afford 6-bromo-5-hydroxy-1-methyl-2-((phenylthio)methyl)-1H-indole-3-carboxylate (27.7 mg, 66%). (10)

^1H NMR (500 MHz, $(CD_3)_2SO$) δ 9.81 (s, 1H), 7.73 (s, 1H), 7.56 (s, 1H), 7.36 – 7.33 (m, 2H), 7.31 – 7.24 (m, 3H), 4.78 (s, 2H), 4.18 (q, *J* = 7.1 Hz, 2H), 3.66 (s, 3H), 1.29 (t, *J* = 7.1 Hz, 3H). ^{13}C NMR (125 MHz, $(CD_3)_2SO$) δ 164.18, 149.27, 143.36, 134.22, 131.46, 131.09, 128.96, 127.24, 125.96, 114.22, 106.40, 106.28, 103.06, 59.11, 30.16, 28.26, 14.29. HRMS (ESI): calculated $C_{19}H_{18}BrNNaO_3S$ $[M+Na]^+$: ^{79}Br 442.0083, found: 442.0081.

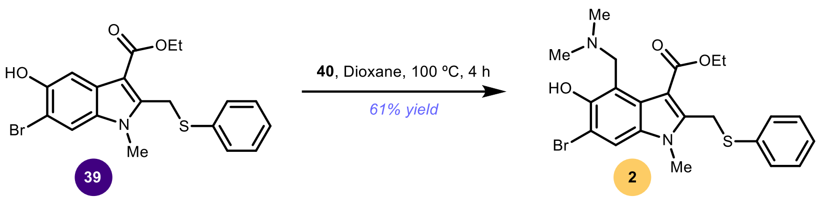

ethyl 6-bromo-4-((dimethylamino)methyl)-5-hydroxy-1-methyl-2-((phenylthio)methyl)-1*H*-indole-3-carboxylate (2):

To a 1-dram vial, **39** (23.8 mg, 0.0566 mmol, 1 equiv) and **40** (20 µL, 0.150 mmol, 2.65 equiv) were dissolved in 1,4-dioxane (0.30 mL). The reaction was stirred and heated to 100 ºC for 4 h before removing the solvent under reduced pressure. The residue was purified with silica gel column chromatography (5% methanol in dichloromethane) to afford the product (14.6 mg, 61%). (11)

^{1}H NMR (500 MHz, $(CD_3)_2SO$) δ 7.71 (s, 1H), 7.37 – 7.23 (m, 5H), 4.63 (s, 2H), 4.14 (q, *J* = 7.1 Hz, 2H), 4.03 (s, 2H), 3.66 (s, 3H), 2.27 (s, 6H), 1.23 (t, *J* = 7.1 Hz, 3H). ^{13}C NMR (125 MHz, $(CD_3)_2SO$) δ 64.80, 150.41, 141.67, 134.37, 131.31, 131.01, 129.06, 127.26, 123.45, 113.08, 112.58, 107.37, 105.23, 59.86, 59.04, 43.54, 30.17, 28.75, 14.04. HRMS (ESI): calculated $C_{22}H_{26}BrN_2O_3S$ $[M+H]^+$: ^{79}Br 477.0842, found: 477.0844.

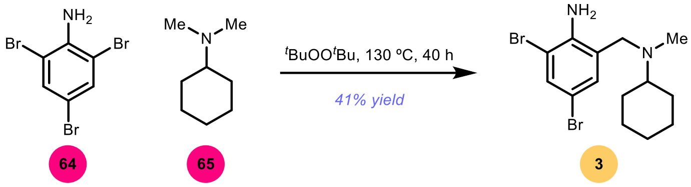

2,4-dibromo-6-((cyclohexyl(methyl)amino)methyl)aniline (3):

To a 2-dram vial, 2,4,6-tribromoaniline (98.9 mg, 0.300 mmol, 1 equiv) and 2-(tert-butylperoxy)-2-methylpropane (110 µL, 0.600 mmol, 2 equiv) were dissolved in *N*,*N*-dimethylcyclohexanamine (0.90 mL, 6.0 mmol, 20 equiv) under nitrogen atmosphere. The reaction was stirred and heated at 130 °C for 40 hours. The mixture was diluted with ethyl acetate (3 mL), dried over magnesium sulfate and concentrated under reduced pressure. The residue was purified with silica gel column chromatography (5% ethyl acetate in hexanes) to afford the product (45.9 mg, 41%). (12)

^{1}H NMR (500 MHz, $CDCl_3$) δ 7.46 (d, *J* = 2.3 Hz, 1H), 7.04 (d, *J* = 2.3 Hz, 1H), 5.48 (s, 2H), 3.59 (s, 2H), 2.41 (tt, *J* = 11.3, 3.1 Hz, 1H), 2.11 (s, 3H), 1.83 – 1.77 (m, 4H), 1.68 – 1.59 (m, 1H), 1.36 – 1.17 (m, 4H), 1.09 (qt, *J* = 12.4, 3.1 Hz, 1H). ^{13}C NMR (125 MHz, $CDCl_3$) δ 144.25, 133.07, 131.80, 126.32, 110.17, 108.19,

62.04, 57.94, 36.51, 28.34, 26.41, 26.04. HRMS (ESI): calculated $C_{14}H_{21}Br_2N_2$ $[M+H]^+$: $^{79}Br/^{79}Br$ 375.0066, found: 375.0073.

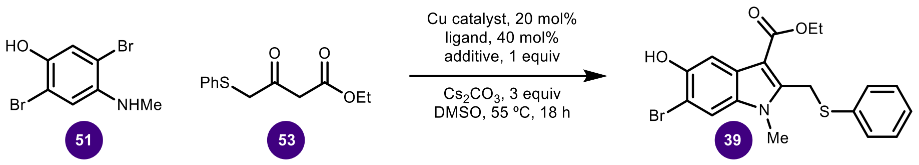

Stock solutions, or suspensions, were prepared as shown in the Table. In an inert atmosphere glovebox, reagents were weighed and dissolved or suspended in anhydrous degassed DMSO to achieve the concentration listed in Table. Stock solutions of reagents were stirred until either a clear solution or a uniform slurry was achieved. A 24-well aluminum microvial plate (Analytical Sales & Services cat. no. 25243) was equipped with oven-dried shell vials (Analytical Sales & Services cat. no. 884001) then moved to the glove box. Stock solutions were dosed to the appropriate shell vials according to the plate map shown in Table using single channel micropipetters. A parylene-coated stir dowel (Analytical Sales & Services cat. no. 13258) was then added to each vial. The microvial plate was sealed, removed from the glove box, and heated to 55 ˚C for 18 h with stirring on a ChemGlass stirring hotplate.

The reactions were quenched by opening the reaction block and adding 100 µL saturated aqueous ammonium chloride solution and 420 µL EtOAc. Reactions were extracted by resealing the plate and shaking manually. From each reaction, an aliquot of the quenched reaction mixture was added into a 96-well polypropylene collection plate (Analytical Sales & Services cat. no. 17P687). The solvent was evaporated by nitrogen blow down on the analytical plate. An acetonitrile solution of caffeine as internal standard (0.005 M, 800 µL) was added, and mixed by pipetting up and down. The reactions were then analyzed by UPLC-MS.The assay yields was produced by measuring the UV absorbance of **39** relative to the caffeine internal standard.

Reagents	C_{stock} (M)	V_{dose} (µL)	Wells
**51**	0.6	16.7	All
**53**	3.0	16.7	All
Cs_2CO_3	2.4	16.7	All
Blank	-	16.7	A1,3,5-D1,3,5
$MgSO_4$	0.6	16.7	A2,4,6-D2,4,6<br>A1,2-D1,2

2-(1H-tetrazol-1-yl)acetic acid (L1)	0.24	16.7	A3,4-D3,4
2,6-dimethylanilino(oxo)acetic acid (L2)	0.24	16.7	A5,6-D5,6
Copper(I) iodide	0.12	16.7	A1-A6
Copper(I) bromide	0.12	16.7	B1-B6
Tetrakisacetonitrile copper(I) triflate	0.12	16.7	C1-C6
Copper(II) acetate	0.12	16.7	D1-D6

**Reagents**	**Reaction conc. (M)**	**Color**
**51**	0.100	
**53**	0.500	
Cs_2CO_3	0.400	
Blank	-	
Blank	-	
$MgSO_4$	0.100	
L1	0.040	
L2	0.040	
CuI	0.020	
CuBr	0.020	
CuOTf	0.020	
$Cu(OAc)_2$	0.020	

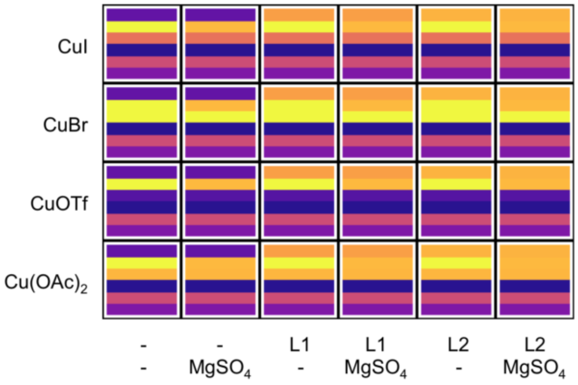

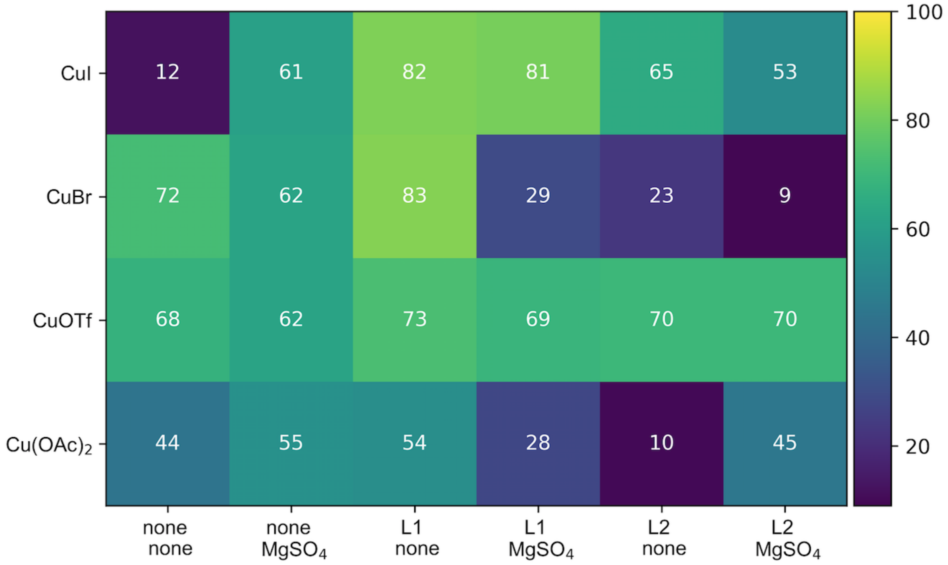
CuI
CuBr
CuOTf
$Cu(OAc)_2$
12 61 82 81 65 53
72 62 83 29 23 9
68 62 73 69 70 70
44 55 54 28 10 45
none none
none $MgSO_4$
L1 none
L1 $MgSO_4$
L2 none
L2 $MgSO_4$
100
80
60
40
20

3. Supplementary Figures

Spectra

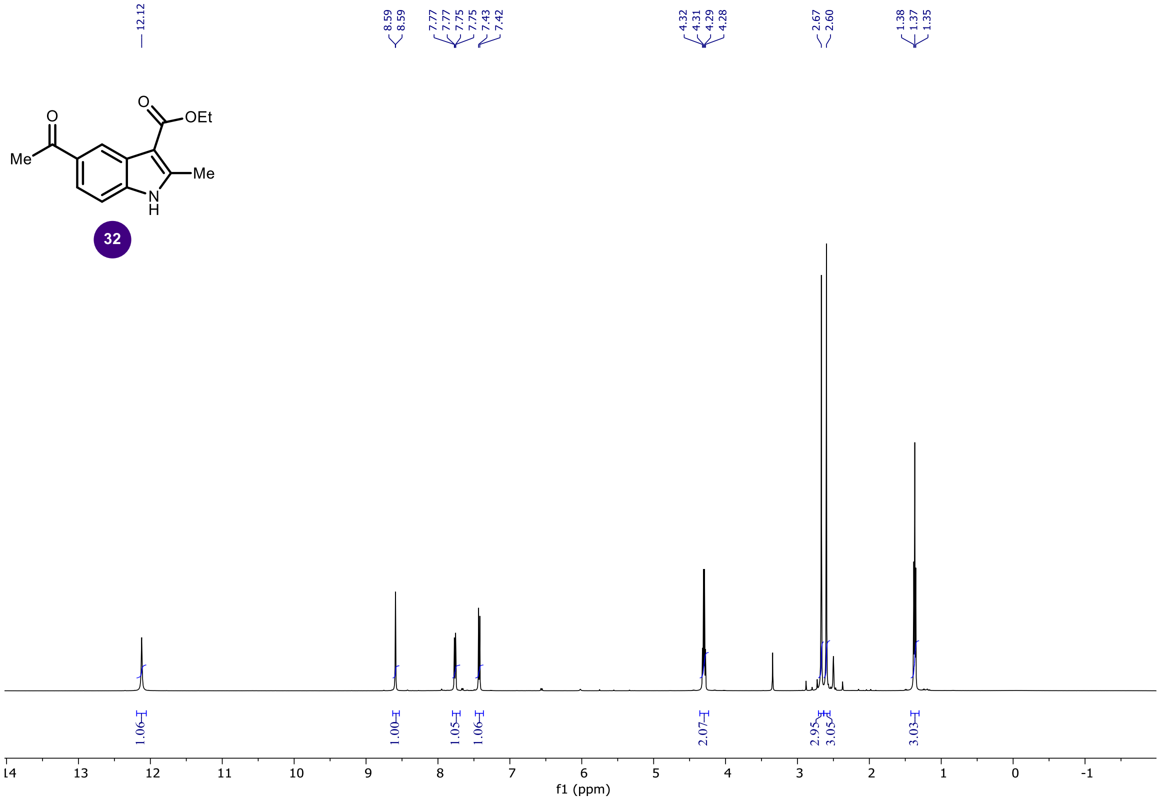

Supplementary Figure 1. ^{1}H NMR of **32** in $(CD_3)_2SO$ at 25 ºC.

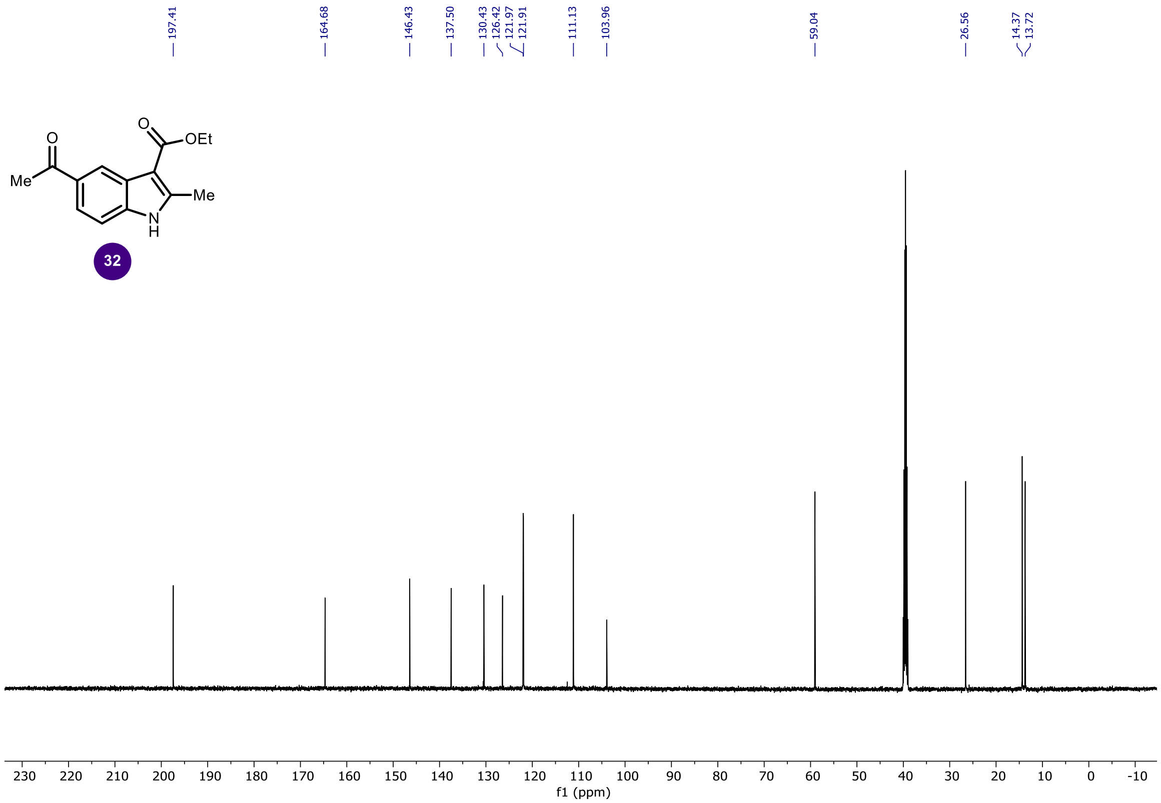

Supplementary Figure 2. ^{13}C NMR of **32** in $(CD_3)_2SO$ at 25 ºC.

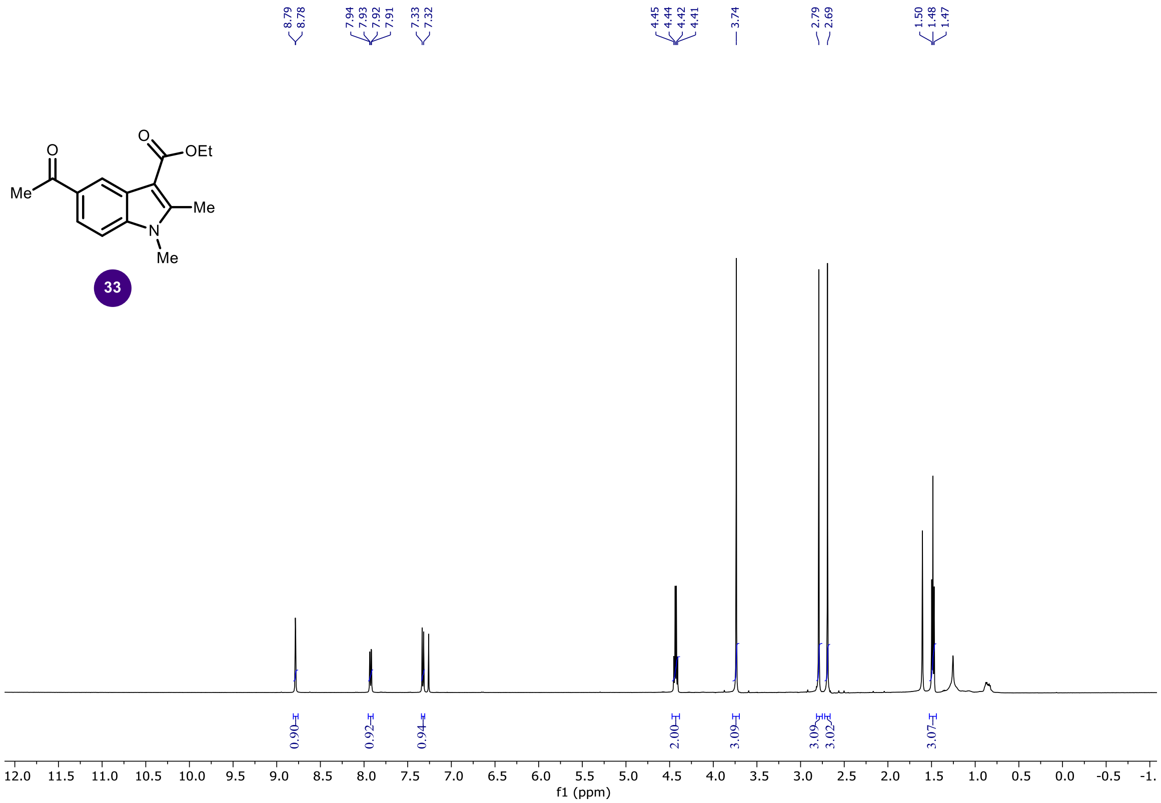

Supplementary Figure 3. ^{1}H NMR of **33** in $CDCl_3$ at 25 °C.

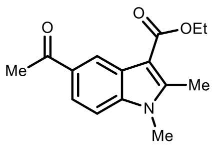

33

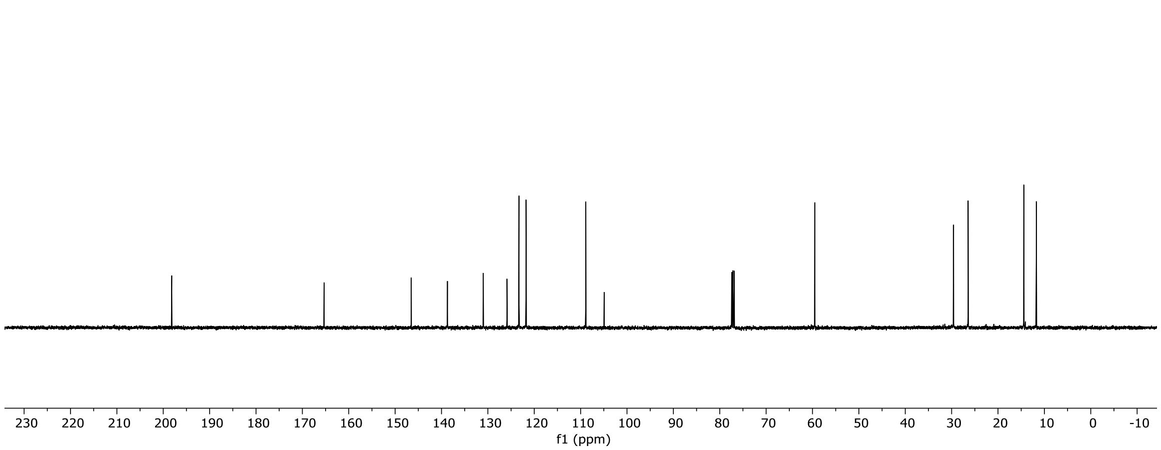

Supplementary Figure 4. ^{13}C NMR of **33** in $CDCl_3$ at 25 ºC.

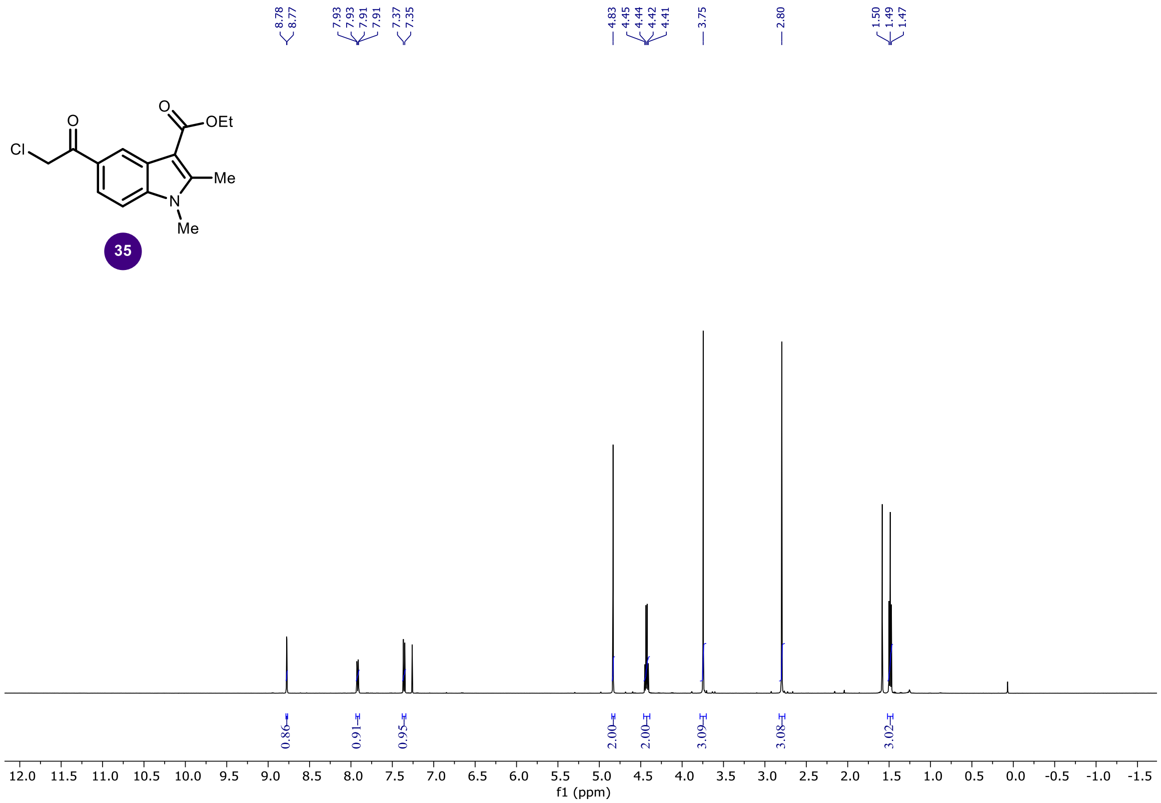

Supplementary Figure 5. ^{1}H NMR of **35** in $CDCl_3$ at 25 ºC.

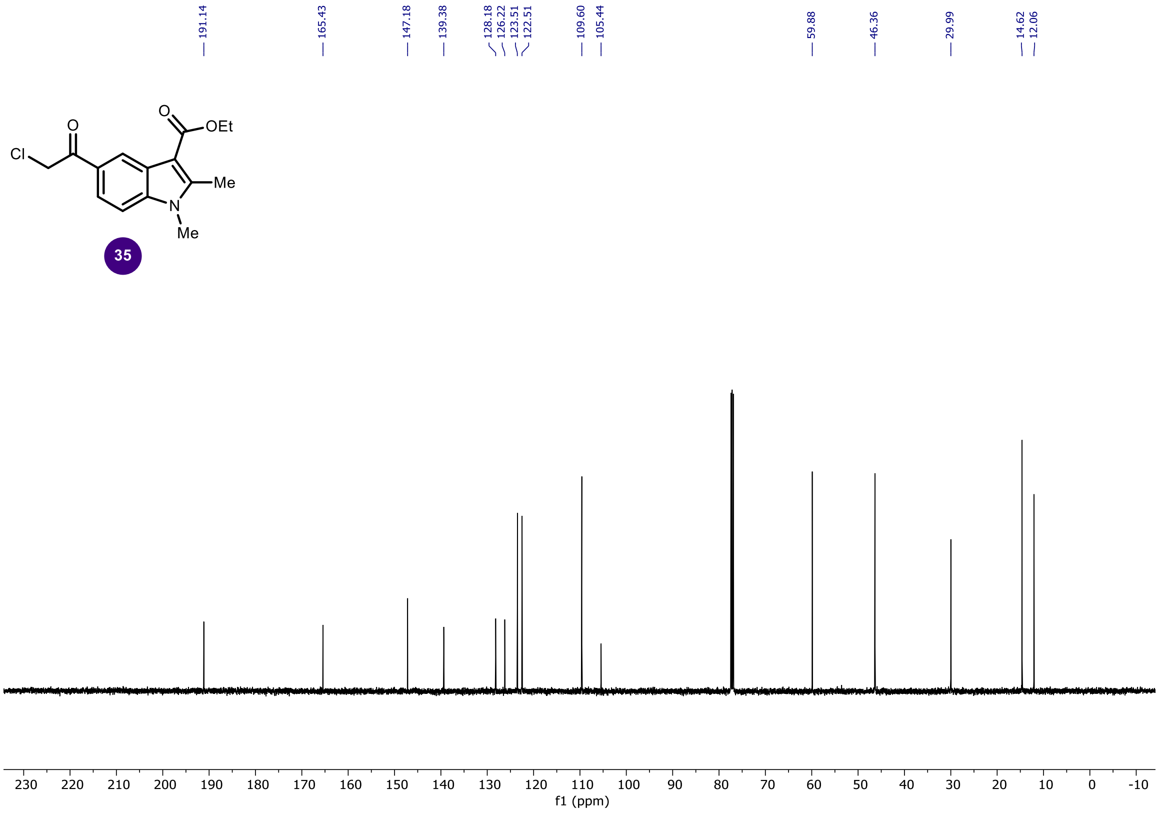

Supplementary Figure 6. ^{13}C NMR of **35** in $CDCl_3$ at 25 ºC.

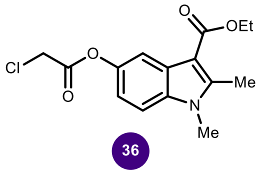

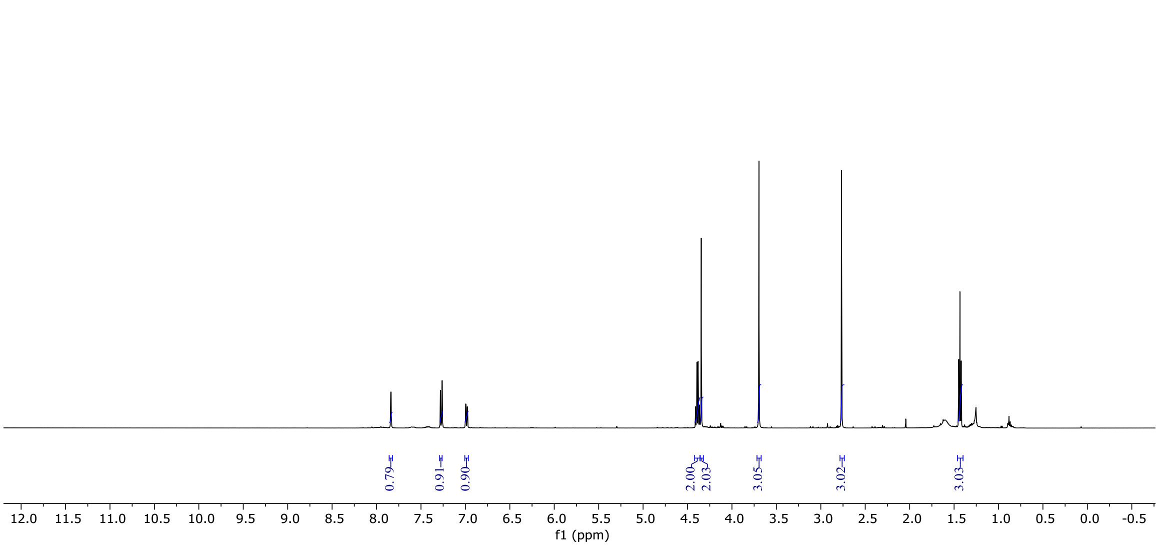

Supplementary Figure 7. ^{1}H NMR of **36** in $CDCl_3$ at 25 °C.

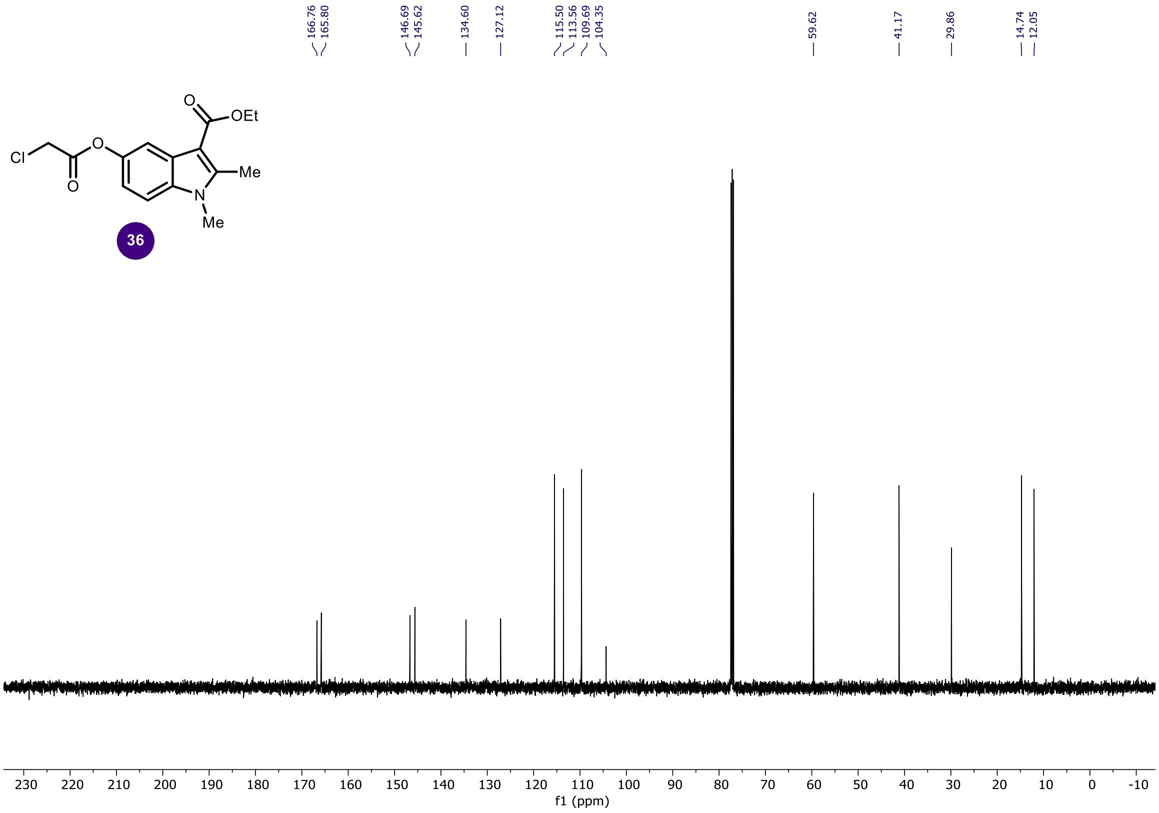

Supplementary Figure 8. ^{13}C NMR of **36** in $CDCl_3$ at 25 ºC.

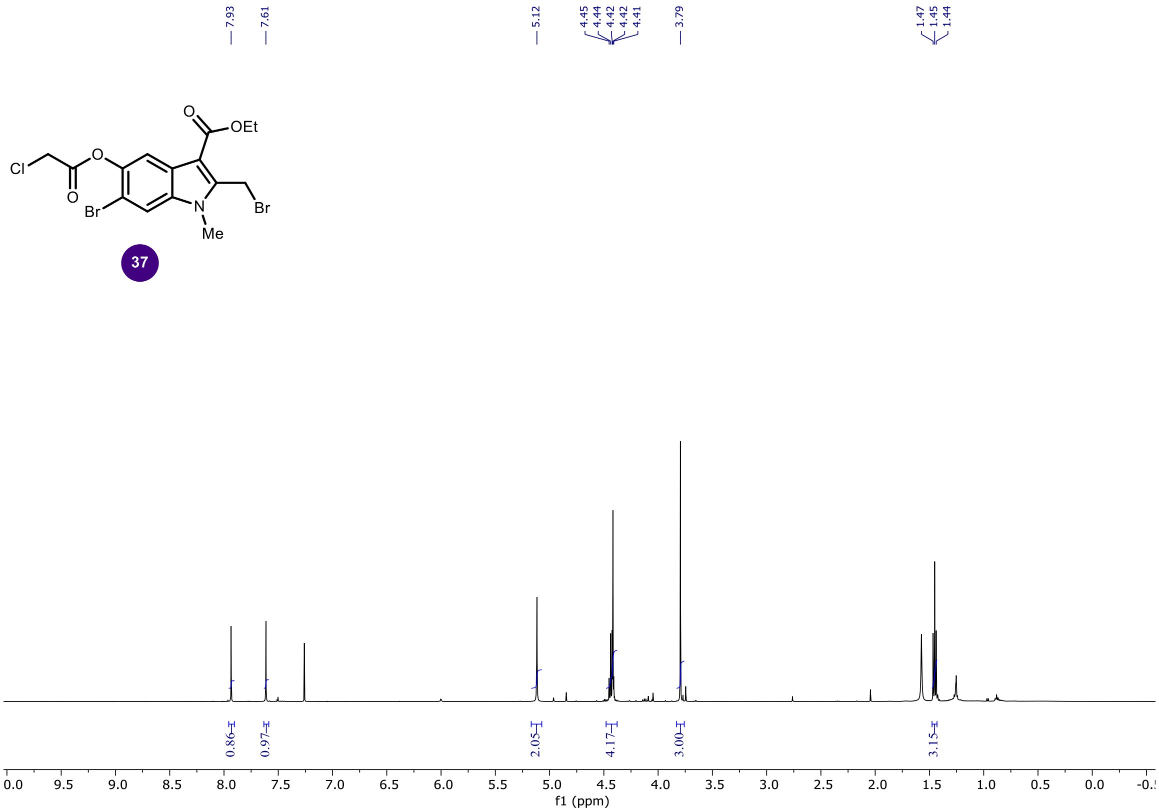

Supplementary Figure 9. ^{1}H NMR of **37** in $CDCl_3$ at 25 °C.

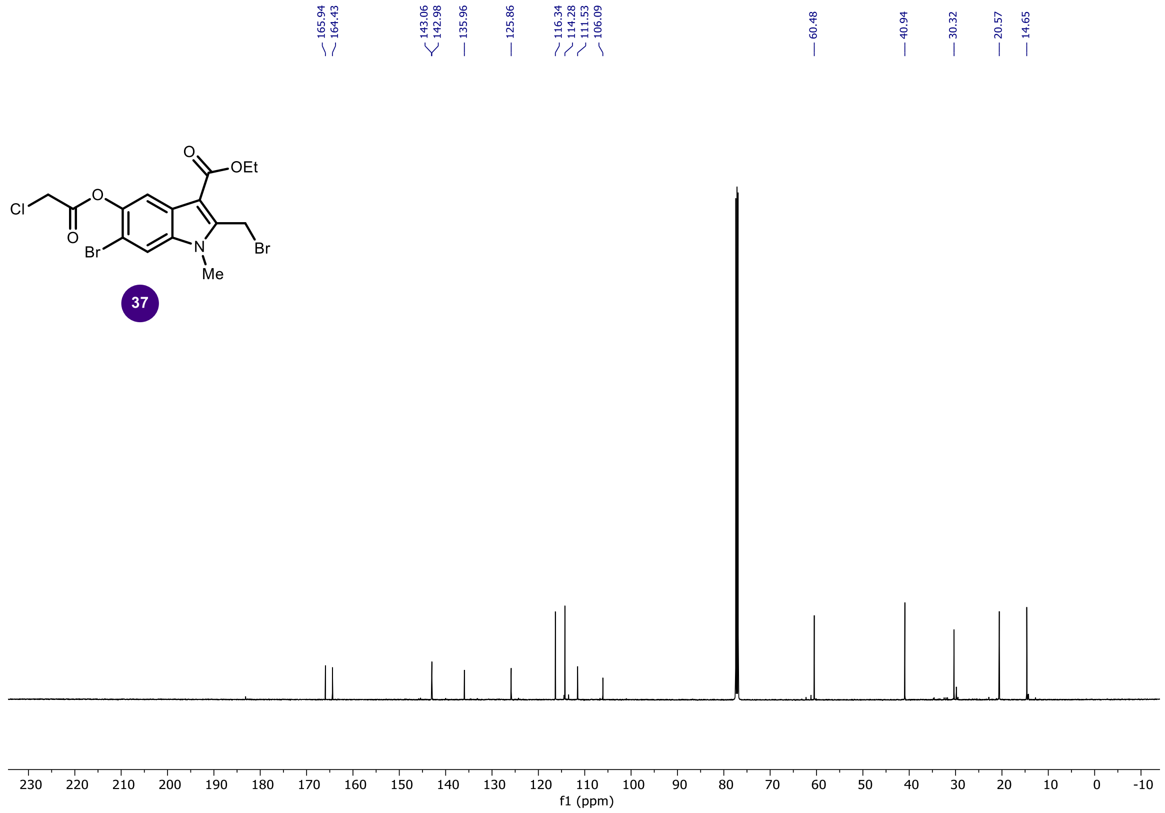

Supplementary Figure 10. ^{13}C NMR of **37** in $CDCl_3$ at 25 ºC.

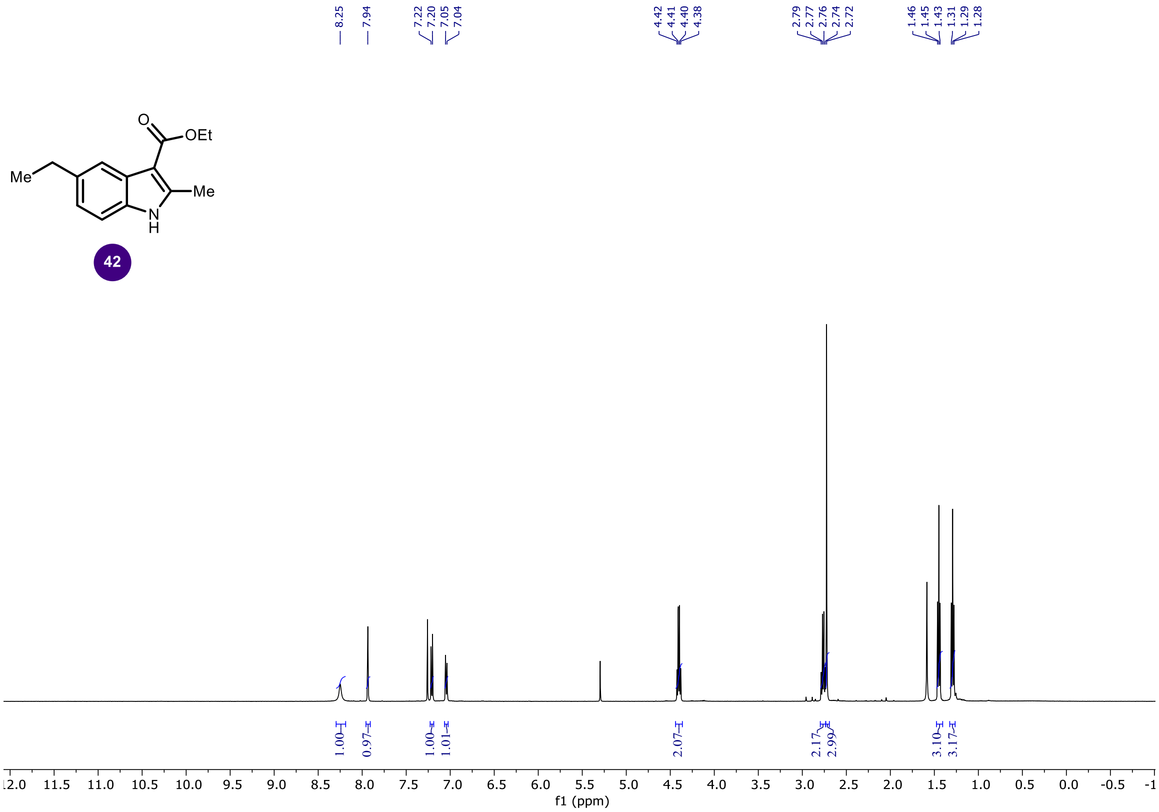

Supplementary Figure 11. ^{1}H NMR of **42** in $CDCl_3$ at 25 ºC.

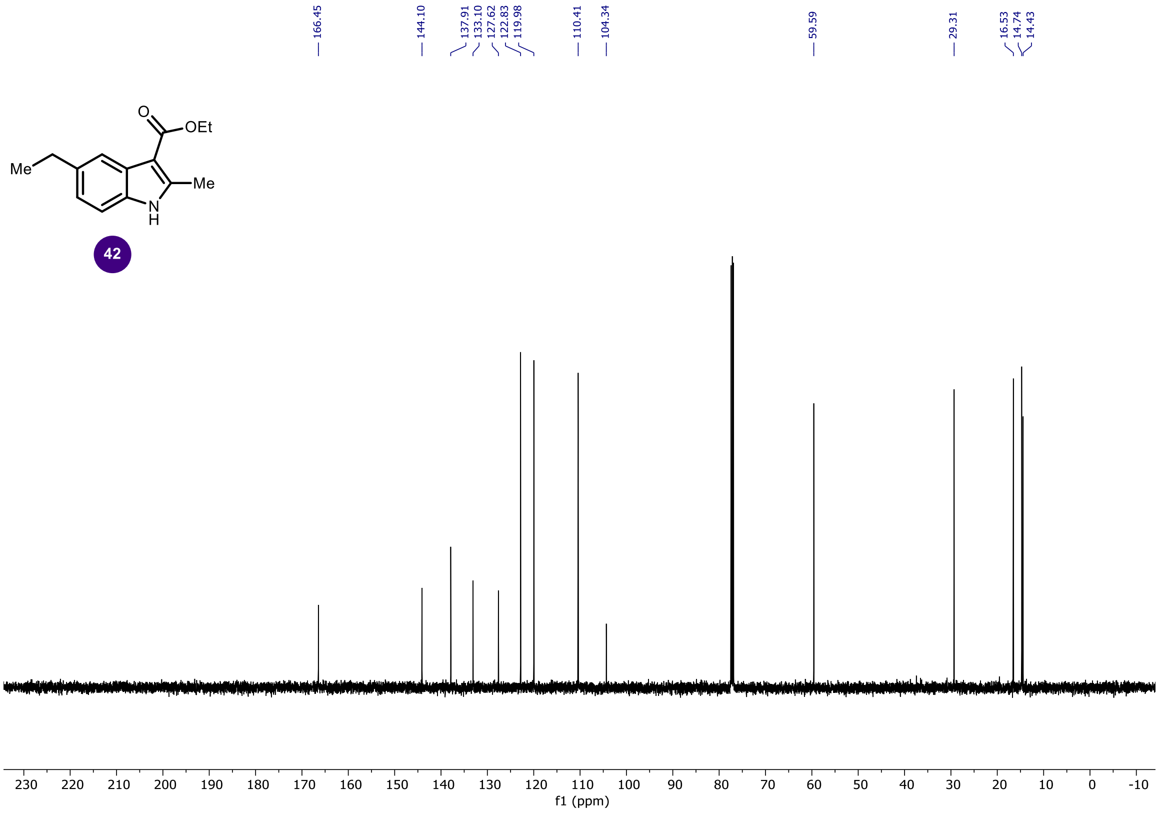

Supplementary Figure 12. ^{13}C NMR of **42** in $CDCl_3$ at 25 ºC.

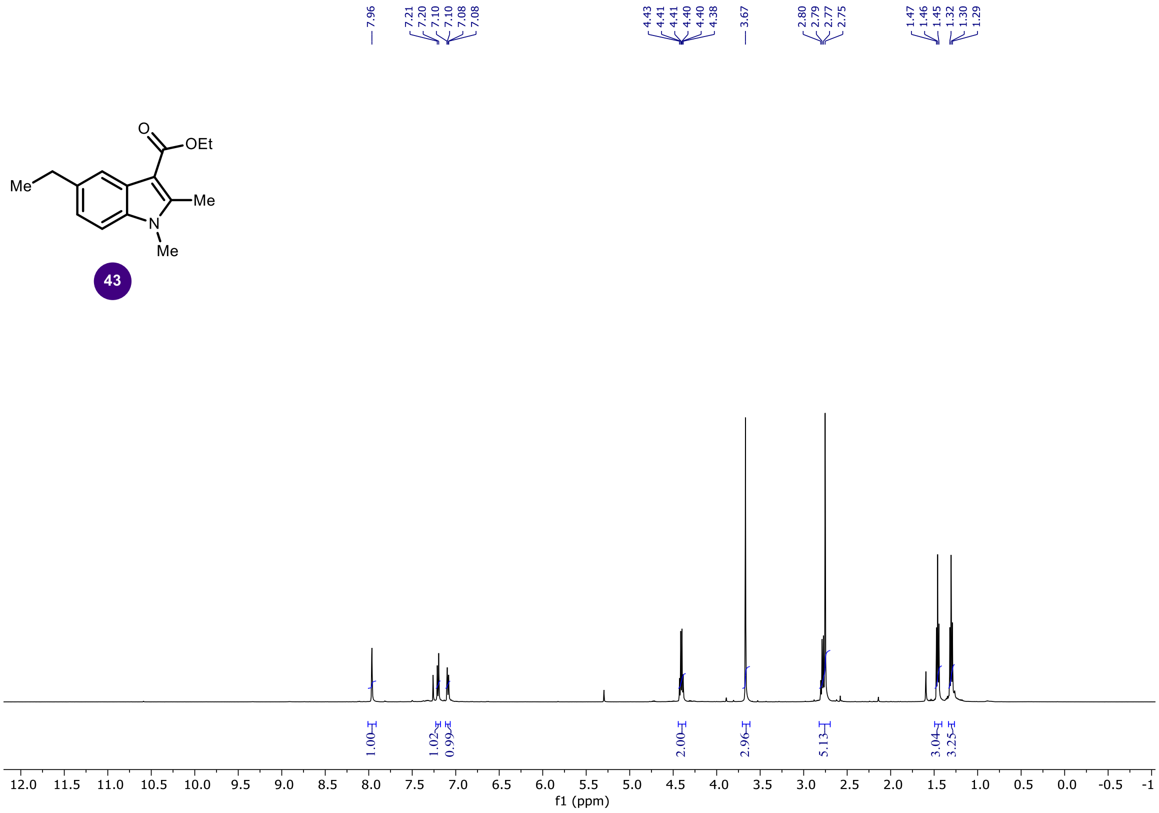

Supplementary Figure 13. ^{1}H NMR of **43** in $CDCl_3$ at 25 ºC.

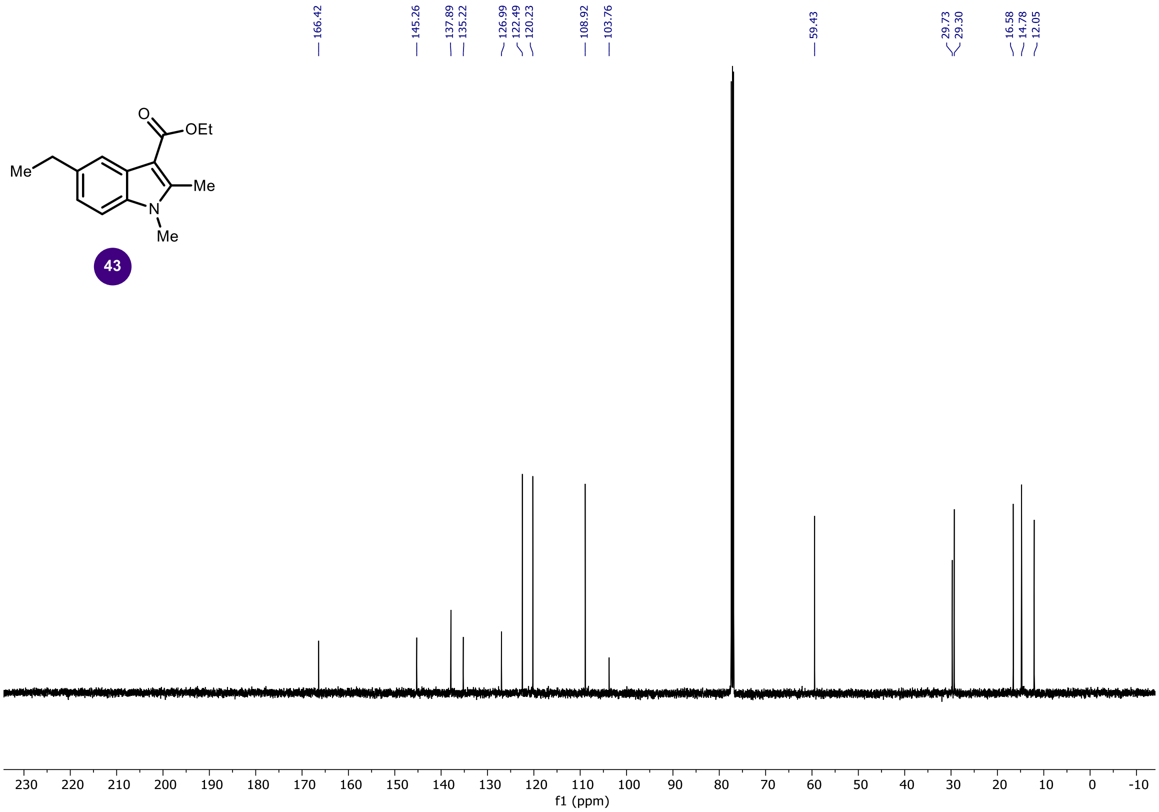

Supplementary Figure 14. ^{13}C NMR of **43** in $CDCl_3$ at 25 ºC.

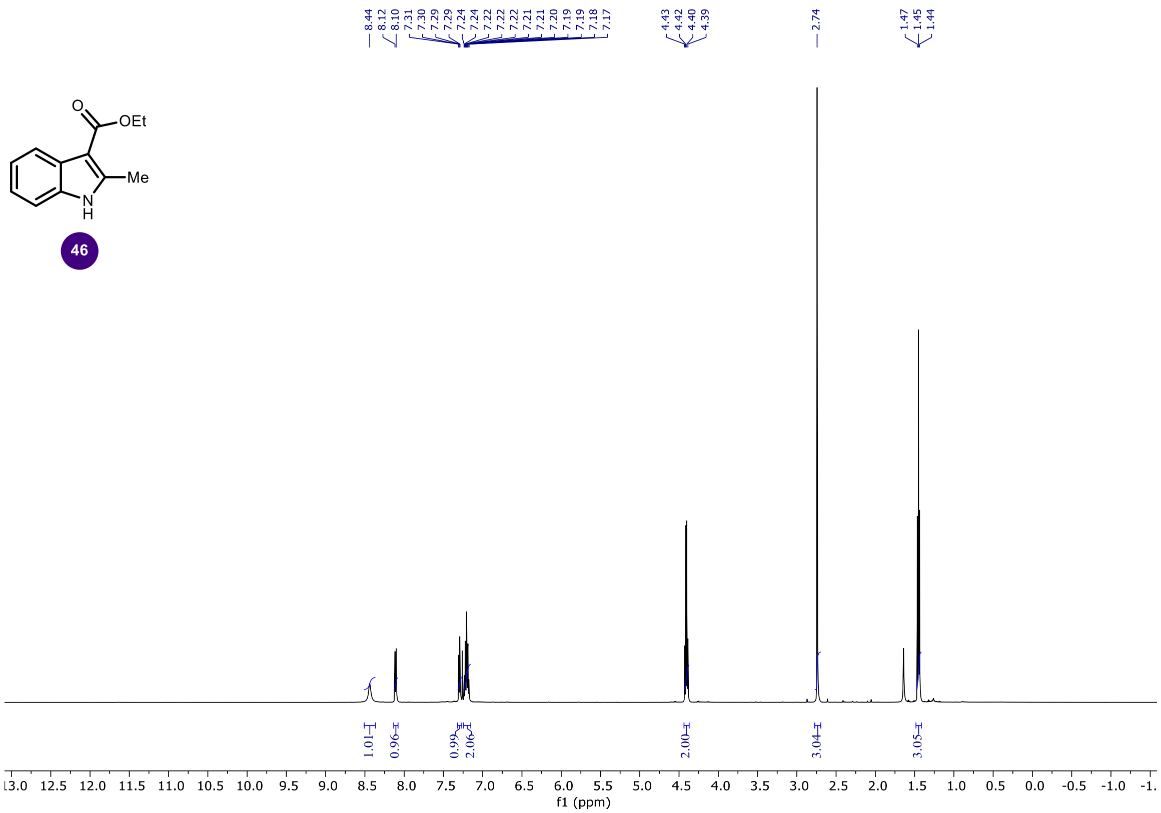

Supplementary Figure 15. ^{1}H NMR of **46** in $CDCl_3$ at 25 ºC.

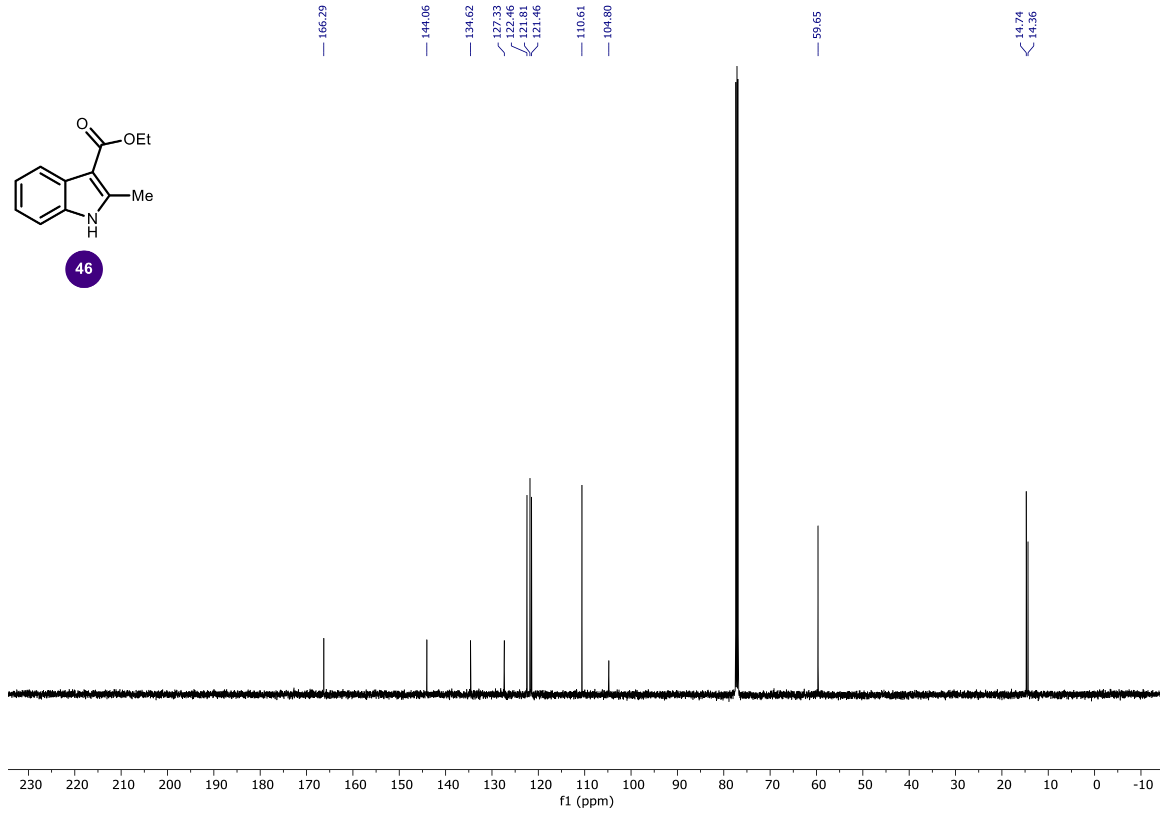

Supplementary Figure 16. ^{13}C NMR of **46** in $CDCl_3$ at 25 ºC.

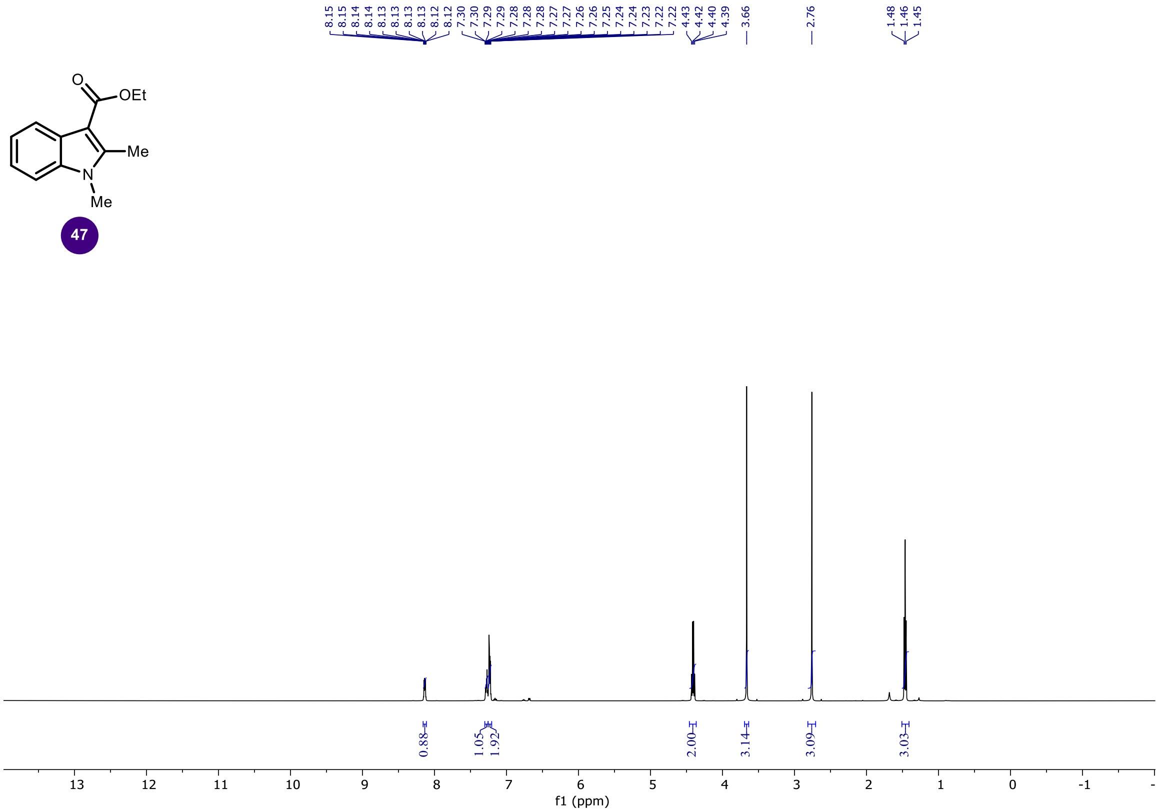

Supplementary Figure 17. ^{1}H NMR of **47** in $CDCl_3$ at 25 ºC.

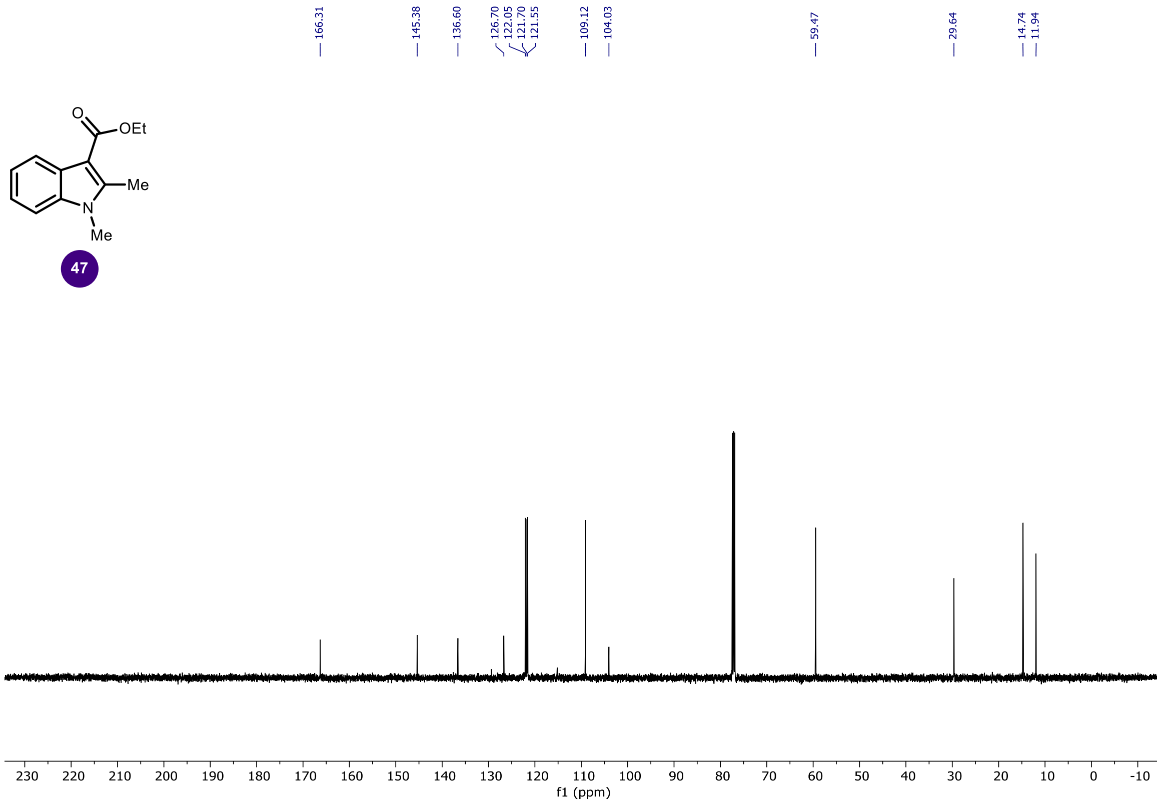

Supplementary Figure 18. ^{13}C NMR of **47** in $CDCl_3$ at 25 ºC.

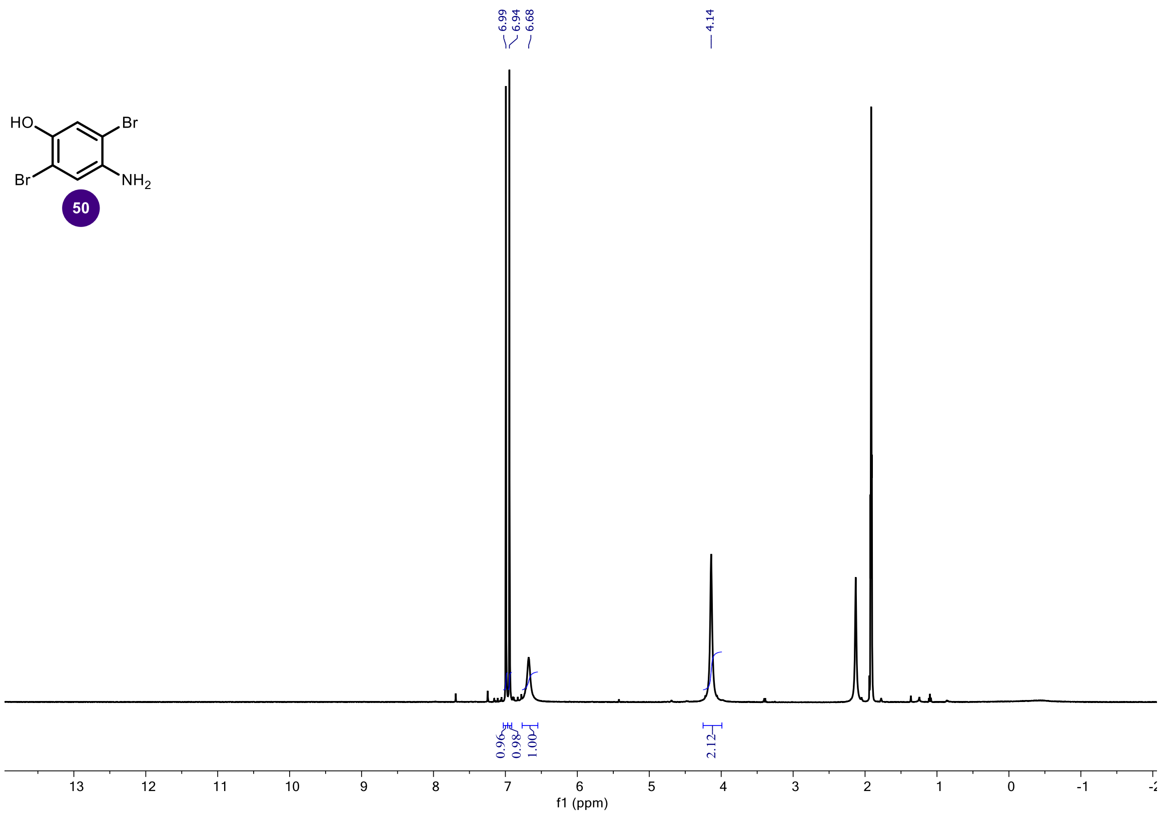

Supplementary Figure 19. ^{1}H NMR of **50** in CD_3CN at 25 °C.

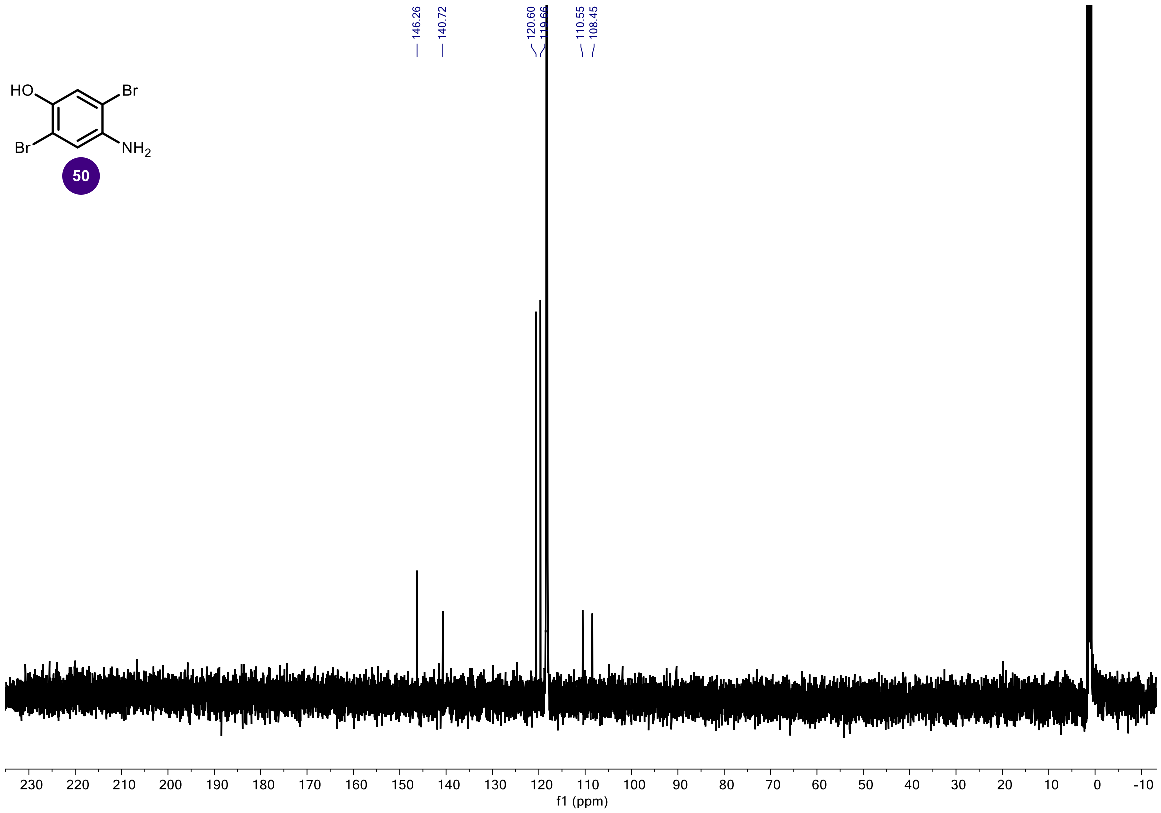

Supplementary Figure 20. ^{13}C NMR of **50** in CD_3CN at 25 ºC.

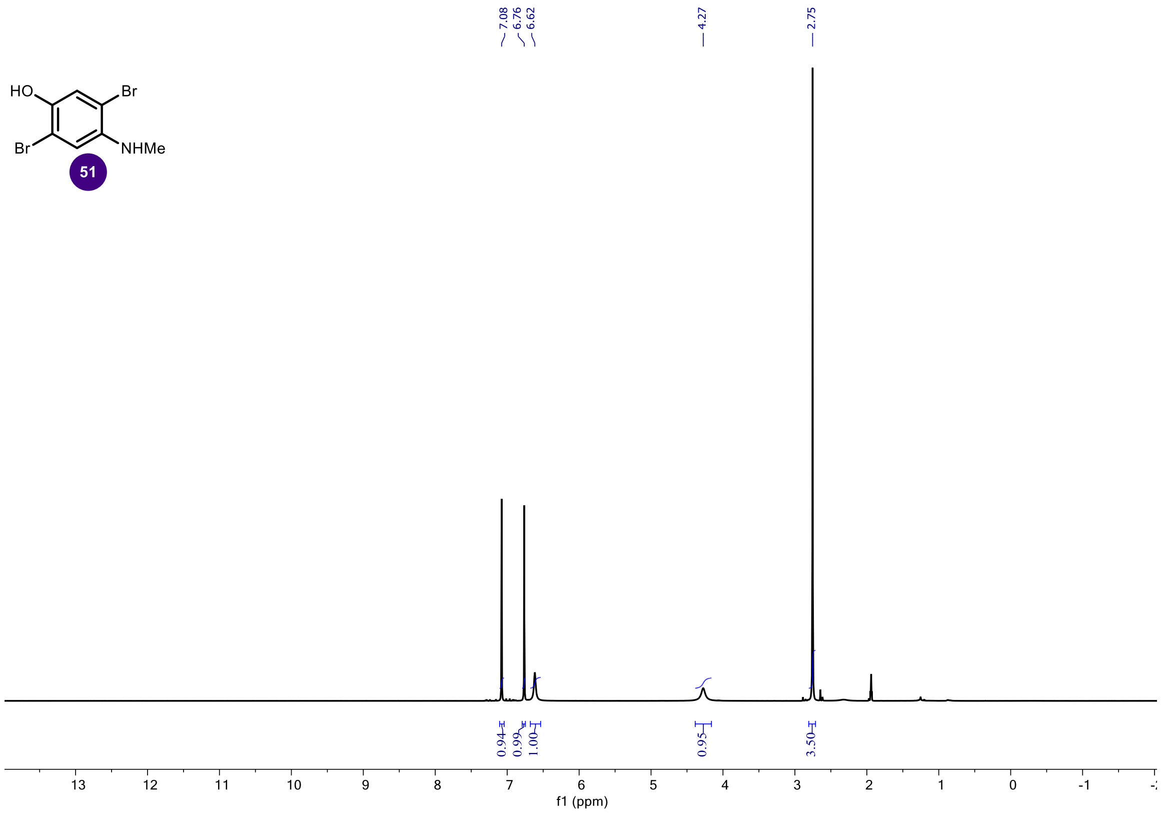

Supplementary Figure 21. ^{1}H NMR of **51** in CD_3CN at 25 ^oC.

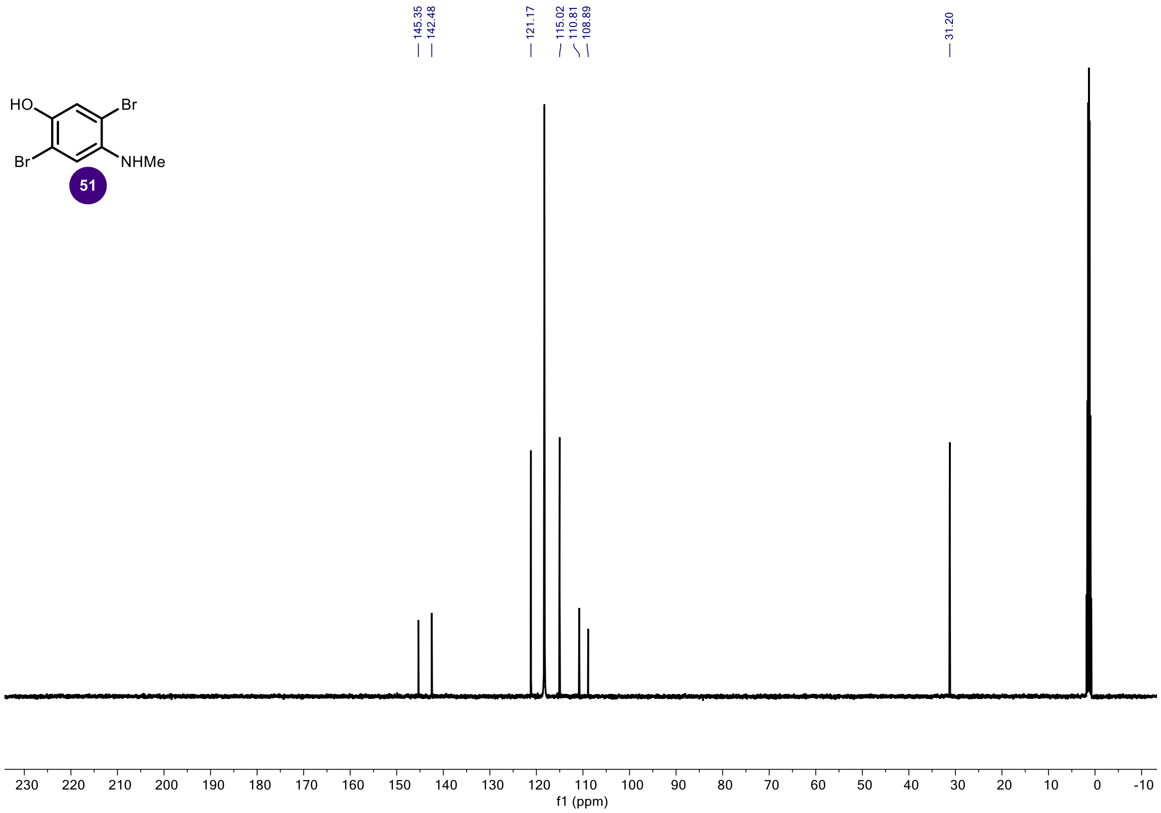

Supplementary Figure 22. ^{13}C NMR of **51** in CD_3CN at 25 °C.

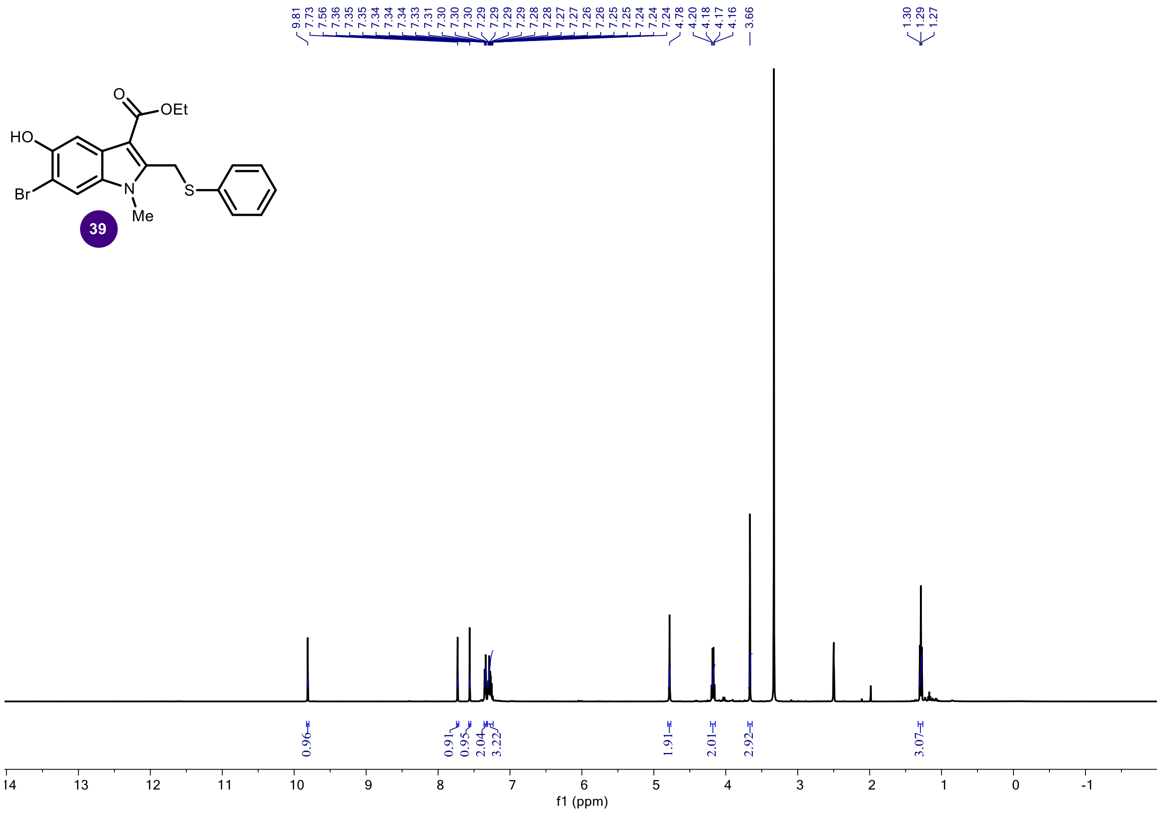

Supplementary Figure 23. ^{1}H NMR of **39** in $(CD_3)_2SO$ at 25 ºC.

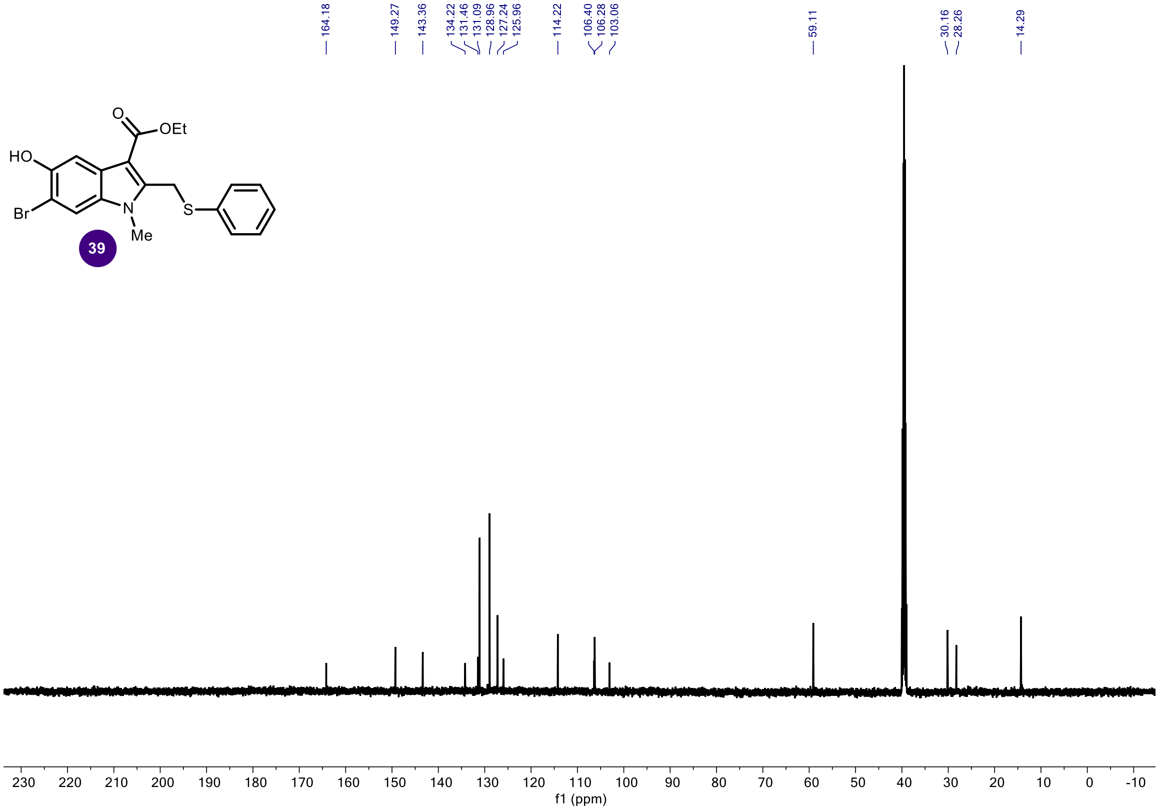

Supplementary Figure 24. ^{13}C NMR of **39** in $(CD_3)_2SO$ at 25 ºC.

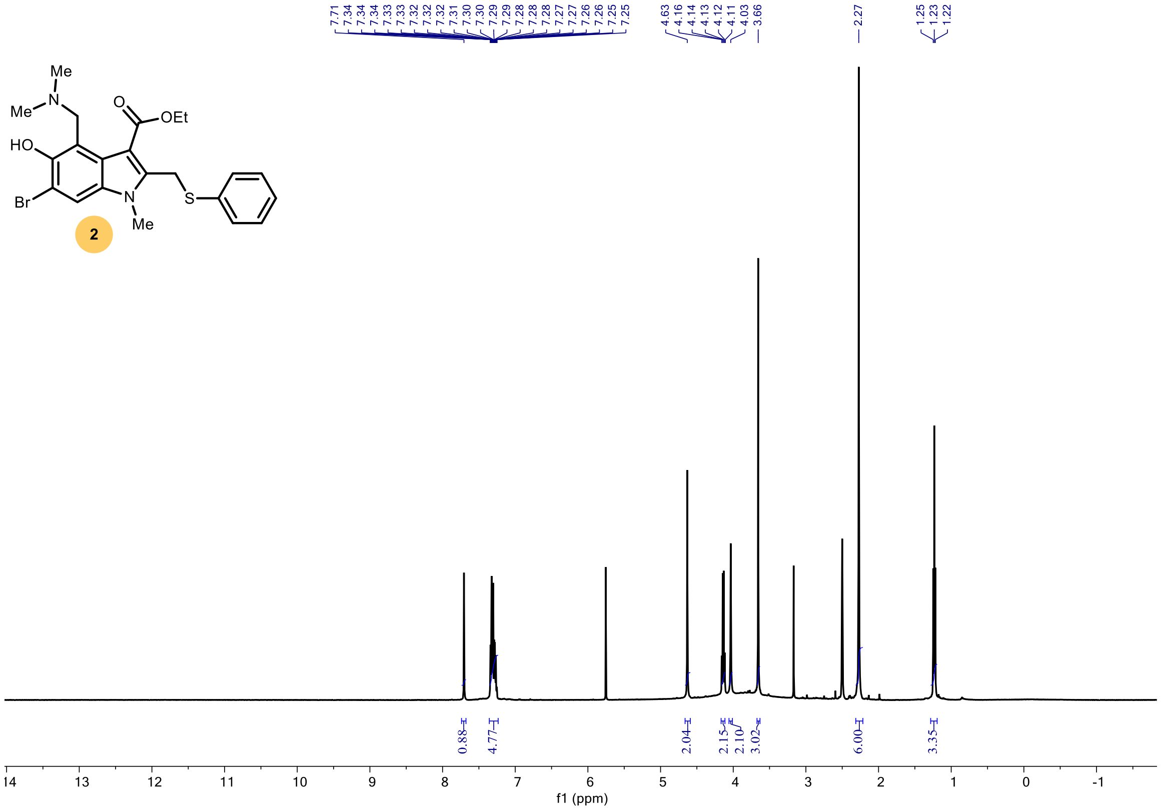

Supplementary Figure 25. ^{1}H NMR of **2** in $(CD_3)_2SO$ at 25 ^{o}C.

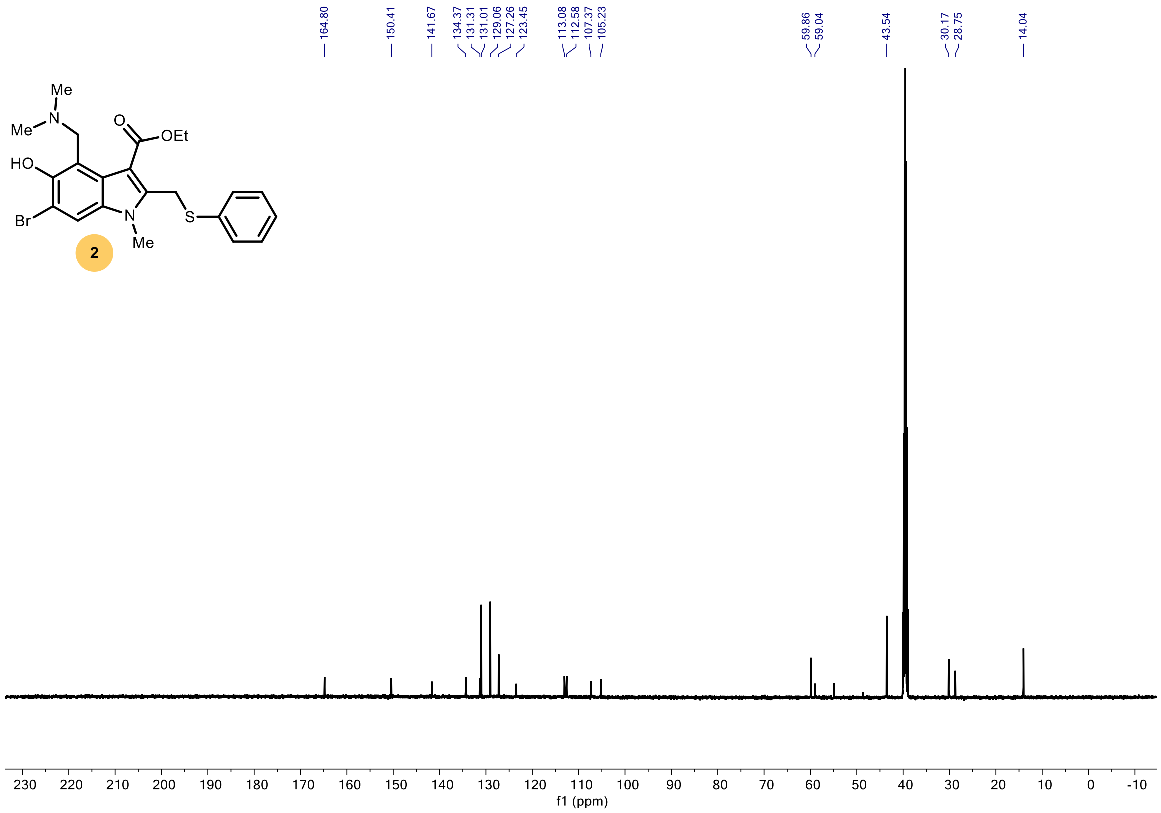

Supplementary Figure 26. ^{13}C NMR of **2** in $(CD_3)_2SO$ at 25 °C.

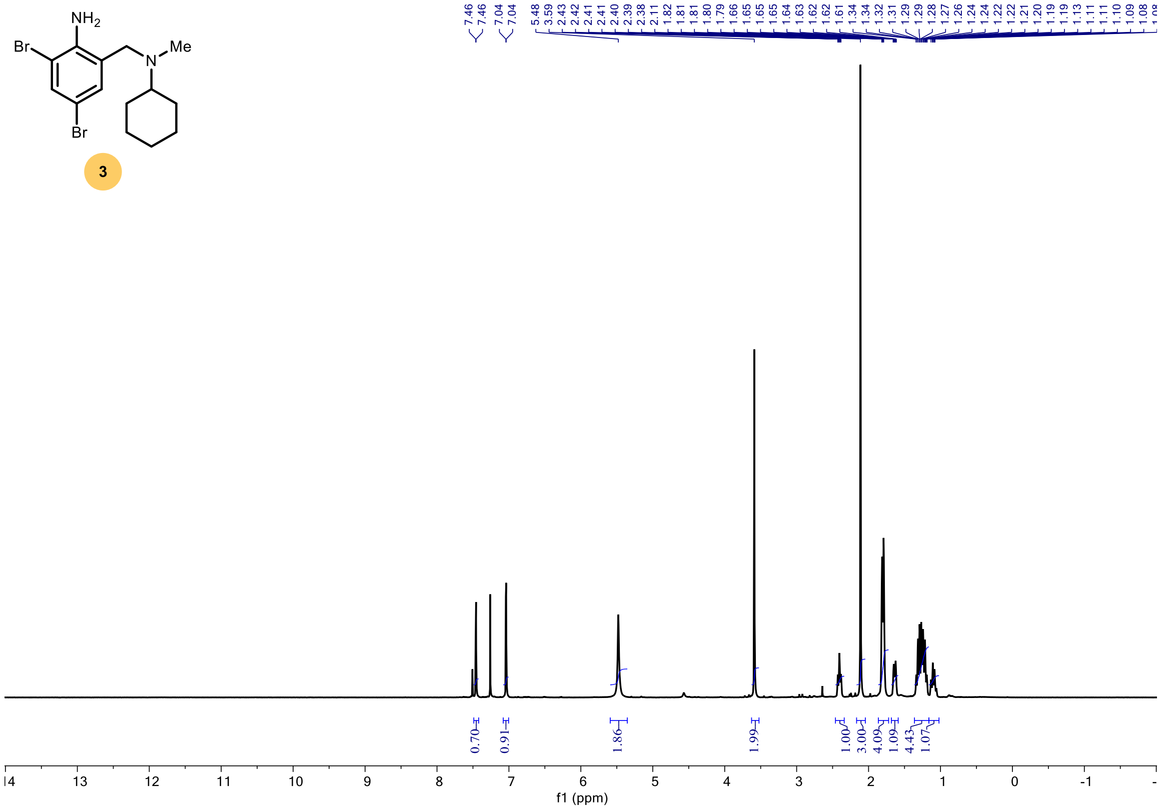

Supplementary Figure 27. ^{1}H NMR of **3** in $CDCl_3$ at 25 ºC.

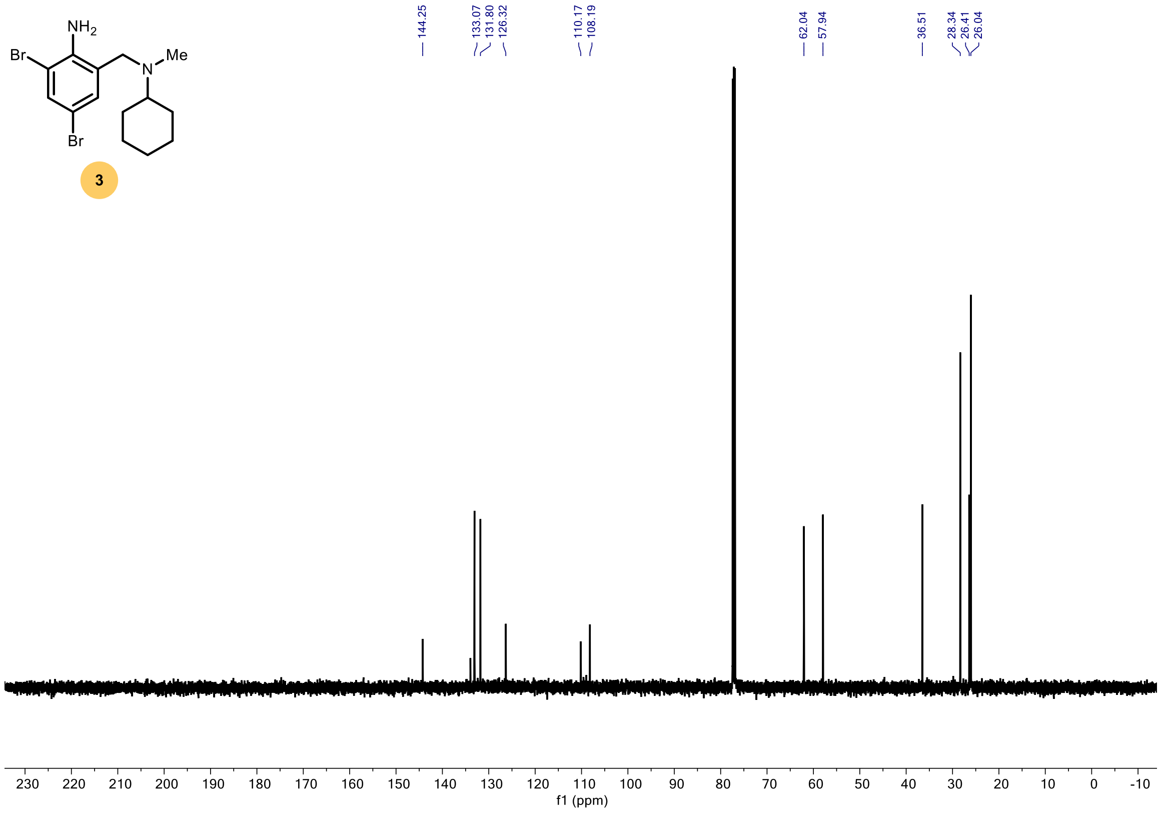

Supplementary Figure 28. ^{13}C NMR of **3** in $CDCl_3$ at 25 ºC.

SYNTHIA™ searches and predicted routes

The generic procedure for performing SYNTHIA searches for each drug is as follows.

For each drug, the SMILES of each starting material found in existing patents were compiled and added to the default price equalizer scoring function using the HIDE_SMILES parameter, which was multiplied by -1000 to penalize routes that contained known starting materials. The max reagent price was set to $100 or $1000, the popularity metric was set to 15 or 25, beam search minimum search width was set between 100 and 200, beam search max reactions per product was set between 20 and 30, and the search completed 5000 iterations to return 50 routes for each target molecule. All other parameters were kept as their default values.

For the crowd-sourcing of routes, each team member was assigned to collect all patented or published routes to one of the synthetic targets (**1**–**12**). For each of the reported routes, reaction components were encoded in a comma-separated values (.csv) file with starting materials and products documented using simplified molecular input line entry system (SMILES) strings, and reaction conditions documented as text. The format of the csv is as follows: There are four columns, the first column contains the route number, the second column contains the reaction step number from within that route, the third column contains a qualifier [‘reactant’, ‘product’, ‘conditions’, ‘protection recommended’, ‘doi/patent number’, ‘source (literature | SYNTHIA)’], and finally, the fourth column contains the value corresponding to the qualifier documented in the third column. Each reaction must contain a reactant and a product as a minimum data entry. A reactant that is a product in a previous reaction in the same or different route converges into the same node in the graphical interface. All encoded routes to a synthetic target are compiled in a single .csv, and used as an input to a python script to generate the interface, which can be accessed for free at http://covidroutes.cernaklab.com/.

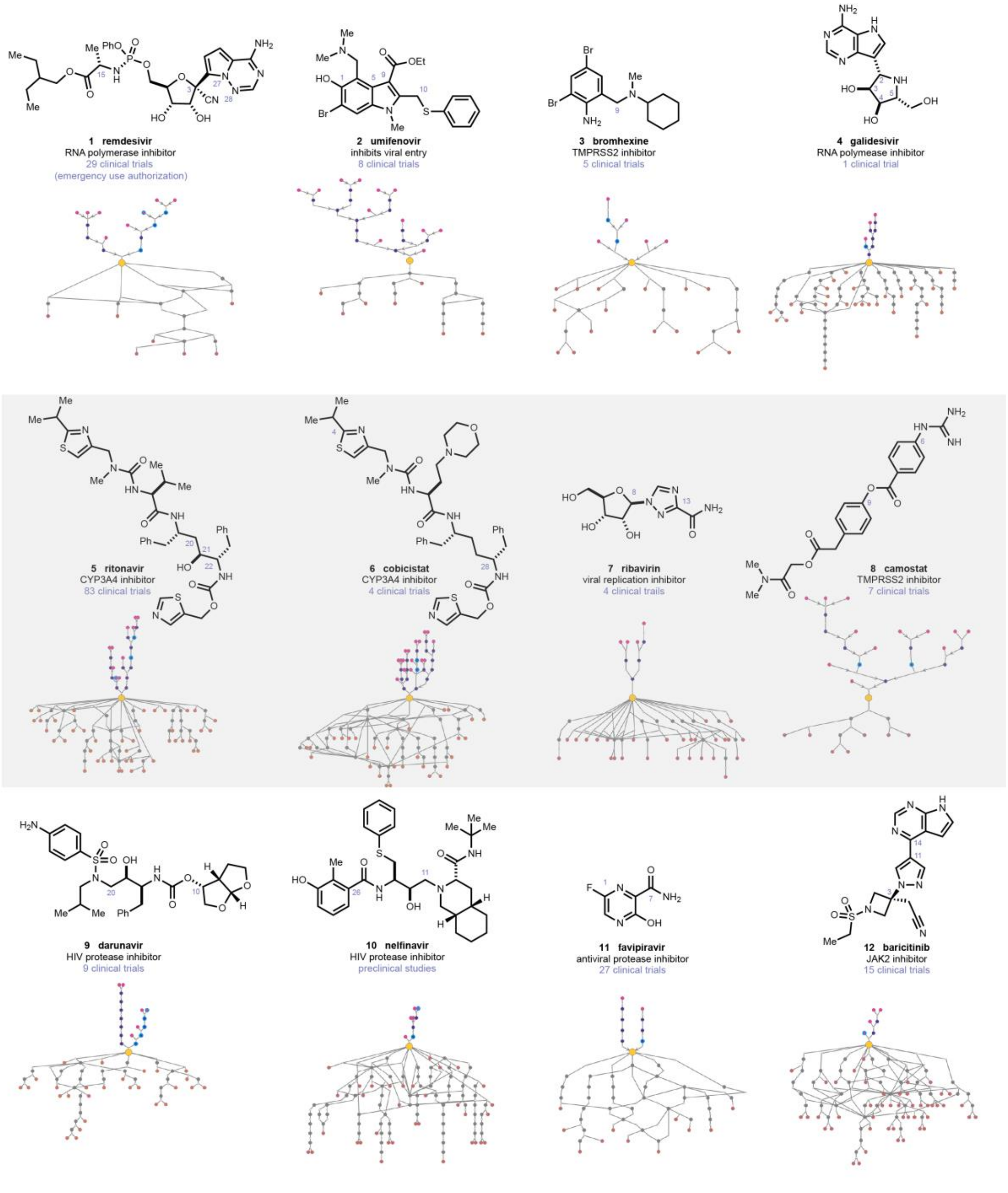

Figure 2. Therapeutics being evaluated for COVID-19 and their retrosynthetic analysis networks**.**

Remdesivir

Analysis type: Automatic Retrosynthesis

Rules: none selected

Filters: none selected

Max. paths returned: 50

Max. iterations: 5000

Buyable:

1. Max. molecular weight - 1000 g/mol

2. Max. price - 1000 $/g

Known:

1. Max. molecular weight - 1000 g/mol

2. Min. popularity - 15

Shorter paths: no

Pathway linearity: COMBO

Protecting groups: LESS

Reaction scoring formula: 20+1000000*(FILTERS+CONFLICT+NON_SELECTIVITY)+40*PROTECT

Chemical scoring formula: SMALLER^3,SMALLER^1.5

Min. search width: 100

Max. reactions per product: 20

Path Score: 1000814.47

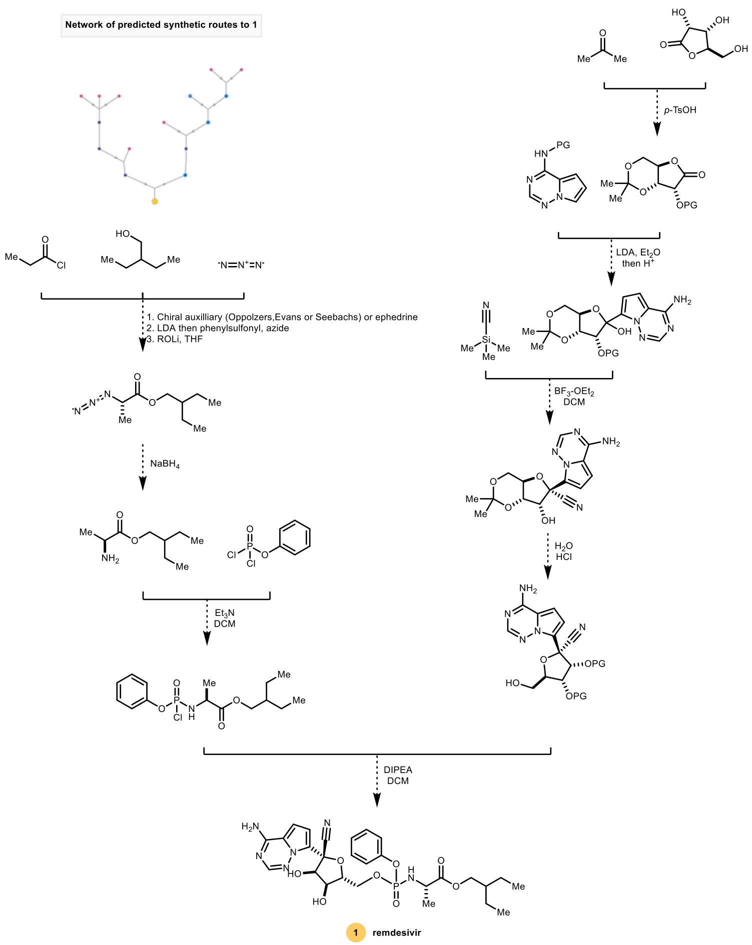

Supplementary Figure 29. SYNTHIA™ predicted routes to **1**. PG = Protection group. SYNTHIA™ recommends when groups should be protected, but does not propose exact protecting groups. In instances where SYNTHIA™ does propose an exact protecting group, the proposed protecting group is drawn.

Umifenovir-1

Analysis type: Automatic Retrosynthesis

Rules: none selected

Filters: Multicut, Strategies

Max. paths returned: 50

Max. iterations: 5000

Buyable:

1. Max. molecular weight - 1000 g/mol

2. Max. price - 10 $/g

Known:

1. Max. molecular weight - 1000 g/mol

2. Min. popularity - 9999

Shorter paths: no

Avoided SMILES: ’CCOC(=O)c1c(C)[nH]c2ccc(O)cc12’,’O=C1C=CC(=O)C(Br)=C1’,’CCOC(=O)CC(=O])c1ccc(O)cc1’,’O=[N+]([O-])c1ccc(O)cc1I’

Pathway linearity: COMBO

Protecting groups: LESS

Reaction scoring formula: TUNNEL_COEF*FGI_COEF*20+1000000*(FILTERS+CONFLICT+NON_SELECTIVITY)+40*PROTECT+1000000*HIDE_SMILES(’CCOC(=O)c1c(C)[nH]c2ccc(O)cc12’,’O=C1C=CC(=O)C(Br)=C1’,’CCOC(=O)CC(=O)CCl’,’O=C1C=CC(=O)C=C1’,’O=[N+]([O-])c1ccc(O)cc1’,’O=[N+]([O-])c1ccc(O)cc1I’)

Chemical scoring formula: SMALLER^3,SMALLER^1.5

Min. search width: 100

Max. reactions per product: 20

Path Score: 164.63 & 225.44

Umifenovir-2

Analysis type: Automatic Retrosynthesis

Rules: none selected

Filters: Cut All Heterocycles, Multicut, Strategies

Max. paths returned: 50

Max. iterations: 5000

Buyable:

1. Max. molecular weight - 1000 g/mol

2. Max. price - 50 $/g

Known:

1. Max. molecular weight - 1000 g/mol

2. Min. popularity - 15

Inventory:

1. Max. molecular weight - 1000 g/mol

Shorter paths: yes

Avoided SMILES: ’CCOC(=O)c1c(C)[nH]c2ccc(O)cc12’,’CCOC(=O)CC(C)=O’,’O=C1C=CC(=O)C(Br)=])c1ccc(O)cc1’,’O=[N+]([O-])c1ccc(O)cc1I’

Avoided keywords: ’nenitzescu’

Pathway linearity: COMBO Protecting groups: LESS

Reaction scoring formula: 100*(TUNNEL_COEF*FGI_COEF*20+1000000*(FILTERS+CONFLICT+NON_SELECTIVITY)+40*PROTECT+1000000*HIDE_SMILES(’CCOC(=O)c1c(C)[nH]c2ccc(O)cc12’,’CCOC(=O)CC(C)=O’,’O=C1C=CC(=O)C(Br)=C1’,’CCOC(=O)CC(=O)CCl’,’O=C1C=CC(=O)C=C1’,’O=[N+]([O-])c1ccc(O)cc1’,’O=[N+]([O-])c1ccc(O)cc1I’)+1000000*HIDE_NAME(’nenitzescu’))

Chemical scoring formula: 100*(SMALLER^3),100*(SMALLER^1.5)

Min. search width: 100

Max. reactions per product: 20

Path Score: 20420.95

Umifenovir-3

Analysis type: Automatic Retrosynthesi

Rules: none selected

Filters: Strategies

Max. paths returned: 50

Max. iterations: 1000

Buyable:

1. Max. molecular weight - 1000 g/mol

2. Max. price - 25 $/g

Known:

1. Max. molecular weight - 1000 g/mol

2. Min. popularity - 9999

Inventory:

1. Max. molecular weight - 1000 g/mol

Shorter paths: yes

Avoided SMILES: ’O=C1C=CC(=O)C(Br)=C1’,’CCOC(=O)CC(=O)CCl’,’O=C1C=CC(=O)C=C1’,’O=[N])c1ccc(O)cc1’

Pathway linearity: CONVERGENT

Protecting groups: LESS

Reaction scoring formula: 100*(TUNNEL_COEF*FGI_COEF*20+1000000*(FILTERS+CONFLICT+NON_SELECTIVITY)+40*PROTECT+1000000*HIDE_SMILES(’O=C1C=CC(=O)C(Br)=C1’,’CCOC(=O)CC(=O)CCl’,’O=C1C=CC(=O)C=C1’,’O=[N+]([O-])c1ccc(O)cc1’))

Chemical scoring formula: 100*(SMALLER^3)

Min. search width: 100

Max. reactions per product: 20

Path Score: 7625.04

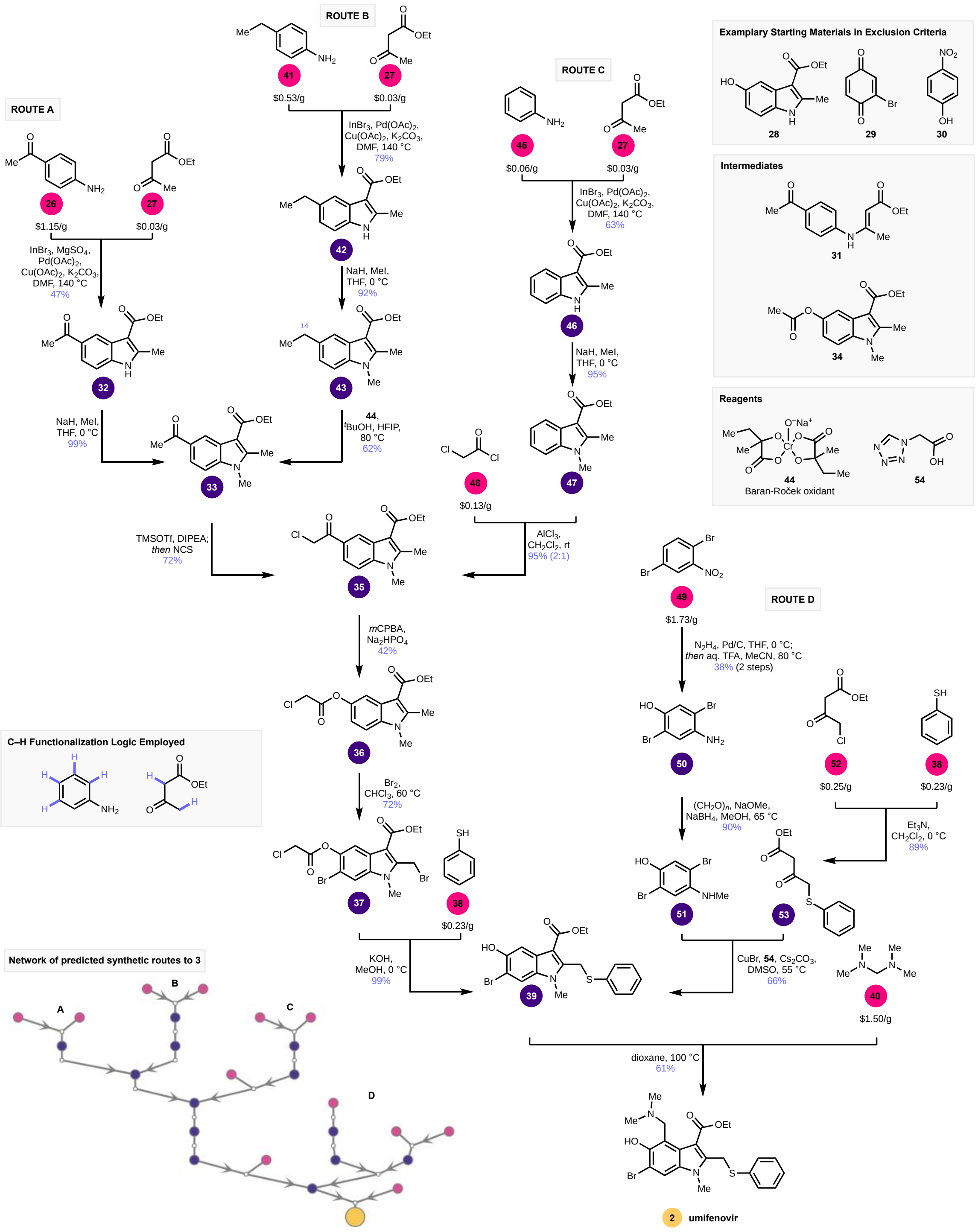

Supplementary Figure 30. Experimental demonstration of four SYNTHIA^{TM} predicted routes to **2**.

Bromhexine-1

Analysis type: Automatic Retrosynthesis

Rules: none selected

Filters: none selected

Max. paths returned: 50

Max. iterations: 5000

Buyable:

1. Max. molecular weight - 1000 g/mol

2. Max. price - 10 $/g

Known:

1. Max. molecular weight - 1000 g/mol

2. Min. popularity - 15

Shorter paths: yes

Avoided SMILES: ’CNC1CCCCC1’

Pathway linearity: COMBO

Protecting groups: LESS

Reaction scoring formula:
100*(20+1000000*(FILTERS+CONFLICT+NON_SELECTIVITY)+40*PROTECT+
1000000*HIDE_SMILES(’CNC1CCC CC1’))

Chemical scoring formula: 100*(SMALLER^3),100*(SMALLER^1.5)

Min. search width: 100

Max. reactions per product: 20

Path Score: 16626.40

Bromhexine-2

Analysis type: Automatic Retrosynthesis

Rules: none selected

Filters: none selected

Max. paths returned: 50

Max. iterations: 2000

Buyable:

1. Max. molecular weight - 1000 g/mol

2. Max. price - 100 $/g

Known:

1. Max. molecular weight - 1000 g/mol

2. Min. popularity - 25

Reaction scoring formula: TUNNEL_COEF*FGI_COEF*20+40*PROTECT+1000000*(CONFLICT+NON_SELECTIVITY+FILTERS)+1000*HIDE_SMILES('BrBr','COC(=O)c1ccccc1N','Nc1c(Br)cc(Br)cc1C=O','CNC1CCCCC1','CS(C)([O])(=O)O[O]','O=C(n1ccnc1)n1ccnc1','O=[N+]([O-])c1ccccc1C Br','NC1CCCCC1')

Chemical scoring formula: SMALLER^3,SMALLER^1.5

Min. search width: 100

Max. reactions per product: 20

Path Score: 20.24

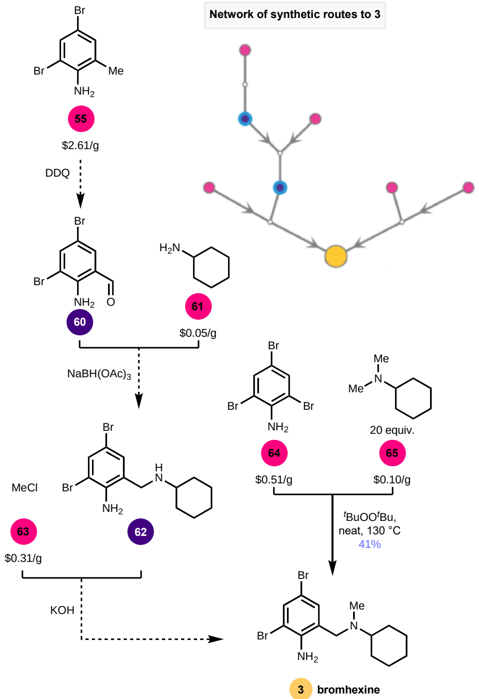

Supplementary Figure 31. SYNTHIA^{TM} predicted and realized routes to **3**. PG = Protection group. SYNTHIA^{TM} recommends when groups should be protected, but does not propose exact protecting groups. In instances where SYNTHIA^{TM} does propose an exact protecting group, the proposed protecting group is drawn.

Galidesivir

Analysis type: Automatic Retrosynthesis

Rules: none selected

Filters: none selected

Max. paths returned: 50

Max. iterations: 5000

Buyable:

1. Max. molecular weight - 1000 g/mol

2. Max. price - 100 $/g

Known:

1. Max. molecular weight - 1000 g/mol

2. Min. popularity - 25

Inventory:

1. Max. molecular weight - 1000 g/mol

Reaction scoring formula: 100*(20+40*PROTECT+100000*(CONFLICT+NON_SELECTIVITY+FILTERS))+ 1000*HIDE_SMILES(’O=c1[nH]cnc2c(C3NC(CO)C(O)C3O)c[nH]c12’,’CC(=O)O.N=CN’,’O=c1[nH]cnc2c([C@@H]3N[C@H](CO)[C@@H](O)[C@H]3O)c[nH]c12’,’CC(=O)OC(C)=O’,’C[N+](C)=CCl.[Cl-]’,’CC#N’,’CC(C)(C)OC(=O)OC(=O)OC(C)(C)C’,’CN(C)C(OC(C)(C)C)N(C)C’,’N#CN’,’O=C(Cl)OCc1ccccc1’,’CC(=O)Nc1ncnc2c(Br)c[nH]c12’,’COC1OC(OC)C(OC(C)=O)C1OC(C)=O’,’N[C@@H](c1ccccc1)c1c(O)ccc2ccccc12’,’c1ccc2[nH]nnc2c1’,’CC(C)(C)C(=O)OC[Mg]Br’,’OC[C@H]1OC(O)[C@H](O)[C@@H]1O’,’C[C@@H](NCc1ccccc1)c1ccccc1’,’CC(C)(C)OC(=O)n1cccc1’,’OC[C@H](O)[C@H]1OC(O)C(O)=C1O’,’Brc1c[nH]c2c(NCc3ccccc3)ncnc12’,’CC(C)(C)OC(=O)N1C=CC[C@H]1CO’,’FB(F)F.N#[N+]c1c[nH]c2c(Cl)ncnc12.[F-]’)

Chemical scoring formula: SMALLER^3,SMALLER^1.5

Min. search width: 150

Max. reactions per product: 25

Path Score: 36793.19

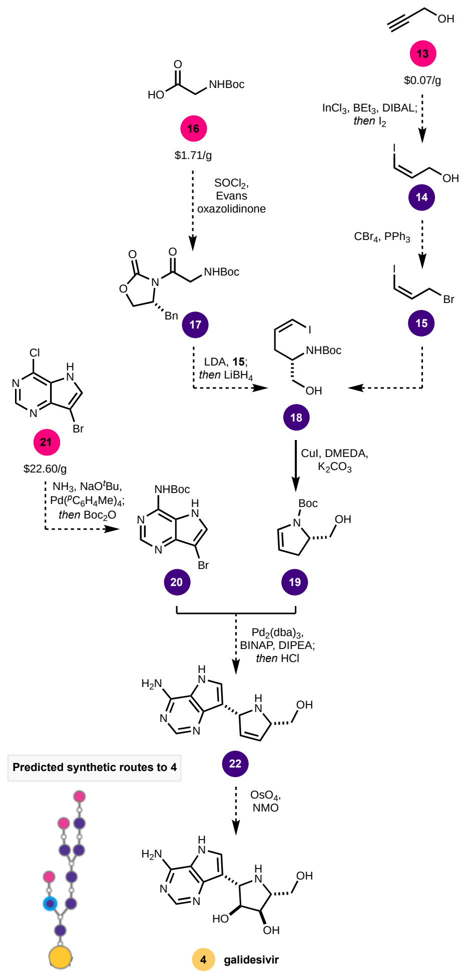

Supplementary Figure 32. SYNTHIA^{TM} predicted routes to **4**. PG = Protection group. SYNTHIA^{TM} recommends when groups should be protected, but does not propose exact protecting groups. In instances where SYNTHIA^{TM} does propose an exact protecting group, the proposed protecting group is drawn.

Ritonavir-1

Analysis type: Automatic Retrosynthesis

Rules: none selected

Filters: Strategies

Max. paths returned: 50

Max. iterations: 5000

Buyable:

1. Max. molecular weight - 1000 g/mol

2. Max. price - 100 $/g

Known:

1. Max. molecular weight - 1000 g/mol

2. Min. popularity - 25

Inventory:

1. Max. molecular weight - 1000 g/mol

Shorter paths: yes

Avoided SMILES: 'CC(=O)[C@@H](Cc1ccccc1)[C@@H](O)C[C@@H](N)Cc1ccccc1','N#C[C@H](C=O)Cc1c]','ICc1ccccc1','N[C@@H](Cc1ccccc1)C[C@H]1CC(=O)C=CO1','O=C1C[C@H](Cc2ccccc2)N1','O=C(Cl)CCc1

Avoided keywords: 'Grignard','Auxiliary'

Pathway linearity: CONVERGENT

Protecting groups: LESS

Reaction scoring formula: 100*(20+1000000*(FILTERS+CONFLICT+NON_SELECTIVITY)+40*PROTECT+1000000*HIDE_SMILES('CC(=O)[C@@H](Cc1ccccc1)[C@@H](O)C[C@@H](N)Cc1ccccc1','N#C[C@H](C=O)Cc1ccccc1','O=C(Cc1ccco1)c1ccccc1','O=CCc1ccccc1','COC(=O)C[C@@H](N)Cc1ccccc1','COC(=O)C(Cc1ccccc1)=[N+]=[N-]','ICc1ccccc1','N[C@@H](Cc1ccccc1)C[C@H]1CC(=O)C=CO1','O=C1C[C@H](Cc2ccccc2)N1','O=C(Cl)CCc1ccccc1','CC(=O)CCc1ccccc1')+1000000*HIDE_NAME('Grignard','Auxiliary'))

Chemical scoring formula: 100*(SMALLER^3)

Min. search width: 100

Max. reactions per product: 20

Path Score: 53645.99

Ritonavir-2

Analysis type: Automatic Retrosynthesis

Rules: none selected

Filters: Strategies

Max. paths returned: 50

Max. iterations: 5000

Buyable:

1. Max. molecular weight - 1000 g/mol

2. Max. price - 100 $/g

Known:

1. Max. molecular weight - 1000 g/mol

2. Min. popularity - 25

Inventory:

1. Max. molecular weight - 1000 g/mol

Shorter paths: no

Avoided SMILES:
’N[C@@H](Cc1ccccc1)C[C@H](O)[C@@H](N)Cc1ccccc1’,’CC(C)(C)OC(=O)N[C@@H](C

Pathway linearity: CONVERGENT

Protecting groups: LESS

Reaction scoring formula:
20+1000000*(FILTERS+CONFLICT+NON_SELECTIVITY)+40*PROTECT+1000000*HIDE_SMILES(’N[C@@H](Cc1cccccc1)C[C@H](O)[C@@H](N)Cc1ccccc1’, ’CC(C)(C)OC(=O)N[C@@H](Cc1ccccc1)C[C@H](O)[C@@H](N)Cc1ccccc1’)

Chemical scoring formula: SMALLER^3

Min. search width: 100

Max. reactions per product: 20

Path Score: 286.87

Ritonavir-3

Analysis type: Automatic Retrosynthesis

Rules: none selected

Filters: Strategies

Max. paths returned: 50

Max. iterations: 5000

Buyable:

1. Max. molecular weight - 1000 g/mol

2. Max. price - 100 $/g

Known:

1. Max. molecular weight - 1000 g/mol

2. Min. popularity - 25

Inventory:

1. Max. molecular weight - 1000 g/mol

Shorter paths: yes

Avoided SMILES: ’O=C(CCl)CCl’

Pathway linearity: CONVERGENT

Protecting groups: LESS

Reaction scoring formula: 100*(20+1000000*(FILTERS+CONFLICT+NON_SELECTIVITY)+40*PROTECT+ 1000000*HIDE_SMILES(’O=C(CCl) CCl’))

Chemical scoring formula: 100*(SMALLER^3)

Min. search width: 100

Max. reactions per product: 20

Path Score: 11541.96

Ritonavir-4

Analysis type: Manual Retrosynthesis

Rules: none selected

Options: none selected

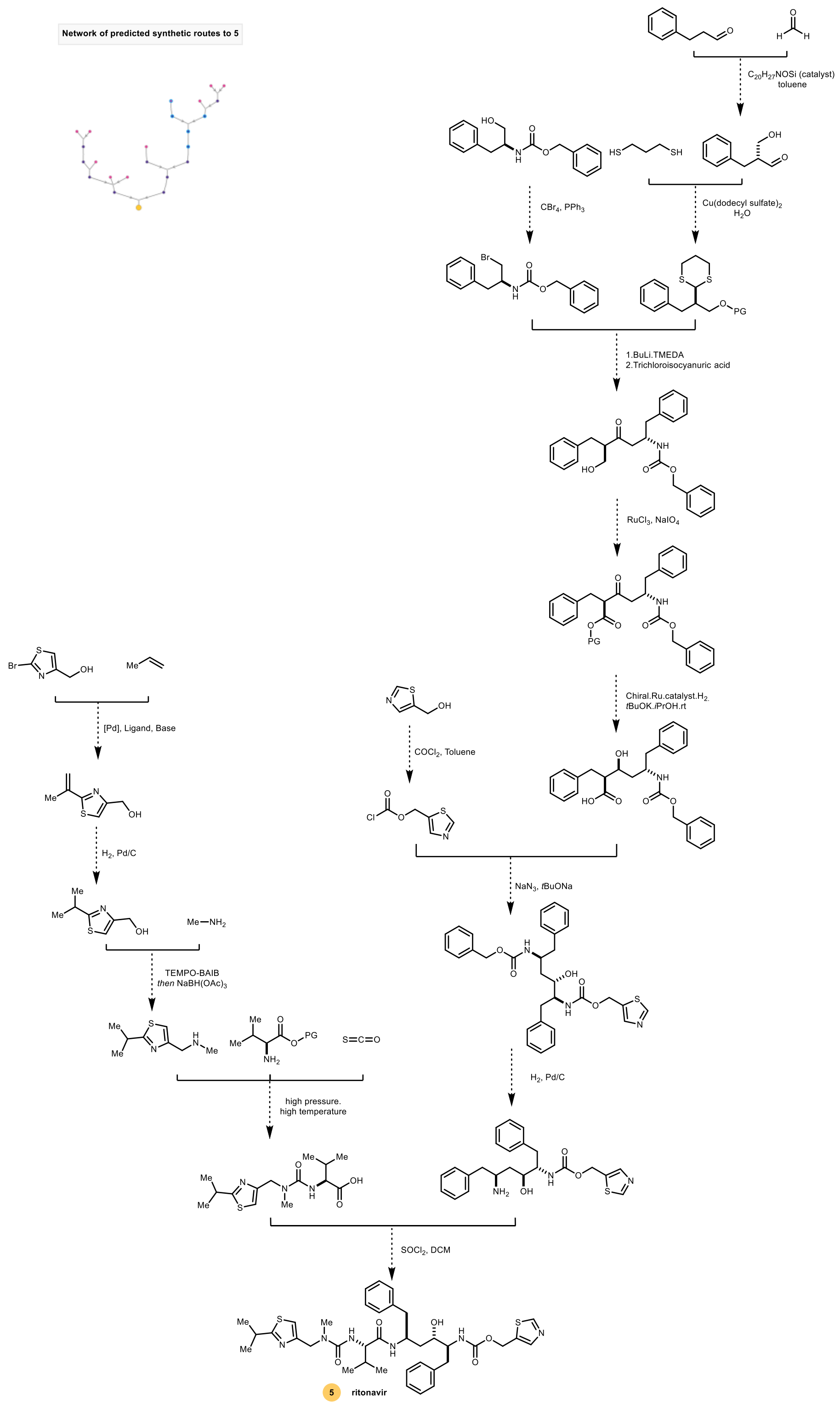

Supplementary Figure 33. SYNTHIA^{TM} predicted routes to **5**. PG = Protection group. SYNTHIA^{TM} recommends when groups should be protected, but does not propose exact protecting groups. In instances where SYNTHIA^{TM} does propose an exact protecting group, the proposed protecting group is drawn.

Cobicistat-1

Analysis type: Automatic Retrosynthesis

Rules: none selected

Filters: Strategies

Max. paths returned: 50

Max. iterations: 5000

Buyable:

1. Max. molecular weight - 1000 g/mol

2. Max. price - 100 $/g

Known:

1. Max. molecular weight - 1000 g/mol

2. Min. popularity - 25

Inventory:

1. Max. molecular weight - 1000 g/mol

Shorter paths: yes Avoided keywords: 'auxiliary','enders'

Pathway linearity: CONVERGENT

Protecting groups: LESS

Reaction scoring formula: 100*(20+1000000*(FILTERS+CONFLICT+NON_SELECTIVITY)+40*PROTECT+ 1000000*HIDE_NAME('auxiliary','enders'))

Chemical scoring formula: 100*(SMALLER^3) Min. search width: 100 Max. reactions per product: 20

Path Score: 25916.09

Cobicistat-2

Analysis type: Automatic Retrosynthesis

Rules: none selected

Filters: none selected

Max. paths returned: 50

Max. iterations: 5000

Buyable:

1. Max. molecular weight - 1000 g/mol

2. Max. price - 100 $/g

Known:

1. Max. molecular weight - 1000 g/mol

2. Min. popularity - 25

Inventory:

1. Max. molecular weight - 1000 g/mol

Shorter paths: yes

Pathway linearity: CONVERGENT

Protecting groups: LESS

Reaction scoring formula: 100*(20+1000000*(FILTERS+CONFLICT+NON_SELECTIVITY)+40*PROTECT)

Chemical scoring formula: 100*(SMALLER^3)

Min. search width: 100

Max. reactions per product: 20

Path Score: 23047.07

Cobicistat-3

Analysis type: Automatic Retrosynthesis

Rules: none selected

Filters: Strategies

Max. paths returned: 50

Max. iterations: 5000

Buyable:

1. Max. molecular weight - 1000 g/mol

2. Max. price - 100 $/g

Known:

1. Max. molecular weight - 1000 g/mol

2. Min. popularity - 25

Inventory:

1. Max. molecular weight - 1000 g/mol

Shorter paths: yes

Avoided SMILES: ’O=C(CCl)CCl’

Pathway linearity: CONVERGENT

Protecting groups: LESS

Reaction scoring formula: 100*(20+1000000*(FILTERS+CONFLICT+NON_SELECTIVITY)+40*PROTECT+1000000*HIDE_SMILES(’O=C(CCl)CCl’))

Chemical scoring formula: 100*(SMALLER^3)

Min. search width: 100

Max. reactions per product: 20

Path Score: 17167.44

Cobicistat-4

Analysis type: Manual Retrosynthesis

Rules: none selected

Options: none selected

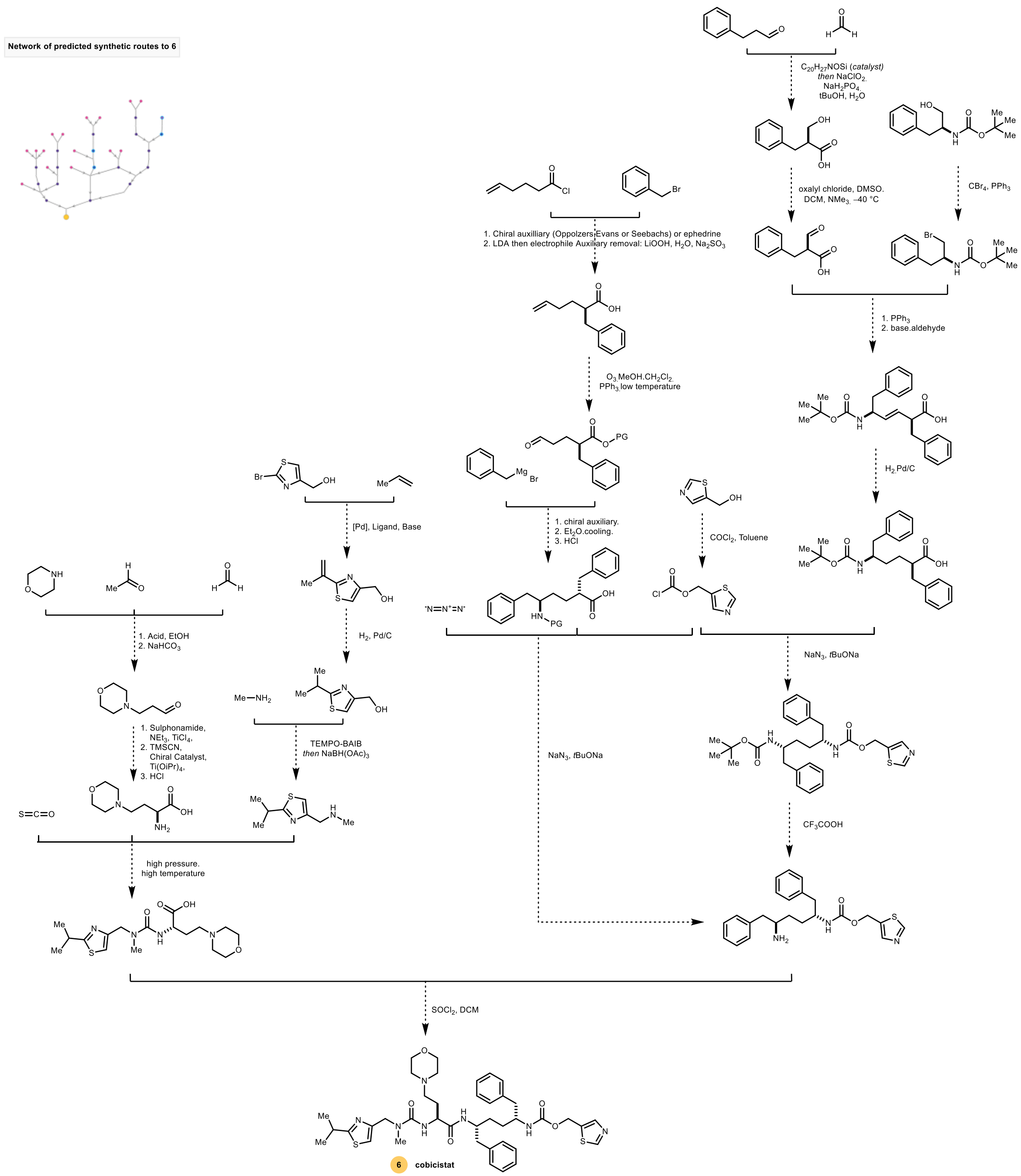

Supplementary Figure 34. SYNTHIA^{TM} predicted routes to **6**. PG = Protection group. SYNTHIA^{TM} recommends when groups should be protected, but does not propose exact protecting groups. In instances where SYNTHIA^{TM} does propose an exact protecting group, the proposed protecting group is drawn.

Ribavirin

Analysis type: Manual Retrosynthesis

Rules: none selected

Options: none selected

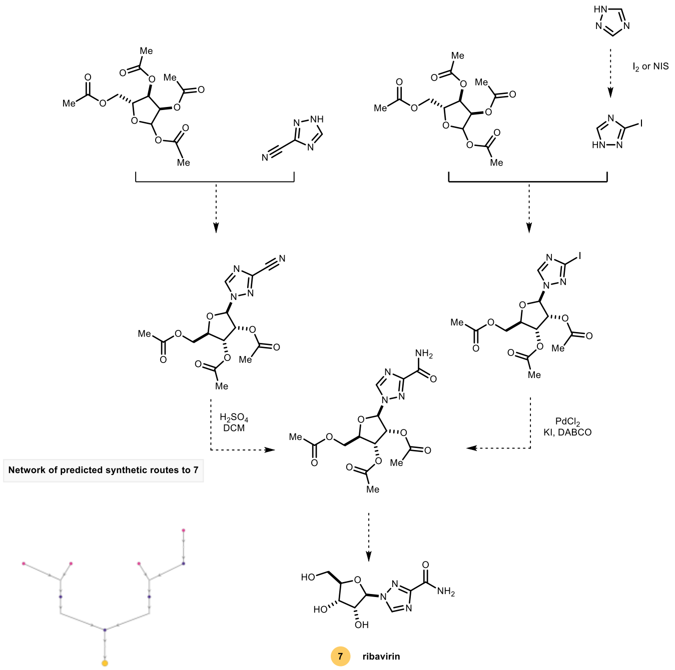

Supplementary Figure 35. SYNTHIA^{TM} predicted routes to **7**.

Camostat-1

Analysis type: Automatic Retrosynthesis

Rules: none selected Filters: none selected

Max. paths returned: 50

Max. iterations: 5000

Buyable:

1. Max. molecular weight - 1000 g/mol

2. Max. price - 100 $/g

Known:

1. Max. molecular weight - 1000 g/mol

2. Min. popularity - 25

Inventory:

1. Max. molecular weight - 1000 g/mol

Reaction scoring formula:
100*(20+1000*HIDE_SMILES('COC(=O)Cc1ccc(O)cc1','O=C(O)Cc1ccc(O)cc1')
+40*PROTECT+100000*(CONFLICT+N ON_SELECTIVITY+FILTERS))

Chemical scoring formula: SMALLER^3,SMALLER^1.5

Min. search width: 100

Max. reactions per product: 20

Path Score: 23562.92

Camostat-2

Analysis type: Automatic Retrosynthesis

Rules: none selected

Filters: none selected

Max. paths returned: 50

Max. iterations: 5000

Buyable:

1. Max. molecular weight - 1000 g/mol

2. Max. price - 100 $/g

Known:

1. Max. molecular weight - 1000 g/mol

2. Min. popularity - 25

Inventory:

1. Max. molecular weight - 1000 g/mol

Reaction scoring formula:
100*(20+1000*HIDE_SMILES('COC(=O)Cc1ccc(O)cc1','O=C(O)Cc1ccc(O)cc1')
+40*PROTECT+100000*(CONFLICT+N ON_SELECTIVITY+FILTERS))

Chemical scoring formula: SMALLER^3,SMALLER^1.5

Min. search width: 100

Max. reactions per product: 20

Path Score: 23562.92

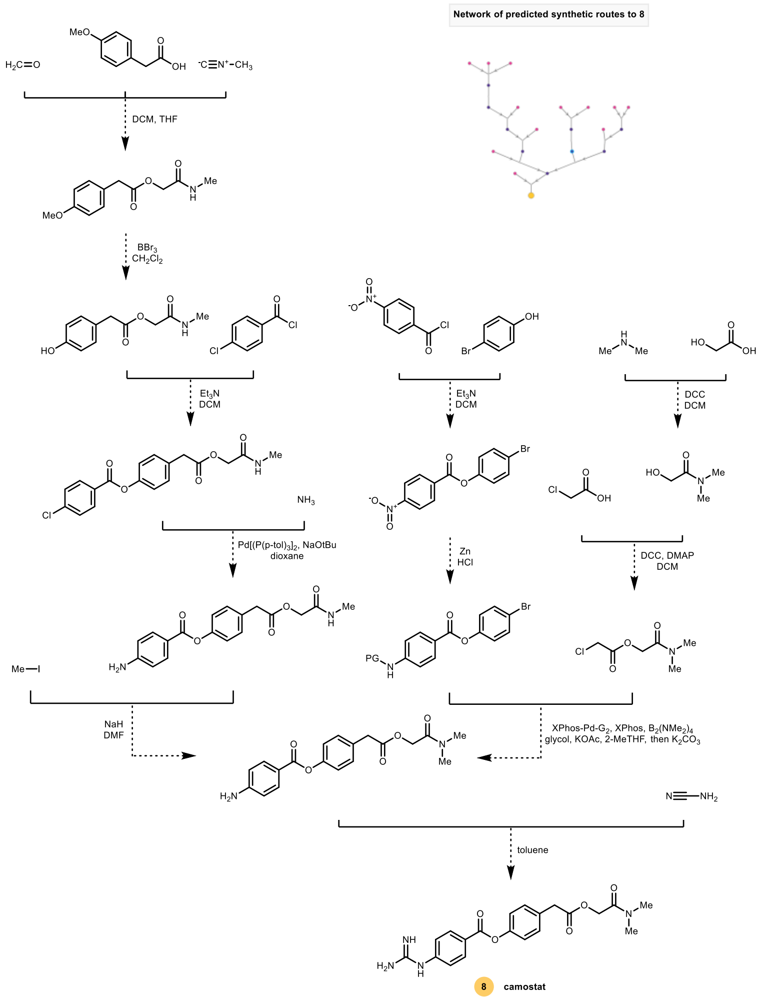

Supplementary Figure 36. SYNTHIA™ predicted routes to **8**. PG = Protection group. SYNTHIA™ recommends when groups should be protected, but does not propose exact protecting groups. In instances where SYNTHIA™ does propose an exact protecting group, the proposed protecting group is drawn.

Darunavir-1

Analysis type: Automatic Retrosynthesis

Rules: none selected

Filters: Strategies

Max. paths returned: 50

Max. iterations: 5000

Buyable:

1. Max. molecular weight - 1000 g/mol
2. Max. price - 100 $/g

Known:

1. Max. molecular weight - 1000 g/mol
2. Min. popularity - 25

Inventory:

1. Max. molecular weight - 1000 g/mol

Shorter paths: yes

Pathway linearity: CONVERGENT

Protecting groups: LESS

Reaction scoring formula:
100*(20+1000000*(FILTERS+CONFLICT+NON_SELECTIVITY)+40*PROTECT)

Chemical scoring formula: 100*(SMALLER^3)

Min. search width: 100

Max. reactions per product: 20

Path Score: 11627.28

Darunavir-2

Analysis type: Manual Retrosynthesis

Rules: none selected

Options: none selected

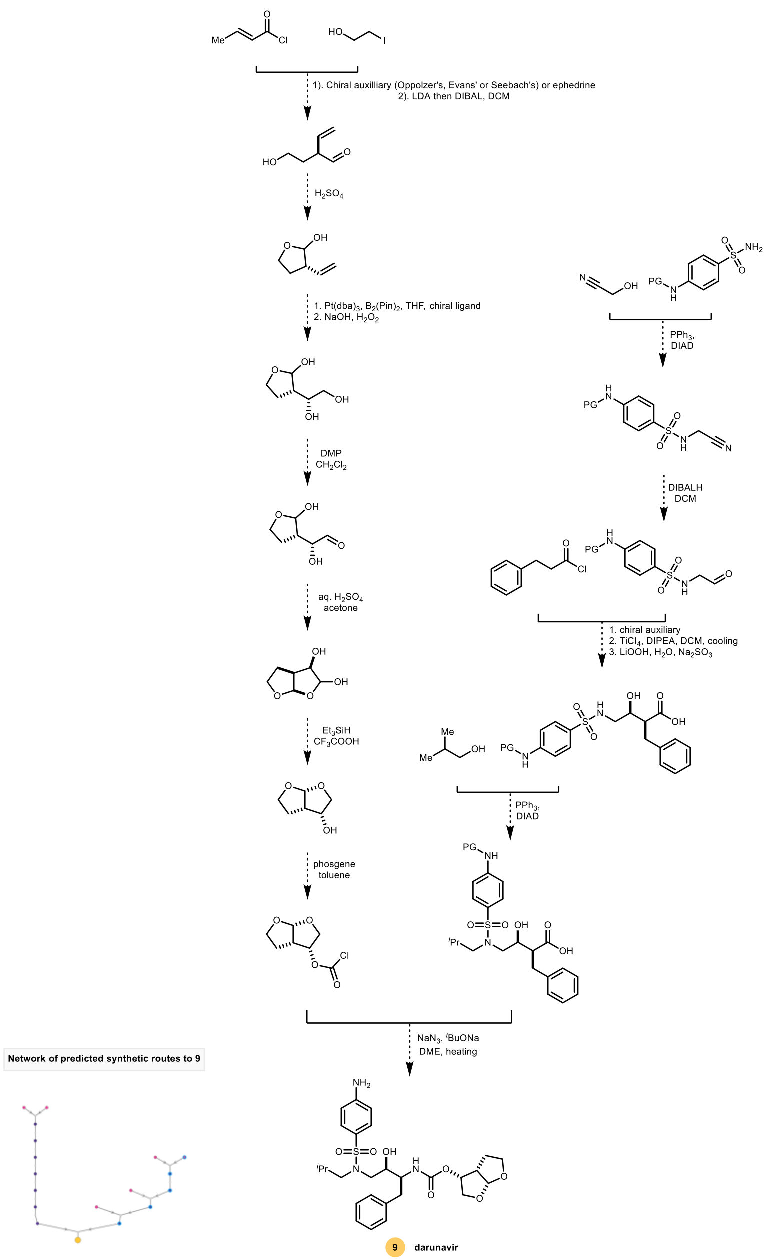

Supplementary Figure 37. SYNTHIA™ predicted routes to **9**. PG = Protection group. SYNTHIA™ recommends when groups should be protected, but does not propose exact protecting groups. In instances where SYNTHIA™ does propose an exact protecting group, the proposed protecting group is drawn.

Nelfinavir

Analysis type: Automatic Retrosynthesis

Rules: none selected

Filters: none selected

Max. paths returned: 50

Max. iterations: 5000

Buyable:

1. Max. molecular weight - 1000 g/mol

2. Max. price - 100 $/g

Known:

1. Max. molecular weight - 1000 g/mol

2. Min. popularity - 25

Inventory:

1. Max. molecular weight - 1000 g/mol

Reaction scoring formula:
20+40*PROTECT+1000000*(CONFLICT+NON_SELECTIVITY+FILTERS)+1000*HIDE_SMILES('CC(C)(C)NC(=O)[C@@H]1C[C@@H]2CCCC[C@@H]2CN1','Cc1c(O)cccc1C(=O)O','Cc1c(O)cccc1 C(=O)Cl','Cc1ccc(S(=O)(=O)[O-])cc1.[NH3+][C@H]1COC[C@@H]1O','C=CC(C=C)NC(=O)c1ccc(OC)cc1','C=C/C=N/[S@@](=O)C(C)(C)C','CS(=O)c1ccccc1','C[C@@H](N[C@H]1COC(C)(C)OC[C@@H]1O)c1ccccc1','CC(=O)Oc1cccc(C(=O)ON2C(=O)CCC2=O)c1C','O=C(O)[C@@H](O)[C@H](O)C(=O)O','C1CC2OC2O1','C[C@H](N)c1ccccc1','C=C[C@H](CO)N1C(=O)c2ccccc2C1=O','CC1(C)OC[C@H](C=O)O1','COC(=O)[C@@H]1COC(=O)N1','O=C(C Cl)O[Na]','O=C(N[C@H]1COC1=O)OCc1ccccc1')

Chemical scoring formula: SMALLER^3,SMALLER^1.5

Min. search width: 100

Max. reactions per product: 20

Path Score: 1278.81

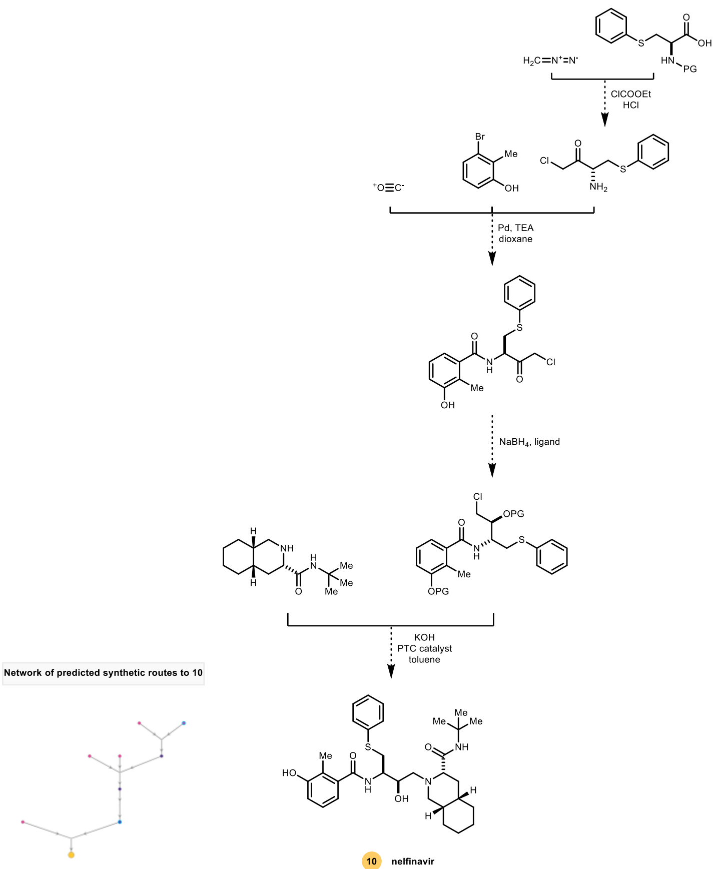

10 nelfinavir

Supplementary Figure 38. SYNTHIA™ predicted routes to **10**. PG = Protection group. SYNTHIA™ recommends when groups should be protected, but does not propose exact protecting groups. In instances where SYNTHIA™ does propose an exact protecting group, the proposed protecting group is drawn.

Favipiravir-1

Analysis type: Automatic Retrosynthesis

Rules: none selected

Filters: none selected

Max. paths returned: 50

Max. iterations: 5000

Buyable:

1. Max. molecular weight - 1000 g/mol

2. Max. price - 100 $/g

Known:

1. Max. molecular weight - 1000 g/mol

2. Min. popularity - 25

Inventory:

1. Max. molecular weight - 1000 g/mol

Reaction scoring formula: 20+40*PROTECT+1000000*(CONFLICT+NON_SELECTIVITY+FILTERS)+1000 *HIDE_SMILES('CCOC(=O)C(OCC)OCC','N#CCN.N#CCN.O=S(=O)(O)O','COC(=O)C(=O)OC','Nc1cnccn1','Nc1nccnc1C(=O)O','NC(=O)c1cnccn1','CCOC(=O)C([NH3+])C(=O)OCC.[Cl-]','O=CC=O')

Chemical scoring formula: SMALLER^3,SMALLER^1.5

Min. search width: 100

Max. reactions per product: 20

Path Score: 155.83

Favipiravir-2

Analysis type: Automatic Retrosynthesis

Rules: none selected

Filters: Strategies

Max. paths returned: 50

Max. iterations: 5000

Buyable:

1. Max. molecular weight - 1000 g/mol

2. Max. price - 100 $/g

Known:

1. Max. molecular weight - 1000 g/mol

2. Min. popularity - 9999

Inventory:

1. Max. molecular weight - 1000 g/mol

Shorter paths: yes

Pathway linearity: CONVERGENT

Protecting groups: NONE

Reaction scoring formula: 100*(20+1000000*(FILTERS+CONFLICT+N
ON_SELECTIVITY)+1000000*PROTECT)

Chemical scoring formula: 100*(SMALLER^3)

Min. search width: 100

Max. reactions per product: 20

Path Score: 22518.43

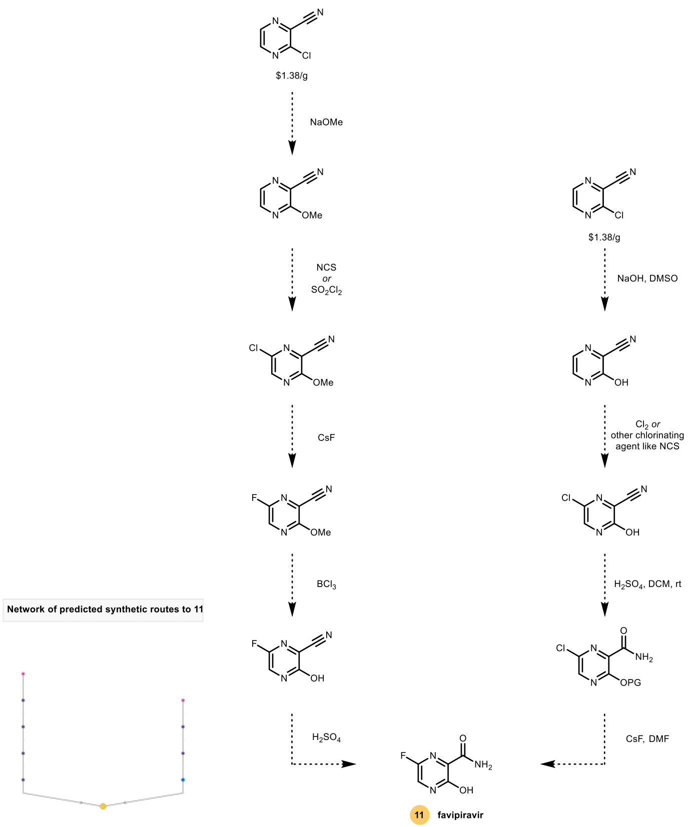

Supplementary Figure 39. SYNTHIA™ predicted routes to **11**. PG = Protection group. SYNTHIA™ recommends when groups should be protected, but does not propose exact protecting groups. In instances where SYNTHIA™ does propose an exact protecting group, the proposed protecting group is drawn.

Bariticinib

Analysis type: Automatic Retrosynthesis

Rules: none selected

Filters: none selected

Max. paths returned: 50

Max. iterations: 5000

Buyable:

1. Max. molecular weight - 1000 g/mol

2. Max. price - 100 $/g

Known:

1. Max. molecular weight - 1000 g/mol

2. Min. popularity - 25

Inventory:

1. Max. molecular weight - 1000 g/mol

Reaction scoring formula: 20+40*PROTECT+1000000*(CONFLICT+NON_SELECTIVITY+FILTERS)+1000*HIDE_SMILES('NCCn1cccn1','c1cn[nH]c1','Cn1cccn1','CS(N)(=O)=O')

Chemical scoring formula: SMALLER^3,SMALLER^1.5

Min. search width: 100

Max. reactions per product: 20

Path Score: 146.27

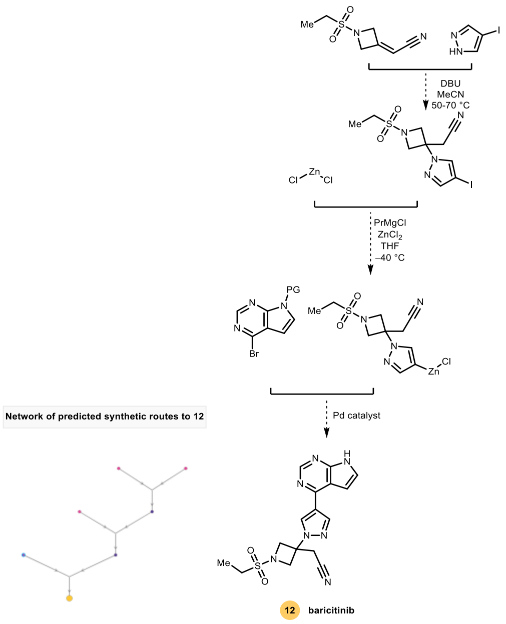

Supplementary Figure 40. SYNTHIA™ predicted routes to **12**. PG = Protection group. SYNTHIA™ recommends when groups should be protected, but does not propose exact protecting groups. In instances where SYNTHIA™ does propose an exact protecting group, the proposed protecting group is drawn.

Supplementary References

1. Wurtz, S., Rakshit, S., Neumann, J. J., Droge, T. & Glorius, F. Palladium-Catalyzed Oxidative Cyclization of *N*-Aryl Enamines: From Anilines toIndoles. *Angew. Chem. Int. Ed.* 47, 7230-7233 (2008).
2. Rodriguez, R. A., Pan, C.-M., Yabe, Y., Kawamata, Y., Eastgate, M. D. & Baran, P. S. Palau'chlor: A Practical and Reactive Chlorinating Reagent. *J. Am. Chem. Soc.* 136, 6908-6911 (2014).
3. Nakatsuka, S., Osamu, A., Ueda, K. & Goto, T. Introduction of a hydroxy group onto 5- and 6-position of indole nucleus by Friedel-Crafts acylation and subsequent Baeyer-Villiger Oxidation. *Heterocycles* 26, 1471-1474 (1987).
4. Trofimov, F. A., Tsyshkova, N. G. & Zotova, S. A. Synthesis of a new antiviral agent, arbidole. *Pharm. Chem. J.* 27, 75-76 (1993).
5. Kanda, Y., Nakamura, H., Umemiya, S., Puthukanoori, R. K., Appala, V. R. M., Gaddamanugu, G. K., Paraselli, B. R. & Baran, P. S. Two-Phase Synthesis of Taxol. *J. Am. Chem. Soc.* 142, 10526-10533 (2020).
6. Riesco-Domínguez, A., van de Wiel, J., Hamlin, T. A., van Beek, B., Lindell, S. D., Blanco-Ania, D., Bickelhaupt, F. M. & Rutjes, F. P. J. T. Trifluoromethyl Vinyl Sulfide: A Building Block for the Synthesis of CF_3S-Containing Isoxazolidines. *J. Org. Chem.* 83, 1779-1789 (2018).

7. de Fonzo, N., Quartarone, G., Ronchin, L., Tortato, C. & Vavasori, A. Kinetics and mechanistic study of the Bamberger rearrangement of *N*-phenylhydroxylamine to 4-aminophenol in acetonitrile-trifluoroacetic acid: A substrate acid complex as para selectivity driver. *Applied Catalysis A: General* 516, 58–69 (2016).
8. Zhang, W., Oya, S., Kung, M. P., Hou, C., Maier, D. L. & Kung, H. F. F-18 Stilbenes as PET Imaging Agents for Detecting β-Amyloid Plaques in the Brain. *J. Med. Chem.* 48, 5980–5988 (2005).
9. Chen, T., Benmohamed, R., Arvanites, A. C., Ranaivo, H. R., Morimoto, R. I., Ferrante, R. J., Watterson, D. M., Kirsch, D. R. & Silverman, R. B. Arylsulfanyl pyrazolones block mutant SOD1-G93A aggregation. Potential application for the treatment of amyotrophic lateral sclerosis. *Bioorg. & Med. Chem.* 19, 613-622 (2011).
10. Liu, X.-G., Li, Z.-H., Xie, J.-W., Liu, P., Zhang, J. & Dai, B. Copper-catalyzed synthesis of 2,3-disubstituted indoles from ortho-haloanilines and β-keto esters/β-diketone. *Tetrahedron* 72, 653-657 (2016).
11. Wright, Z. V. F., Wu, N. C., Kadam, R. U., Wilson, I. A. & Wolan, D. W. Structure-based optimization and synthesis of antiviral drug Arbidol analogues with significantly improved affinity to influenza hemagglutinin. *Bioorg. & Med. Chem.* 27, 3744-3748 (2017).
12. Ueno, R., Ikeda, Y. & Shirakawa, E. *tert*-Butoxy-Radical-Promoted α-Arylation of Alkylamines with Aryl Halides. *Eur. J. Org. Chem.* 2017, 4188-4193 (2017).
